# Public surface disinfection every 2 hours can reduce the infection risk of norovirus in airports up to 83%

**DOI:** 10.1371/journal.pcbi.1012561

**Published:** 2024-12-05

**Authors:** Nan Zhang, Linan Zhuang, Marco-Felipe King, Hua Qian, Min Zhu

**Affiliations:** 1 Beijing Key Laboratory of Green Built Environment and Energy Efficient Technology, Beijing University of Technology, Beijing, China; 2 School of Civil Engineering, University of Leeds, Woodhouse Lane, Leeds, United Kingdom; 3 School of Energy and Environment, Southeast University, Nanjing, China; 4 6th Medical Center of General Hospital of PLA, Beijing, China; The University of Hong Kong, CHINA

## Abstract

Norovirus, primarily transmitted via fomite route, poses a significant threat to global public health and the economy. Airports, as critical transportation hubs connecting people from around the world, has high potential risk of norovirus transmission due to large number of public surfaces. A total of 21.3 hours of video episodes were recorded across nine functional areas at the airport, capturing 25,925 touches. A surface transmission model based on a Markov chain was developed. Using the beta-Poisson dose-response model, the infection risk of norovirus and the effectiveness of various interventions in different airports’ areas were quantified. Without any preventive measures, restaurants at airports exhibited the highest risk of norovirus transmission, with an infection probability of 8.8×10^−3^% (95% CI, 1.5×10^−3^% -2.1×10^−2^%). This means approximately 4.6 (95% CI, 0.8–10.9) out of 51,494 passengers who entered the restaurants would be infected by an infected passenger. Comparing with no surface disinfection, disinfecting public surfaces every 2 hours can reduce the risk of norovirus infection per visit to the airport by 83.2%. In contrast, comparing with no hand washing, handwashing every 2 hours can reduce the infection risk per visit to the airport by only 2.0%, making public surface disinfection significantly more effective than handwashing. If the mask-wearing rate increases from 0% to 50%, the infection risk of norovirus would be decreased by 48.0% (95% CI, 43.5–52.3%). Furthermore, using antimicrobial copper/copper-nickel alloy coatings for most public surfaces could reduce the infection risk by 15.9%-99.2%.

## Author summary

The occurrence of norovirus outbreaks during air travel is common. Previous studies have predominantly focused on aircraft cabins, but airports, serving as connectivity nodes for various cities, deserve equal attention. Norovirus primarily spreads through surface transmission, and within airports, the frequent interactions among passengers and their touch with public surfaces offer potential pathways for norovirus to spread via contaminated surfaces. To investigate the risk of norovirus infection among passengers through surface transmission at airports, we collected real touch data from passengers in different zones of the airport. The aim of this study was to quantify the risk of norovirus transmission through surface touch in different airport areas and evaluate the effectiveness of various interventions targeting surface transmission.

The study identified airport restaurants as high-risk locations for norovirus transmission via surface transmission. Simulation results indicated that public surface disinfection, mask-wearing, and the use of antimicrobial surfaces are effective interventions for controlling the spread of norovirus via surface transmission. These findings provide crucial insights for developing infection prevention and control strategies specifically tailored for norovirus within airport environments.

## 1. Introduction

Norovirus serves as a primary cause of viral gastroenteritis outbreaks, accounting for approximately 685 million cases each year and resulting in over 200,000 deaths [1,2]. The global economic burden of norovirus is substantial, with annual losses reaching as high as $64.5 billion due to healthcare expenses and productivity losses [3]. Immunity to norovirus in humans is typically short-lived. The duration of immunity to norovirus (NoV) gastroenteritis ranges from 6 months to 2 years, meaning individuals may experience multiple infections over their lifetime [4,5].

Norovirus primarily spreads through the fecal-oral, vomit-oral, and surface touch routes, as well as via contaminated food, water, and the environment [6–9]. The viral concentration in the feces of norovirus patients can reach up to 10^11^ genomic copies/g [10]. However, only 18 to 1000 infectious norovirus particles may be required to infect susceptible individuals [11]. Norovirus exhibits considerable stability in the environment, surviving for up to two weeks on surfaces and even two months in water [12].

Numerous reports of norovirus outbreaks have highlighted the significant role of contaminated surfaces in transmission. For instance, a case report mentioned an incident where seven soccer players were infected with norovirus due to vomit-contaminated food bags [13]. Furthermore, during a long-haul flight, after a passenger experienced a vomiting event, 27 different crew members on the same plane became infected with norovirus over the following five days [14]. In schools, 22% of frequently touched surfaces have tested positive for norovirus genome copies using RT-PCR technology [15]. Therefore, the transmission route of norovirus through contaminated surfaces deserves significant attention.

Airports are critical transportation hubs connecting people from around the world, thus significantly increasing the potential for cross-infection. Before the COVID-19 pandemic, in 2018, the global aviation industry transported 12 million passengers every day [16]. However, the indoor environment with high-density passengers also facilitates disease transmission. A previous study revealed that 10% of frequently touched surfaces at airports (e.g. lounge chairs, stair handrails, and luggage trays) were contaminated with at least one respiratory virus, such as rhinovirus [17]. When passengers touch contaminated surfaces and then contact their facial mucous, there is a risk of infection. Therefore, assessing infection risk in different areas at airports and formulating effective strategies on norovirus prevention and control are important, which also critical for aviation industry’s recovery.

In researching the surface transmission of pathogens, scientists often employ tools such as Markov chain models and multi-agent models to simulate the viral exposure risk [18–21]. However, these models often inadequately account for the real touch behaviors, even though human behavior plays a crucial role in the transmission of infectious diseases [22].

Quantitative Microbial Risk Assessment (QMRA) is a technique used to simulate the infection risk in populations exposed to microorganisms, following a four-step process: hazard identification, exposure assessment, dose-response, and risk characterization [23]. QMRA can quantify infection risks across various scenarios, aiding in the estimation of infection risks and effectiveness of interventions [24]. In this study, to more accurately assess the risk of norovirus infection among airport passengers in different regions through fomite transmission, we collected real human surface touching behavior data and developed a fomite transmission model based on Markov chains to evaluate passengers’ viral exposure. Subsequently, using the dose-response model, we quantitatively calculated the infection risk of norovirus among passengers and assessed the effectiveness of various interventions. This study provides a scientific support for strategy making on infectious disease prevention and control at airports.

## 2. Method

### 2.1. Ethics statement

Both the experiments and survey received approval from the Ethics Committee of Beijing University of Technology (No. BJUT-CJXB03). In China, video recording in public areas was allowed, and we had deleted all videos after data collection on surface touch behaviors. Considering large number of passengers, we did not receive the formal consent from all participants before the video recording.

### 2.2. Data collection and analysis

In this study, we focused nine typical areas within the airport: manual check-in, self-service check-in, escalators, restaurants, charging stations, shops, waiting areas, boarding areas, and baggage claim [25]. Using norovirus as an example, we simulated virus transmission among passengers through surface contact in the aforementioned functional areas and evaluated the potential effectiveness of interventions, including mask wearing, handwashing, public surface disinfection, and the installation of antimicrobial surfaces, based on our simulation results. The methods of the study could be expanded to risk analysis on fomite transmission of all pathogens.

Six postgraduate students in our research groups were asked to collect a total of 21.3 hours of video recordings of passenger touch behavior in nine functional areas of two northern airports and one southern airport in China, from August to November 2022 (during the COVID-19 pandemic). Experiment participants used tripods to fix the recording devices (e.g., smartphones or GoPro). The recording angles typically captured passengers’ actions from the side to minimize obstructions of their hands. All passengers were anonymous in the study and video collection in public areas was allowed in China. Considering a large number of passengers and keep the real behaviors of passengers without interference, no formal consent was conducted by these passengers.

Six trained video analysts performed a second-by-second analysis of touch behaviors in the video episodes. The collected data on touch behaviors included the surface touched by each passenger’s left or right hand and whether they were wearing masks (We recorded mask-wearing status and conducted a further analysis assuming passengers do not wear masks and examining their mucous membrane touch.) (S1 Table). Video analysists tracked individuals by assigning numerical codes when they first appeared in the video, and if they left and re-entered, they were not counted as new but continued to be tracked until the video’s end. A touch was defined as the initiation of hand contact with an object’s surface until departure, with a time precision of 1 second (e.g., if a hand touched the screen twice in one second, it was only recorded as one touch) [19]. Finally, 25,925 surface touch events were collected (S2 Table).

In the nine airport areas, we collected touch behavior data from 1,760 passengers (S3 Table). There were a total of 108 surfaces touched across all areas, categorized into four primary categories: facial mucous (eyes, nose, and mouth), body (hands, head/neck, torso, arms, legs), private surfaces (e.g., phones, boarding passes), and public surfaces (e.g., check-in counters, restaurant tables) (S4 Table). In this study, surface touch frequency is defined as the total number of touches on a surface in a specific area divided by the total time that all passengers spent in that area. Mask-wearing rate was defined as the time passengers wore masks in an area divided by the total time all passengers spent in that area.

Flight data for a northern airport on a workday in April 2022 were collected from a flight ticket website [26]. On that day, the national pandemic situation was not very severe, and it had a relatively minor impact on people’s travel behavior. This allowed the data to reliably reflect the passenger traffic at that time. Flight data included departure times and aircraft models (e.g. Boeing 737). There were a total of 383 direct flights from this airport on that day. By searching for the aircraft models, number of seats could be obtained. Assuming all flights were at full capacity, we estimated that the airport received a maximum of 67,050 passengers that day. Additionally, we collected 1,536 valid survey responses through an online questionnaire system (https://www.wjx.cn) (S1 Text). The questionnaire covered the total time passengers spent at the airport, whether and how long they stayed in the nine areas, passenger preferences for check-in pattern (manual or self-service), and the number of people travelled together. The results of the passenger questionnaire on stay probabilities and durations in different areas are listed in S5 Table.

Using flight information (aircraft departure time) and the total time passengers spent at the airport, we could determine when passengers entered the airport. For example, if a flight departed at 9:30 a.m., and a passenger spent 2 hours at the airport, we would assume that the passenger entered the airport at 7:30 a.m.

### 2.3. Airport surface transmission model

In this study, a first-order discrete-time Markov chain model was used to simulate human touch behaviors. Given the current state, the future and past surfaces touched are independent (Eq 1) [18].

$$p_{i,j}=P(X_{n+1}=j|X_{n}=i)$$ (1)

where, *X*_*n*_ represents the *n*^th^ touch, *i* and *j* represent the surfaces of the n^th^ and n+1^th^ touched, respectively; *P*_*i*,*j*_ represents the probability of touching surface *j* after touching surface *i*.

The model utilized real human touch behavior data collected from various areas of the airport as input parameters. The transition probabilities of the Markov chain model were determined through the following steps: (1) We collected passengers’ touch behavior data from the videos, recording the start and end times of each touch, and which surface is touched (when there is no touch, it is considered as touching an empty surface, with a surface code of -1 (S2 Table)). (2) In the Markov chain model, each state corresponded to a touched surface and the duration of the touch. For example, a left hand touched an escalator handrail for 10 seconds. (3) For each pair of states (i, j), we counted the number of transitions from state i to state j. Assuming the observed sequence is X_1_, X_2_,…, X_n_, where X_k_ represents the state of the k-th touch. If state i occurs and is followed by state j, it is recorded as one transition from i to j. (4) The transition probability of P_i,j_ was the ratio of the number of transitions from state i to state j to the total number of times starting from state i.

Fomite transmission is influenced by various factors such as inactivation rate on different surfaces [27]. Simultaneously, during the process of hand-to-surface contact, the type of surface also affects the virus transfer rate between hands and surfaces (i.e., the proportion of virus transfer from hands to surfaces or from surfaces to hands with each touch) [28]. In our investigation of surface transmission, all abovementioned factors were considered to calculate the exposure of norovirus via the surface transmission route, and relevant parameters are listed in S4 Table. The surface transmission model is described by Eq (2) [29].

$$\left\{ \begin{array}{l} V_{s}(t+1)=V_{s}(t)+\sum_{i_{s}(t)}^{n_{s}(t)}\left[A_{hs}(\frac{V_{h}(t)}{A_{h}}r_{hs}-\frac{V_{s}(t)}{A_{s}}r_{sh})]-\frac{I_{ac-s_{i}}}{60}V_{s}(t\right)\\ V_{h}(t+1)=V_{h}(t)+A_{hs}(\frac{V_{s}(t)}{A_{s}}r_{sh}-\frac{V_{h}(t)}{A_{h}}r_{hs})-\frac{I_{ac-h}}{60}V_{h}(t) \end{array}\right.$$ (2)

where *h* and *s* correspond to the hand and the surface, *V*_*h*_(*t*) and *V*_*s*_(*t*) denote the viral loads on the hand and the surface at a given time *t*. *A*_*s*_ and *A*_*h*_ represent the total surface and hand areas, respectively. *A*_*hs*_(*t*) stands for the effective touch area between the hand and the surface, *r*_*sh*_ and *r*_*hs*_ describe the rates of virus transfer from the surface/hand to the hand/surface. *i*_*s*_(*t*) indicates the specific surface touched by the hand at that particular moment, *n*_*s*_(*t*) reflects the total count of surfaces touched by hands at time *t*, and *I*_*ac−s*_ and *I*_*ac−h*_ signify the inactivation rates (min^-1^) of the virus on the surface and hands.

### 2.4. Dose-response model

We assume that the first passenger, who is during the period with the highest infectivity (with a norovirus content in their feces of 2.5×10^11^ genome copies/g) and exhibits symptoms of diarrhea, entering a specific airport area [10]. It is assumed that 0.001g of feces left on hands of the infected after toileting [24]. When fingers come into contact with the nose or mouth, 34% of the particles on the hands are transferred to the nose or mouth [29,30]. The viral exposure refers to the total amount of virus transferred from the hands to the susceptible person’s oral and nasal mucous membranes.

We assume that norovirus patients may have fecal contamination on their hands after a bout of diarrhea. However, it should be noted that norovirus patients may experience frequent diarrhea during their infectious period, with rates as high as 1.6 times per hour in severe cases [31]. This implies that infected individuals may get their hands contaminated with feces multiple times, subsequently spreading the virus to surfaces and increasing the risk of infection for susceptible individuals.

The frequency of mucous touch is a key factor for virus transmission via fomite route. In this study, touch behaviors of passengers were collected during the pandemic. China implemented mandatory mask-wearing measures in public indoor environments, leading to widespread mask-wearing among airport passengers [32]. Wearing masks can significantly reduce the frequency of passengers touching mucous membranes [33]. To obtain the real frequency on mucous touch, it is assumed that passengers touching the outer surface of the mask in the non-edge areas when wearing a mask is regarded as touching the nose/mouth. After considering the mucous membrane contact frequency, it’s important to note that the percentages of people touching their eyes, nose, and mouth differ. We referenced a previous study and allocated the percentages of touching the eyes, nose, and mouth as 8.1%, 42.8%, and 49.1%, respectively [34].

The beta-Poisson dose-response model mathematically links viral exposure to the probability of infection, as shown in Eq (3):

$$p=1-{\left(1+n*dose\right)}^{-r}$$ (3)

based on previous research on the dose-response parameters of norovirus, the values of *n* was set to be 2.55×10^−3^ and *r* was set to be 0.086 [11,24,35].

The infection risk for each passenger was determined using the dose-response equation. We define the number of infections as the number of passengers multiplied by the estimated infection risk.

### 2.5. Intervention measures

Handwashing, public surface disinfection, mask-wearing, and the use of antimicrobial surfaces are common interventions for infectious disease transmission via fomite route. Here are the assumptions for these interventions in the model:

Handwashing and public surface disinfection: the model assumes that handwashing and disinfection of public surfaces occur at frequencies of 0.2/0.5/1/2 times per hour, resulting in a 99.99% reduction in viral load on hands/public surfaces [36–38]. For instance, if a frequency of 0.2 times per hour was set, the viral load on passenger’s hands or public surfaces would be reduced by 99.99% every 5 hours.

Mask-wearing: When they touch their nose and mouth, it is considered as touching the outer surface of the mask.

Antimicrobial surfaces: antimicrobial surfaces could be installed in the public areas of the airport (S6 Table), and two antimicrobial materials (pure copper and copper-nickel alloy) were considered. Pure copper achieves a norovirus deactivation rate of 0.3 min^-1^, and a copper-nickel alloy containing 70% copper achieves a norovirus deactivation rate of 0.07 min^-1^ [39].

The parameters involved in the model and their respective assumptions are listed in Table 1.

**Table 1:** Model parameters.

Description	Units	Value	Reference
**Virus shedding volume**			
Virus concentration in feces	genome copies/g	2.5×10^11^	[10]
Feces transferred to hands during wiping process	g	1.0×10^−3^	[24]
**Transfer rate (hand to surface/ surface to hand)**			
Mucous membranes	Proportion	0.34/0	[30]
Skin		0.18/0.18	[40]
fabric/cloth		0.67/0.01	[41]
Glass		0.19/0.18	[42]
Porous/non-porous		0.46/0.05	[43,44]
Non-porous		0.12/0.07	[45,46]
**Inactivation rate**			
Skin	min^-1^	0.040	[47]
fabric/cloth		0.008	[20]
Glass		0.002	[48]
Porous/non-porous		0.008	[20]
Non-porous		0.002	[48]
**Prevention and control strategies**			
Hand washing/disinfection efficiency	Proportion	0.99	[49]
Antibacterial surface inactivation rate-copper	min^-1^	0.3	[39]
Antibacterial surface inactivation rate-copper-nickel		0.07	
**beta-Poisson dose-response model**			
dose-the amount of virus entering the mucosa of susceptible individuals is equal to	genome copies	*V*_*s*_(*t*+1) in Eq (2)	-
*n* = dose–response constant	-	2.55×10^−3^	[24,35]

### 2.6. Statistical analysis

The data in the results section are presented using means and 95% confidence intervals (95% CI). Statistical significance was analyzed by one-way ANOVA followed by Tukey’s post hoc test using SPSS statistics version 26.0 software. A value of P < 0.05 was considered statistically significant.

## 3. Results

### 3.1. Mucosal touch behavior

Passengers in restaurants had the highest touch frequency on facial mucous (10.3 times/h), followed by charging areas (4.6 times/h) and shopping areas (2.2 times/h). In other areas, the touch frequency on facial mucous was less than 2 times/h. The lowest mask-wearing rate was observed in restaurants, at only 22.0%, followed by the waiting area (87.3%) and the charging area (90.6%). The average mask-wearing rate among passengers in airport areas is 87.9% (95%CI, 35.1%-99.9%) (Table 2).

**Table 2:** Touch frequency on facial mucous and mask-wearing rate of passengers in different areas.

Airport area	Touch frequency on facial mucous^1^ (times/h)	Mask wearing rate^2^ (%)	Mask touch frequency^3^ (times/h)	Ratio of touches on the outer edge of the mask ^4^ (%)
Manual check-in	1.1	98.5	11.2	52.0
Self-service check-in	0.5	95.6	8.2	71.4
Escalator	1.1	98.9	10.7	16.0
Restaurant	10.3	22.0	14.5	92.1
Charging area	4.6	90.6	15.0	50.6
Shopping area	2.2	98.2	13.4	81.3
Waiting area	1.9	87.3	13.3	88.2
Boarding area	1.3	100.0	8.2	25.0
Baggage claim area	0	99.7	1.6	33.3

^1^ Touch frequency on facial mucous: ratio of the total times all passengers touched the facial mucosal to the total time of all passengers stayed in the area.

^2^Mask wearing rate: ratio of the time all passengers wore masks to the total time of all passengers stayed in the area.

^3^Mask touch frequency: ratio of the total times all passengers touched the mask to the total time of all passengers stayed in the area.

^4^Ratio of touches on the outer edge of the mask: ratio of the total times all passengers touched the outer edge of the mask to the total times they touched the mask.

### 3.2. The viral load transferred from surfaces to hands

**Fig 1:**
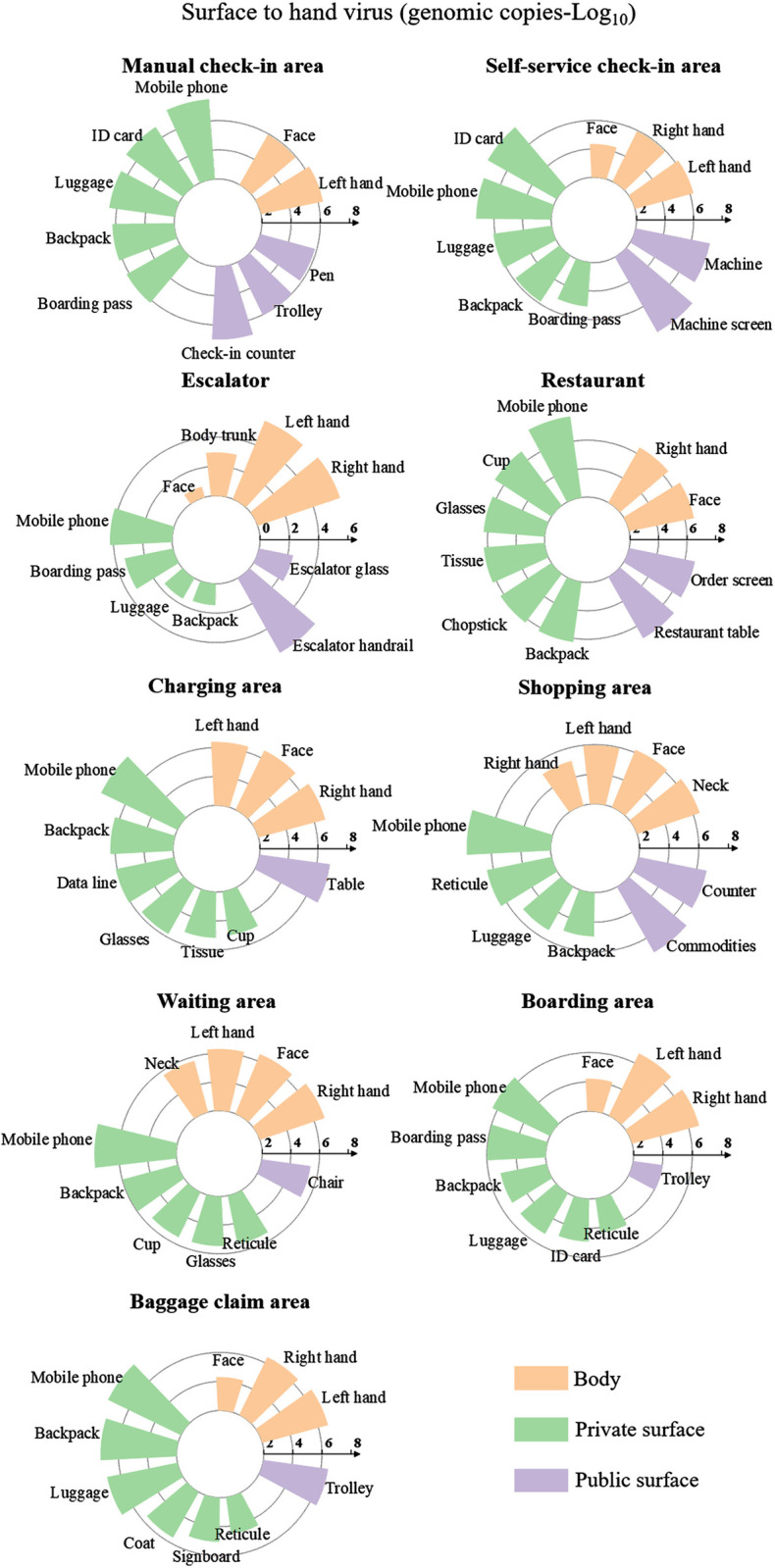
Virus load transferred from surfaces to hands (only top ten surfaces with the highest viral load transfers were listed).

In airports, the highest total amount of virus transferred to hands originates from all private surfaces (4.6×10^7^; 95% CI, 7.4×10^5^−7.4×10^7^ genomic copies), which is 2.2 times greater than the virus transferred from public surfaces to hands (2.1×10^7^; 95% CI, 8.5×10^4^−1.1×10^8^ genomic copies) and 6.6 times greater than the virus transferred from body surfaces to hands (7.0×10^6^; 95% CI, 1.6×10^6^−1.1×10^7^ genomic copies) (Fig 1). Among private surfaces, the highest amount of virus transferred to hands is found on mobile phone surfaces, with an average of 3.4×10^7^ (95% CI, 4.6×10^5^−6.7×10^7^) genomic copies (Fig 1).

### 3.3. Dose-Response

**Fig 2:**
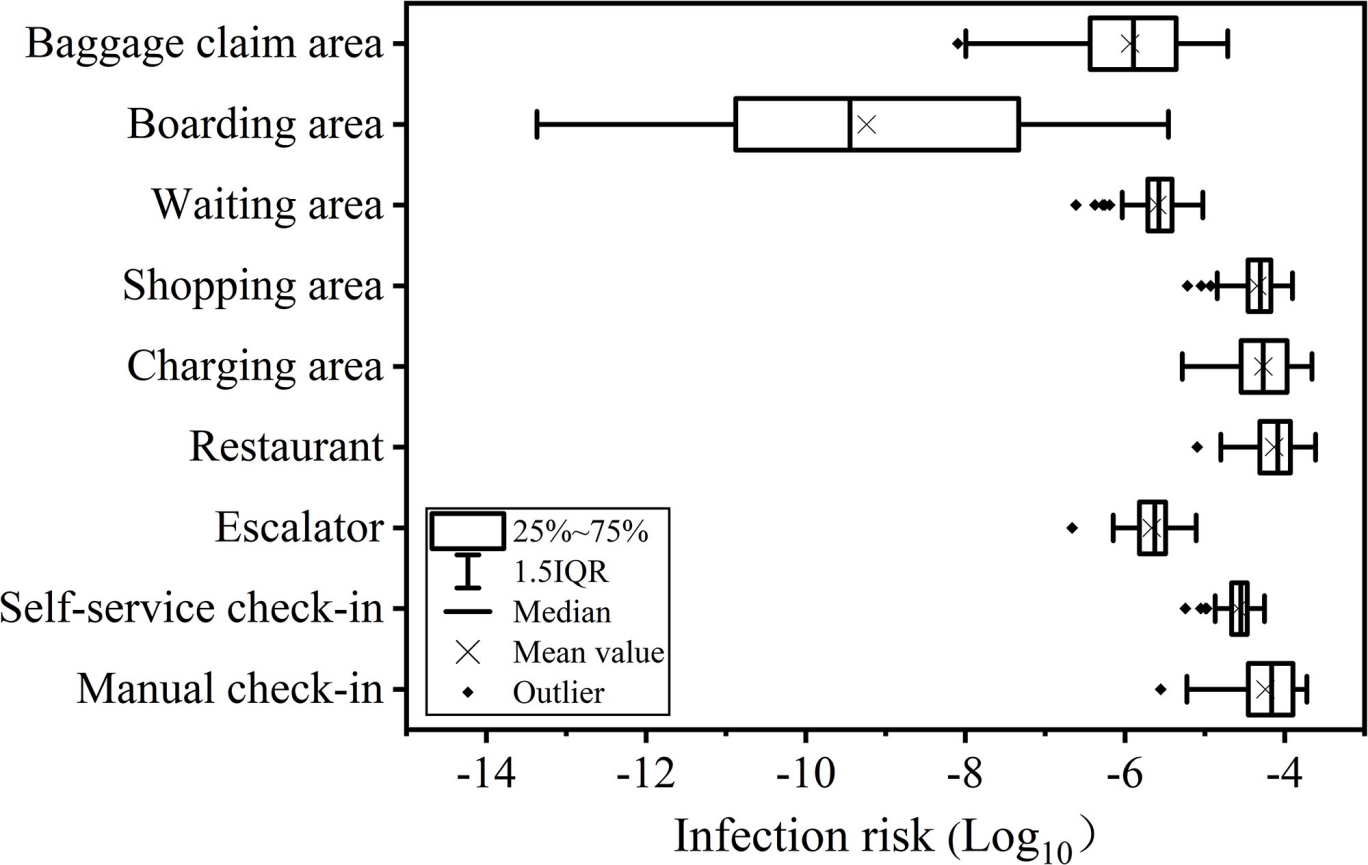
Daily risk of infection in different areas of the airport.

**Fig 3:**
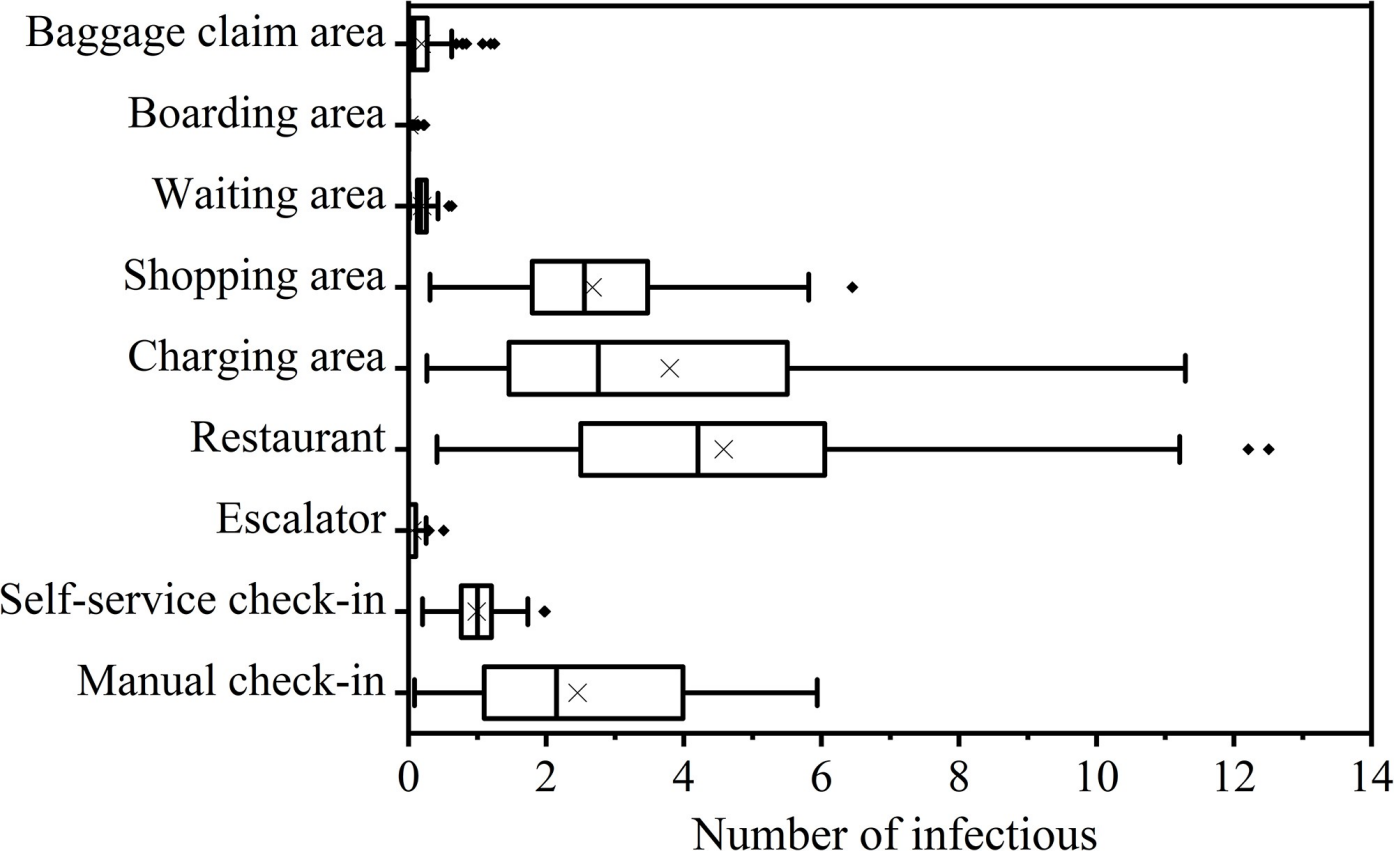
Daily number of infections in different areas of the airport.

Among various airport areas, restaurants exhibit the highest infection risk at 8.8×10^−3^% (95% CI, 1.5×10^−3^%-2.1×10^−2^%), followed by manual check-in areas at 7.8×10^−3^% (0.7×10^−3^%-1.8×10^−2^%) and charging areas at 7.4×10^−3^% (1.1×10^−3^%-2.1×10^−2^%) (Fig 2). Restaurants also account for the highest number of infections, with 4.6 (95% CI, 0.8–10.9) infections expected among 51,494 passengers, followed by charging areas with 3.8 infections (95% CI, 0.6–11.1) among 51,494 passengers (Fig 3).

### 3.4. Intervention measures

#### 3.4.1. Handwashing and public surface disinfection

**Fig 4:**
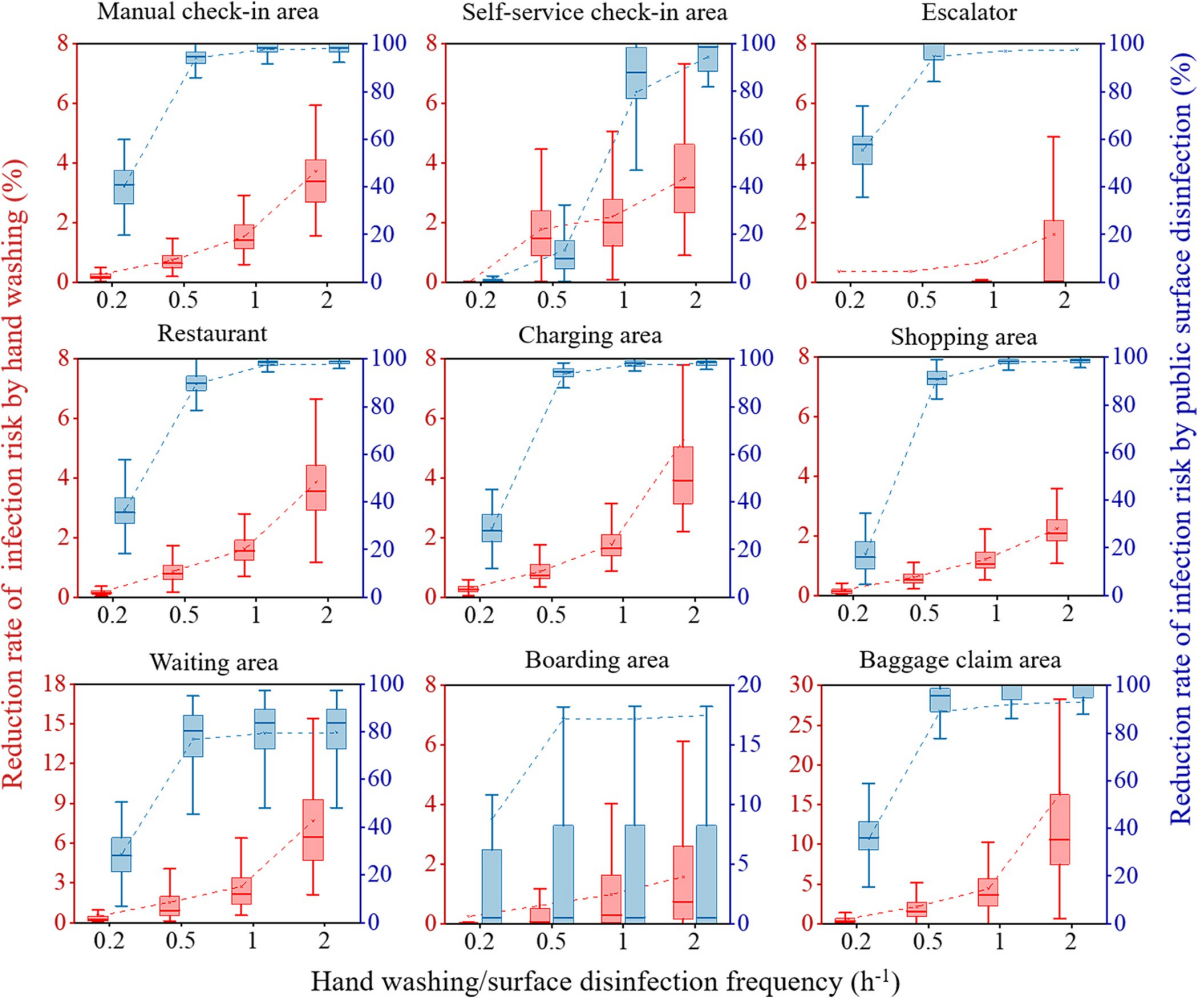
Reduction rate of infection risk by hand washing and public surface disinfection.

Handwashing and public surface disinfection can reduce the infection risk of passengers via fomite route. The model demonstrates that in airports, public surface disinfection is much more effective than hand washing. When passengers washed their hands every two hours, infection risk could be reduced by 2.0%. If all public surfaces can be disinfected with the same frequency, the infection risk could be reduced by 83.2%. As the frequency on public surface disinfection continues to increase, the reduction in infection risk is not significantly different (p = 0.619) (Fig 4).

#### 3.4.2. Wearing masks

**Fig 5:**
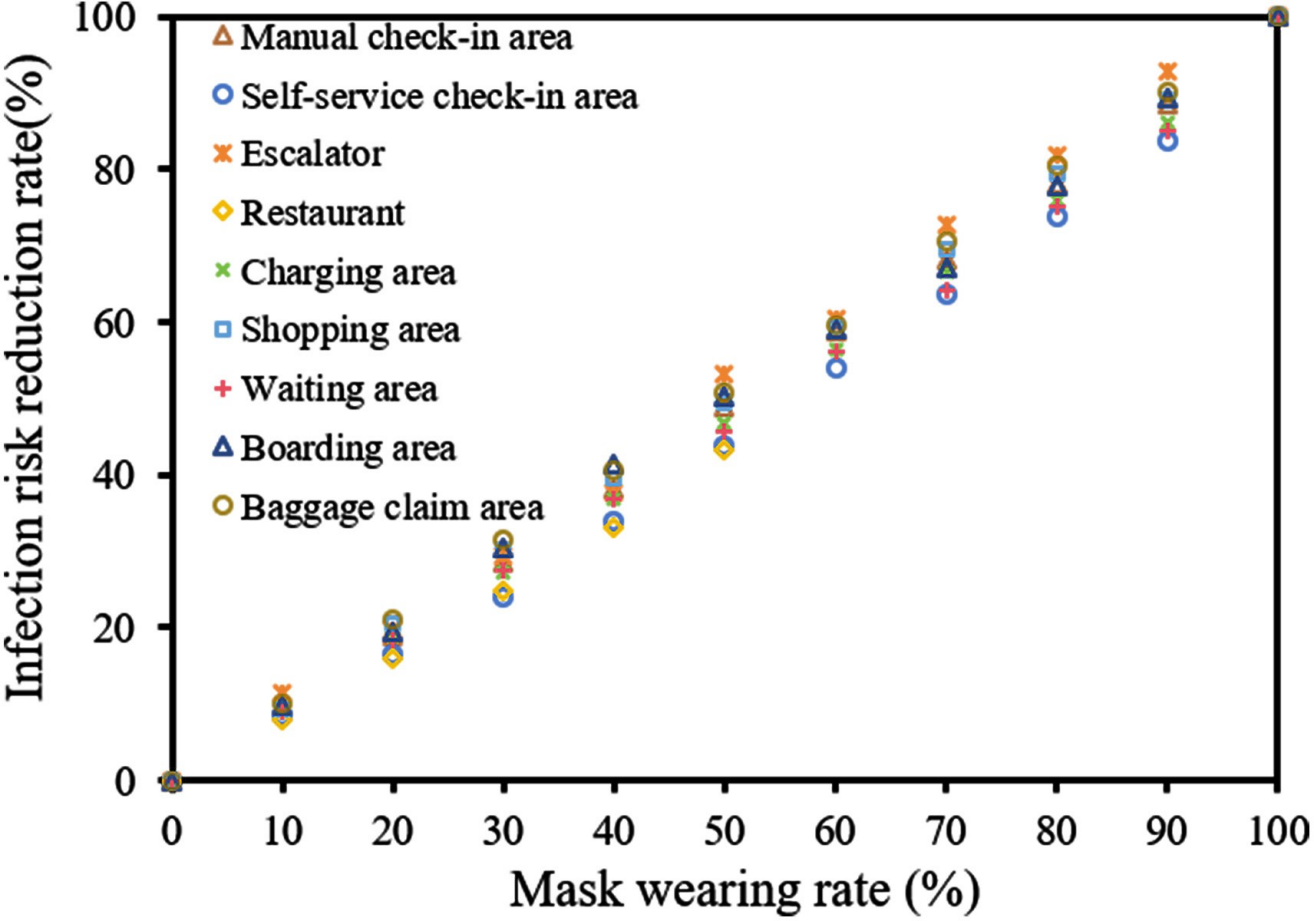
Reduction rate of infection risk caused by passengers wearing masks.

The mask-wearing rate among diners was only 2.5% during the early stage of the COVID-19 pandemic [50]. However, considering diners spent a lot of time on chatting after having the meal, we assumed a maximum mask-wearing rate of 50% for restaurant passengers [29]. This means that all passengers were assumed to wear masks for 50% of the time they spent in the restaurant. In this situation, the overall infection risk would decrease by 48.0% (95% CI, 43.5–52.3%) (Fig 5).

#### 3.4.3. Antibacterial surface

**Fig 6:**
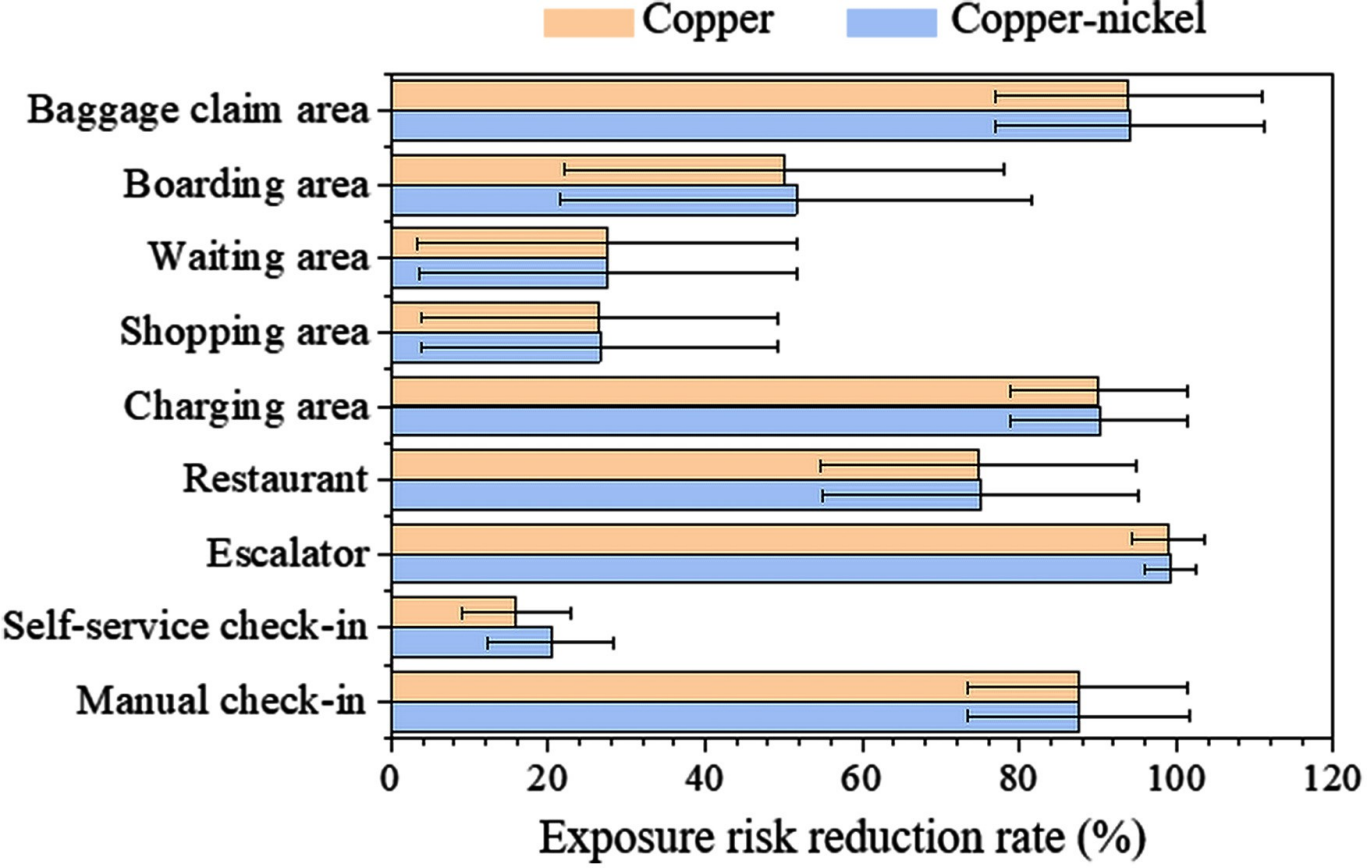
Reduction rate of infection risk caused by installing antimicrobial public surfaces.

Placing antimicrobial surfaces in public areas of the airport can sharply reduce the infection risk via fomite route. However, there is no significant difference in reducing passenger infection risk (p = 0.958) between pure copper or copper-nickel alloys. When these antimicrobial surfaces were installed in escalators, baggage claim areas, and charging areas, the infection risk could be reduced by 98.9–99.2%, 93.9–94.0%, and 90.1–90.2%, respectively. If setting antimicrobial public surfaces in self-service check-in areas, the effectiveness for infection risk reduction is modest, and the infection risk of passengers via surface transmission route only could be reduced by 15.9%-20.4%. This is mainly because in self-service check-in areas, the most frequently touched public surface by passengers is the computer screen (328.5 touches per hour), but screens are difficult to be made by antimicrobial materials. Installing copper/copper-nickel alloy antimicrobial surfaces in different airport areas can on average reduce the infection risk by 59.7/60.5% (Fig 6).

## 4. Discussion

Due to crowded passengers from different cities and countries and a large number of public surfaces, airports have a high potential risk for norovirus transmission via fomite route. Previous studies did the simulation on norovirus transmission without considering real human touch behaviors [51]. This study considered the real touch behavior of passengers in different airports’ areas and establishes a norovirus fomite transmission model based on a Markov chain model considering real human touch behaviors. Using a dose-response model, we quantitatively analyze the infection risk in nine airport areas and assess the effectiveness of interventions such as surface disinfection, mask-wearing, and antimicrobial surfaces.

Among 9 airport areas, due to frequent touches on public surfaces and facial mucous, the restaurant is identified as the highest-risk area for norovirus infection, with an average infection risk of 8.8×10^−3^%. On November 18–19, 2003, a Japanese restaurant served 1,492 guests over two consecutive days, leading to 660 cases of norovirus infection, resulting in a high infection rate of 44% [52]. In October 2017, an Italian self-service restaurant experienced two outbreaks of gastroenteritis within a week, with 13 out of 49 gastroenteritis patients being infected with norovirus [53]. The sources of norovirus outbreaks in restaurants were often human or food, but the transmission routes were diverse. Besides surface-mediated transmission, waterborne and foodborne infections pose significant threats. [54,55]. Furthermore, restaurants are not only high-risk environments for the transmission of Norovirus but also serve as venues for the spread of various other viruses, including SARS-CoV-2 [56].

Handwashing and surface disinfection are common measures for preventing norovirus transmission [51,57]. In airports, we found that public surface disinfection is much more effective than personal handwashing. This difference may stem from comprehensive environmental disinfection, effectively controlling the dispersion of pathogens on surfaces and thereby reducing the risk of infection for subsequent passengers in that area. On the other hand, personal hand hygiene primarily influences an individual’s infection risk, with a relatively smaller impact on subsequent passengers acquiring the virus through contaminated surfaces. Our study found that, performing public surface disinfection every 2 hours is sufficient to effectively reduce the risk of norovirus surface transmission. Moreover, large-scale disinfection measures can be potentially harmful to human health, so targeted disinfection of environmental surfaces is suggested [58,59]. Research indicates that disinfecting frequently touched public surfaces is more effective than disinfecting all surfaces [60]. Our analysis of the touching behavior of passengers in different airport areas has identified frequently touched environmental surfaces, such as check-in counters and luggage trays during check-in and tables and ordering screens in the restaurant area. This discovery can provide guidance to airport personnel for targeted cleaning efforts.

In addition to surface disinfection, the use of antimicrobial surfaces, such as copper and copper alloys, which have been found to expedite the deactivation of certain bacteria, fungi, and viruses [61]. Our study reveals that the installation of copper or copper-nickel antimicrobial surfaces in various areas can reduce the risk of infection transmission through surfaces by an average of 59.7% and 60.5%, respectively. Copper not only exhibits inhibitory effects against norovirus but also accelerates the inactivation of other viruses, such as SARS-CoV-2. While SARS-CoV-2 can survive for up to 48 hours on stainless steel and 72 hours on plastic surfaces [62], it becomes completely inactivated on copper surfaces after 4 hours [62,63]. In healthcare settings, healthcare-associated infections (HAI) are common occurrences, with patients potentially acquiring infections through contact with contaminated surfaces, medical equipment, or tools [64]. Applying antimicrobial coatings to armrests of chairs in intensive care units (ICU) can reduce the pathogen load by as much as 60% [65]. Therefore, actively exploring and implementing antimicrobial surfaces on high-risk surfaces helps curb the spread of infectious diseases. In addition to copper and its alloys being used to prevent microbial transmission, substances like TiO_2_ and silver nanoparticles have also been proven to have inhibitory effects on norovirus and other microorganisms [66,67].

Wearing masks is a common and effective measure to prevent the spread of respiratory diseases, and during the COVID-19 pandemic, governments worldwide have encouraged mask-wearing among the general population [68]. Mask-wearing not only reduces the risk of respiratory diseases being transmitted through airborne and droplets routes but also lowers the frequency of facial mucous touching [33,69,70]. Our study found that if passengers wear masks for 50% of their time at the airport, the risk of infection through the fomite route can be reduced by 48.0%.

There are some limitations of the study. Firstly, since data collection occurred during the COVID-19 pandemic, surface touch behaviors might be changed. We could not obtain the actual touch frequency on all surfaces including facial mucous without mask wearing in airports. While existing literature indicates that mask-wearing does indeed reduce the frequency of mucosal touches, the specific reduction rate varies. Therefore, the actual frequency might differ from our observations under mask-wearing conditions. Secondly, due to the limited data, the input parameters used in the surface transmission model, such as the transfer rate between hands and surfaces (include mucous membranes), virus inactivation rate on surfaces with different materials, surface area, etc., are all point values. When evaluating handwashing and surface disinfection, we assumed an efficiency of 99.99%. This assumption might lead to an overestimation of the effectiveness of abovementioned interventions. Thirdly, the transmission model in this study does not consider the deposition of viruses from droplets or aerosols onto surfaces.

## 5. Conclusions

Passengers in restaurant areas has the highest infection risk of 8.8×10^−3^%, resulting in an average of 4.6 infected individuals out of 51,494. This implies that one passenger with norovirus can potentially infect 4.6 people on average if the infected only toilet once. Regular disinfection of public surfaces, performed every two hours, can reduce the infection risk for passengers by 83.2%, which is much more effective than hand washing. The effectiveness of disinfection every two hours shows no significant difference compared to higher-frequency disinfection. When protective measures such as mask-wearing are adopted, the reduction in the risk of infection for passengers is directly proportional to the mask-wearing rate. Another effective measure to reduce passenger infection risk is the installation of antimicrobial surfaces made of copper or copper-nickel alloy in various areas of the airport, which can reduce the infection risk by an average of 15.9%-99.2%.

## Supporting information

S1 Table Passenger behavior data. (DOCX)

S2 Table Passenger touch event. (DOCX)

S3 Table Surface touch data volume. (DOCX)

S4 Table Surface information: Introduce the surface area, transfer rate from surface to hands/surface to surface, surface material, and virus inactivation rate of the surface. (DOCX)

S5 Table Probability and duration of passengers staying in different areas. (DOCX)

S6 Table Antibacterial surface. (DOCX)

S1 Text Questionnaire on epidemic prevention and control behavior of airport personnel. (DOCX)

S1 Data All data used in the manuscript for tables and figures. (XLSX)
