# Supplementary material for: Public surface disinfection every 2 hours can reduce the infection risk of norovirus in airports up to 83%
Source: PLoS Comput Biol. 2024 Dec 5;20(12):e1012561. doi: 10.1371/journal.pcbi.1012561 (PMC11620375; doi:10.1371/journal.pcbi.1012561)
Supplement: S1 Table — (DOCX) [file pcbi.1012561.s001.docx]

**Table S1.** Passenger behavior data [1]

| Peo  ID | Time | Left hand | | | | Right hand | | | | gender | Mask |
| --- | --- | --- | --- | --- | --- | --- | --- | --- | --- | --- | --- |
|  |  | surf1 | surf2 | owner1 | owner2 | surf1 | surf2 | owner1 | owner2 |  |  |
| 1 | 0:00:00 | 6 | -1 | 1 | -1 | 11 | -1 | 1 | -1 | M | -1 |
| 1 | 0:00:01 | 6 | -1 | 1 | -1 | 11 | -1 | 1 | -1 | M | -1 |
| 1 | 0:00:02 | 6 | -1 | 1 | -1 | 11 | -1 | 1 | -1 | M | -1 |
| 2 | 0:00:03 | 102 | -1 | 4 | -1 | -1 | -1 | -1 | -1 | M | 1 |
| 2 | 0:00:04 | 102 | -1 | 4 | -1 | -1 | -1 | -1 | -1 | M | 1 |

(peoID: Each analyzed person has a numerical code;

Time: The video starts at 0:00:00 and analyzes passenger behavior second by second;

Left/Right surf1, Left/Right surf2: Refers to the surface touched, and -1 indicates that the person did not touch the surface;

Left/Right owner1 and Left/Right owner2: indicate who the surface of the touch belongs to, corresponding to Left/Right surf1 and Left/Right surf2. We define four types of belonging surfces:1- surface belongs to passenger who touch the surface, 2-people accompany the passenger who touch the surface, 3-strangers, or 4-public surfaces. For example, if peoID 1 touches the body trunk, if Owner1 is 1, it means passenger 1 touches their own body trunk, and if Owner1 is 2, it means peoID 1 touches the body trunk of the accompanying person;

Gender: indicates gender, W-female, M-male

Mask: indicates whether the passenger is wearing a mask, 1- with a mask, -1- without a mask.)

**Reference**

1. Zhuang L, Ding Y, Zhou L, Liu R, Ding J, Wang R, et al. Fomite Transmission in Airports Based on Real Human Touch Behaviors. Buildings. 2023; 13:2582.
