# Supplementary material for: Public surface disinfection every 2 hours can reduce the infection risk of norovirus in airports up to 83%
Source: PLoS Comput Biol. 2024 Dec 5;20(12):e1012561. doi: 10.1371/journal.pcbi.1012561 (PMC11620375; doi:10.1371/journal.pcbi.1012561)
Supplement: S2 Table — (DOCX) [file pcbi.1012561.s002.docx]

**Table S2.** Passenger touch event [1].

| peoID | Time | Duration(s) | Surf1 | Surf2 | Owner1 | Owner2 | Hand | Gender |
| --- | --- | --- | --- | --- | --- | --- | --- | --- |
| 1 | 0 | 3 | 6 | -1 | 1 | -1 | 0 | 1 |
| 1 | 0 | 3 | 11 | -1 | 1 | -1 | 1 | 1 |
| 2 | 3 | 40 | 102 | -1 | 4 | -1 | 0 | 1 |
| 2 | 43 | 14 | 65 | -1 | 1 | -1 | 0 | 1 |
| 3 | 28 | 1 | 18 | -1 | 1 | -1 | 0 | 0 |

peoID: Each analyzed person has a numerical code;

Time: represents the start time of a touch. For example, if the video being analyzed is 1 hour long, then 0-3599 is the analysis time length, 0 represents the start time of the touch at 0 seconds, and 43 represents the start time of the touch at 43 seconds;

Duration: represents the duration of a touch, where 3 represents the duration of the touch for 3 seconds;

Surf1, Surf2: Refers to the surface touched, and -1 indicates that the person did not touch the surface;

Owner1 and Owner2: indicate who the surface of the touch belongs to, corresponding to Surf1 and Surf2.

Hand: Indicates which hand is touching the surface, 0- left hand, 1- right hand;

Gender: indicates gender, 0-female, 1-male.

**Reference**

1. Zhuang L, Ding Y, Zhou L, Liu R, Ding J, Wang R, et al. Fomite Transmission in Airports Based on Real Human Touch Behaviors. Buildings. 2023; 13:2582.
