# Supplementary material for: Public surface disinfection every 2 hours can reduce the infection risk of norovirus in airports up to 83%
Source: PLoS Comput Biol. 2024 Dec 5;20(12):e1012561. doi: 10.1371/journal.pcbi.1012561 (PMC11620375; doi:10.1371/journal.pcbi.1012561)
Supplement: S3 Table — (DOCX) [file pcbi.1012561.s003.docx]

**Table S3. Surface touch data volume [1].**

| Airport’s area | Video duration (s) | Number of recorded passengers | Data volume^1^  (person∙second) | Number of touches |
| --- | --- | --- | --- | --- |
| Manual check-in area | 6,349 | 76 | 22,915 | 3,140 |
| Self-service check-in area | 12,973 | 111 | 9,848 | 2,390 |
| Escalator | 12,452 | 1,047 | 29,212 | 4,258 |
| Restaurant | 10,549 | 111 | 59,977 | 7,474 |
| Charging area | 5,573 | 29 | 20,948 | 1,820 |
| Shopping area | 8,294 | 9 | 10,060 | 416 |
| Waiting area | 15,843 | 192 | 68,449 | 3,786 |
| Boarding area | 1,107 | 139 | 11,025 | 2,036 |
| Baggage claim area | 3,600 | 46 | 9,233 | 605 |

**Reference**

1. Zhuang L, Ding Y, Zhou L, Liu R, Ding J, Wang R, et al. Fomite Transmission in Airports Based on Real Human Touch Behaviors. Buildings. 2023; 13:2582.
