# Supplementary material for: Public surface disinfection every 2 hours can reduce the infection risk of norovirus in airports up to 83%
Source: PLoS Comput Biol. 2024 Dec 5;20(12):e1012561. doi: 10.1371/journal.pcbi.1012561 (PMC11620375; doi:10.1371/journal.pcbi.1012561)
Supplement: S4 Table — (DOCX) [file pcbi.1012561.s004.docx]

**Table S4.** Surface information.

| Primary surface | | Sub-surface | Code | Area^1^  (cm²) | Transfer rate^2^ | | Material | Inactivation rate^3^  (min^-1^) | Surface items |
| --- | --- | --- | --- | --- | --- | --- | --- | --- | --- |
|  |  |  |  |  | Hand to  surface | Surface to hand |  |  |  |
| Mucosa  （M） | | Eye | 1 | 3 | 0.34 | 0 | mucous | 0 | 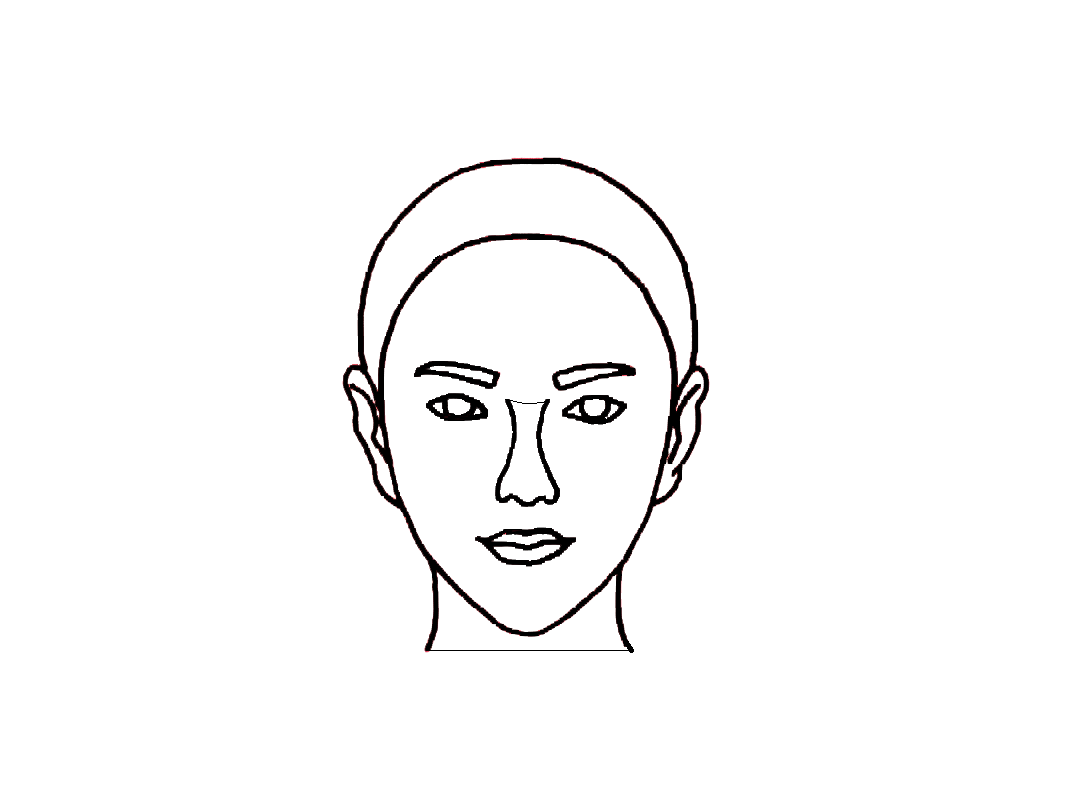 |
|  |  | Mouth | 2 | 6 | 0.34 | 0 | mucous | 0 |  |
|  |  | Nose | 3 | 4 | 0.34 | 0 | mucous | 0 |  |
| Hand  （H） | | Left hand | 4 | 160 | 0.1817 | 0.1817 | hand | 0.04 | 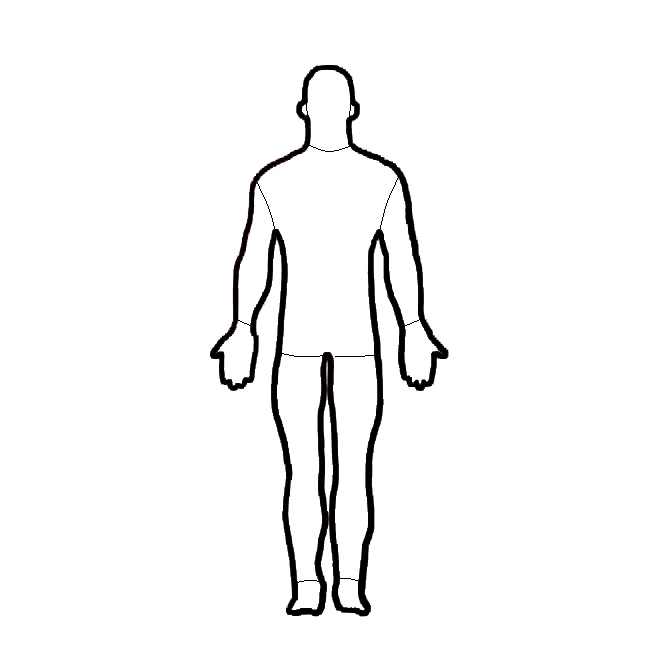 |
|  |  | Right hand | 5 | 160 | 0.1817 | 0.1817 | hand | 0.04 |  |
| Body  （B） | Main body | Body  trunk | 6 | 4,500 | 0.6675 | 0.0149 | fabric/cloth | 0.008 |  |
|  |  | Arm | 7 | 2,500 | 0.6675 | 0.0149 | fabric/cloth | 0.008 |  |
|  |  | Leg | 8 | 4,000 | 0.6675 | 0.0149 | fabric/cloth | 0.008 |  |
|  | Face  neck | Face | 9 | 500 | 0.1817 | 0.1817 | Head | 0.04 |  |
|  |  | Neck | 10 | 2,000 | 0.1817 | 0.1817 | face/neck | 0.04 |  |
| Personal Private Objects  (PR) | | Mobile phone | 11 | 240 | 0.1934 | 0.1803 | Glass | 0.0021 | 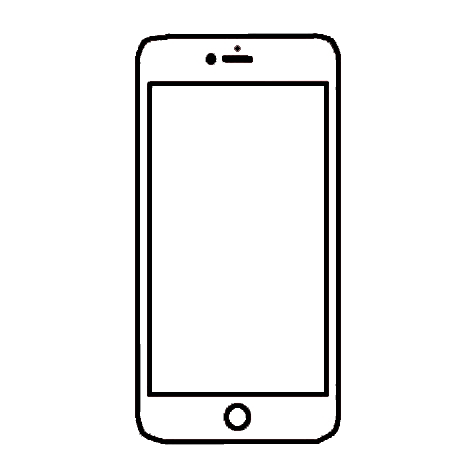 |
|  |  | Mask | 12 | 160 | 0.6675 | 0.0149 | Mask | -1 | 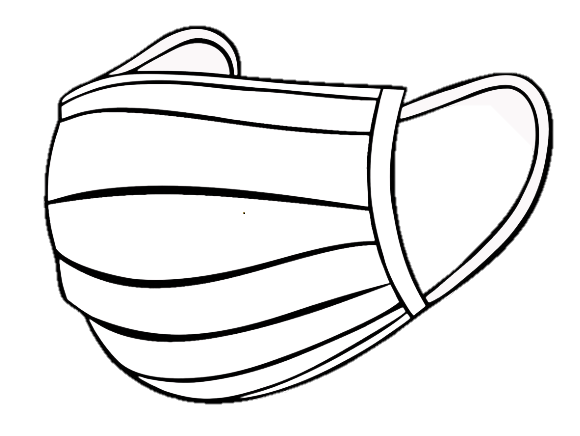 |
|  |  | Pen | 13 | 50 | 0.11 | 0.18 | Pen | 0.0021 | 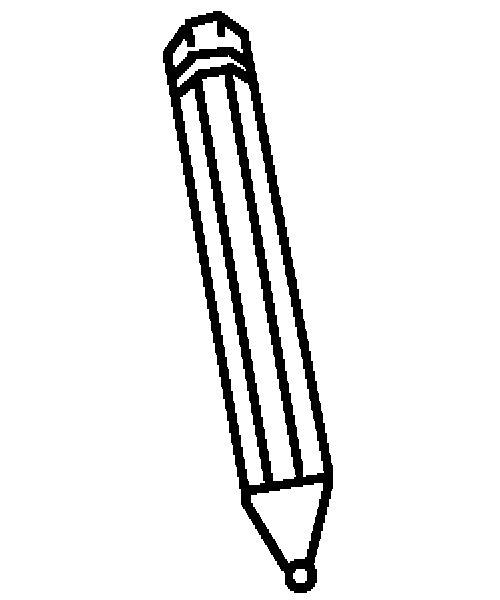 |
|  |  | Glove | 14 | 160 | 0.12 | 0.07 | Non-porous | 0.0021 | 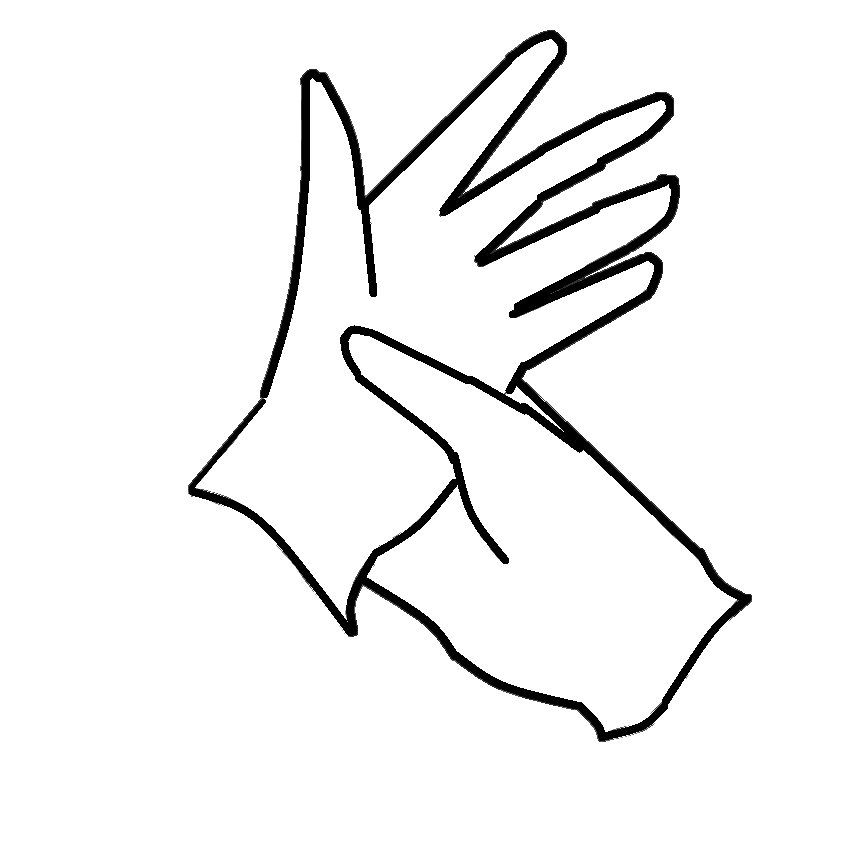 |
|  |  | Trunk box | 15 | 4,500 | 0.12 | 0.07 | Non-porous | 0.0021 | 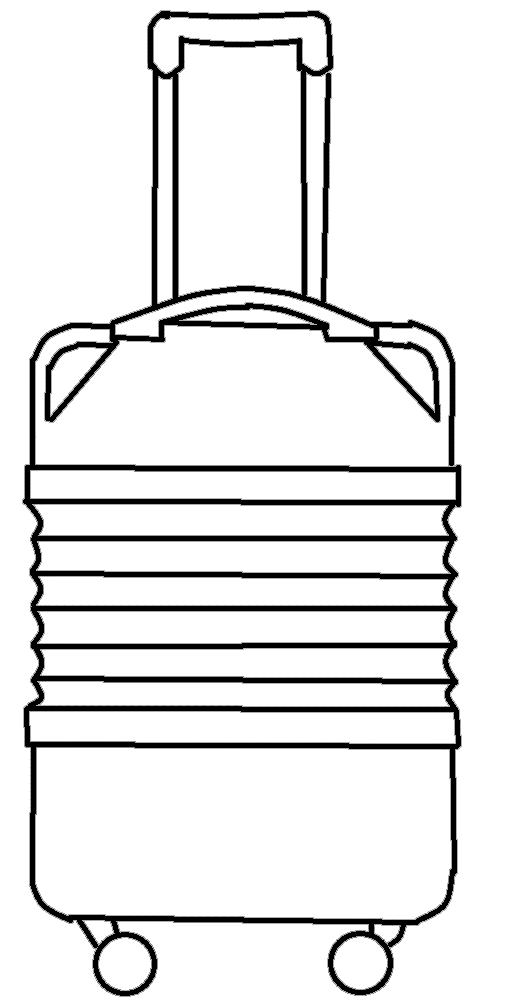 |
|  |  | Luggage compartment handle | 16 | 130 | 0.12 | 0.07 | Non-porous | 0.0021 |  |
|  |  | Backpack strap | 17 | 150 | 0.46 | 0.05 | Porous/non-porous | 0.008 | 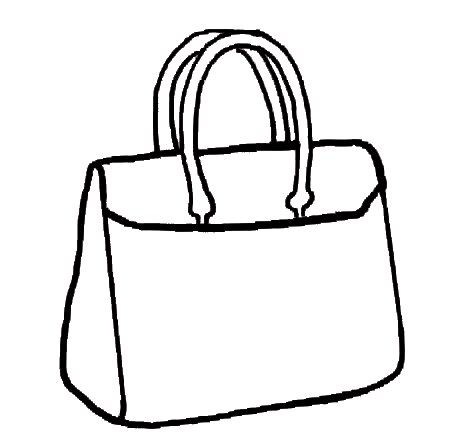 |
|  |  | Backpack body | 18 | 4,000 | 0.46 | 0.05 | Porous/non-porous | 0.008 |  |
|  |  | Cup | 19 | 170 | 0.12 | 0.07 | Non-porous | 0.0021 | 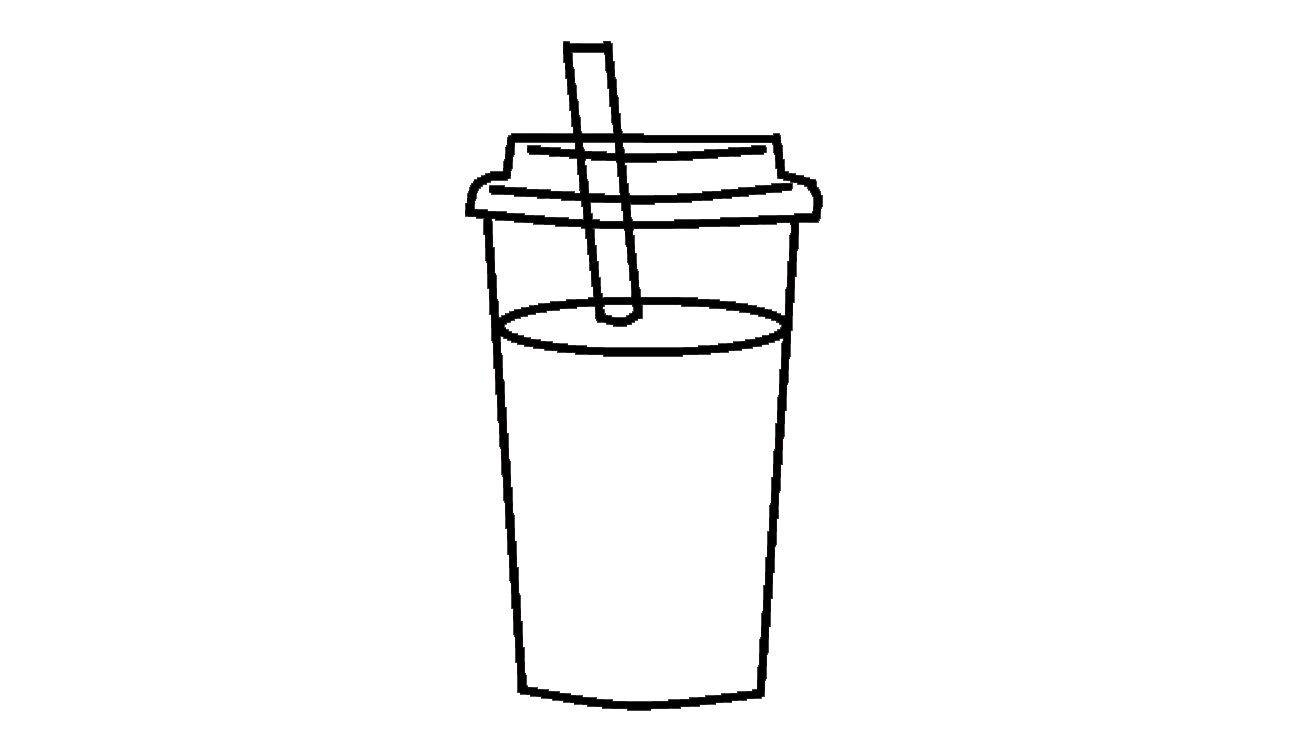 |
|  |  | File pocket | 20 | 1,500 | 0.12 | 0.07 | Non-porous | 0.0021 | 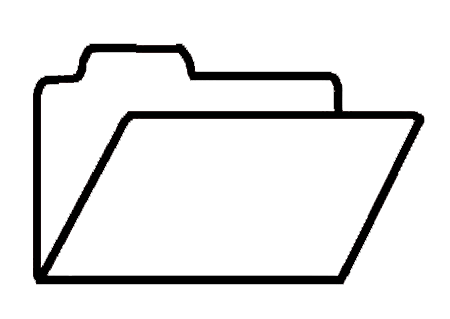 |
|  |  | Camera | 21 | 600 | 0.12 | 0.07 | Non-porous | 0.0021 | 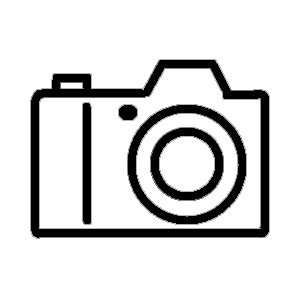 |
|  |  | Computer | 22 | 3,000 | 0.12 | 0.07 | Non-porous | 0.0021 | 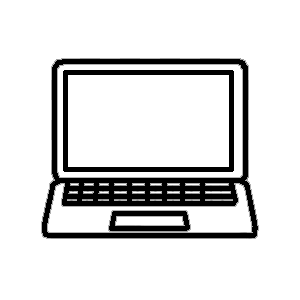 |
|  |  | ID card | 23 | 90 | 0.12 | 0.07 | Non-porous | 0.0021 | 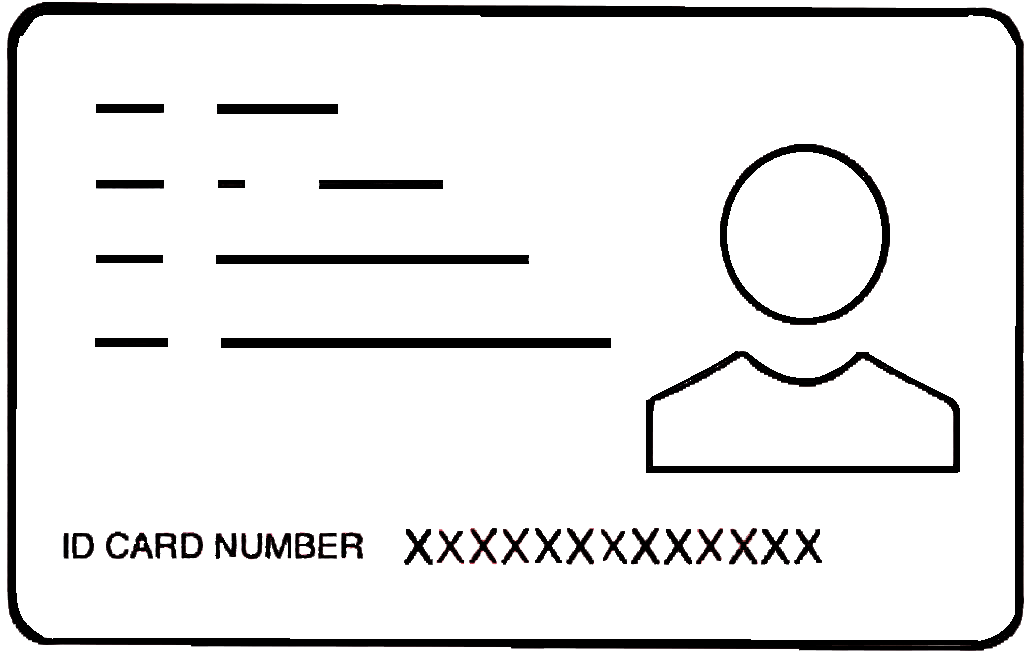 |
|  |  | Household register | 24 | 300 | 0.6675 | 0.0149 | Paper/tissue | 0.008 | 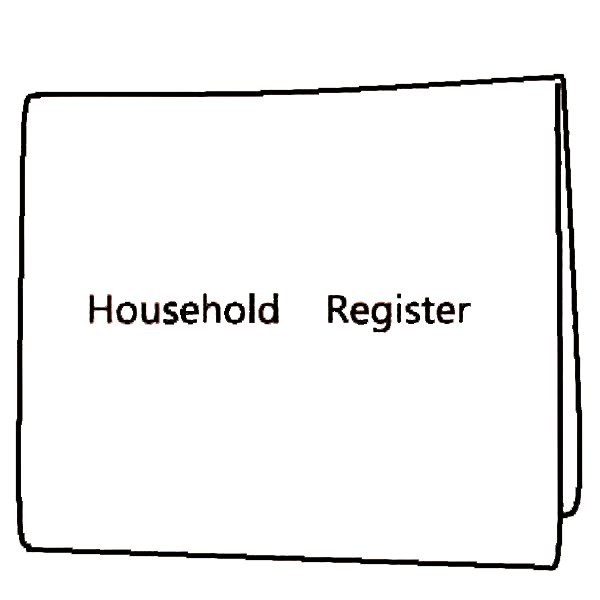 |
|  |  | U-shaped pillow | 25 | 1200 | 0.6675 | 0.0149 | Fabric/cloth | 0.008 | 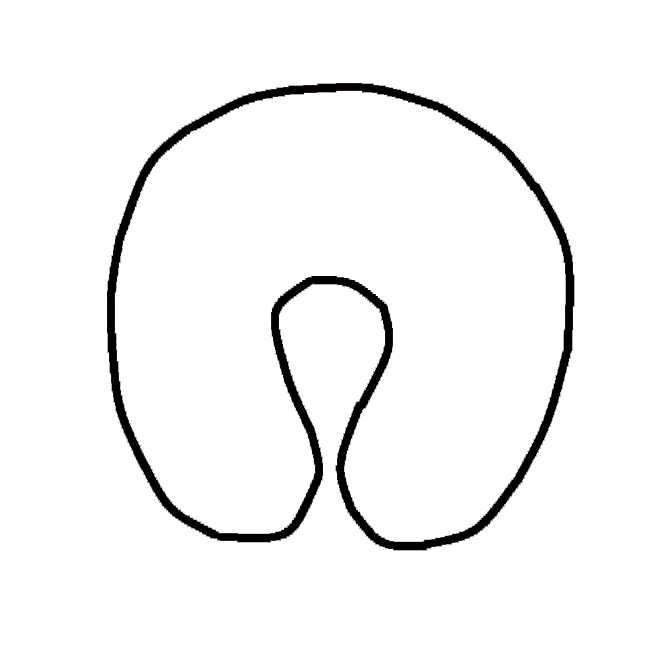 |
|  |  | Package | 26 | 2,000 | 0.6675 | 0.0149 | Paper/tissue | 0.008 | 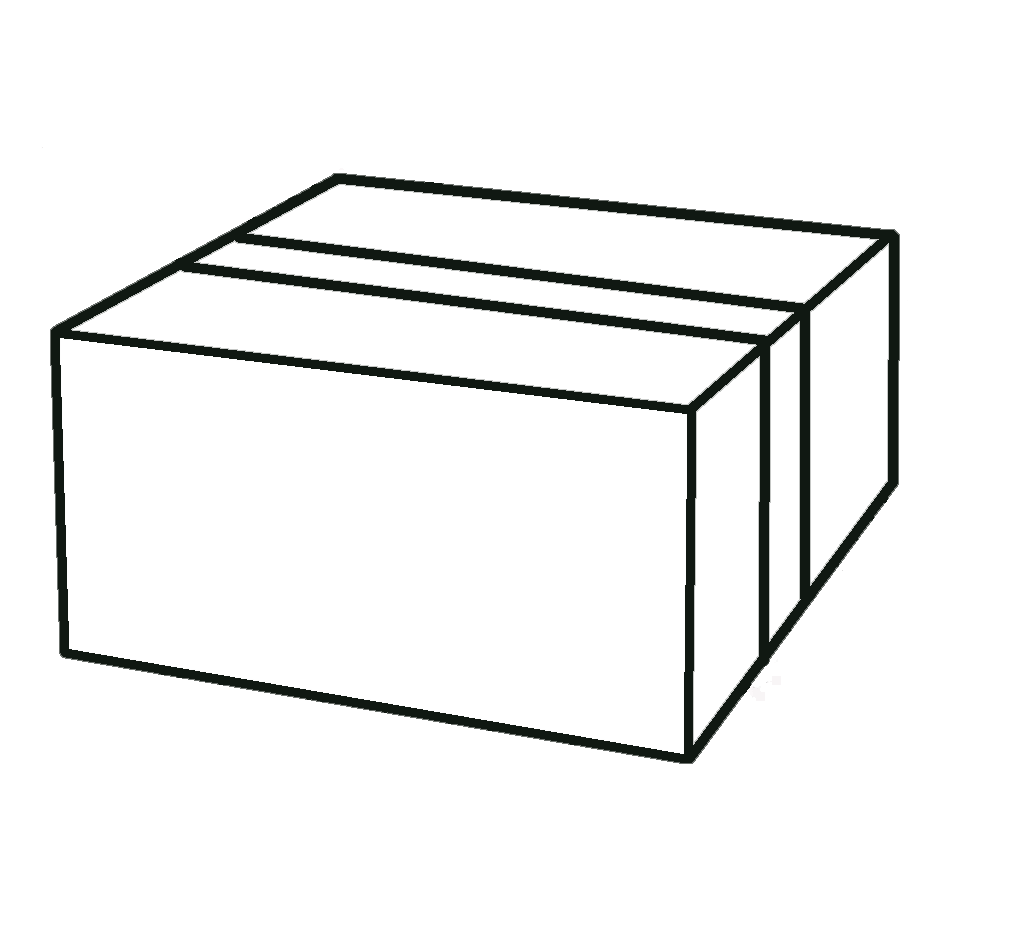 |
|  |  | Umbrella | 27 | 200 | 0.12 | 0.07 | Non-porous | 0.0021 | 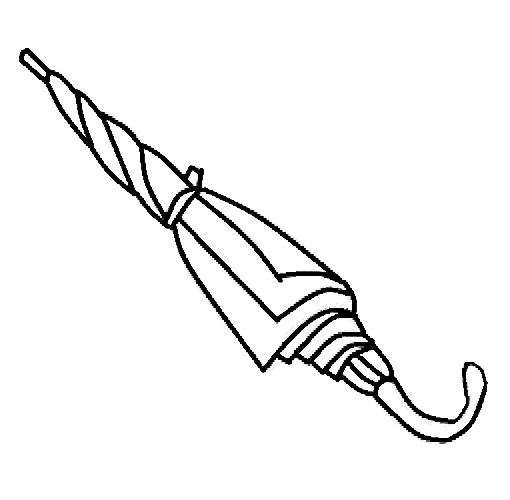 |
|  |  | Key | 28 | 20 | 0.1669 | 0.1152 | Stainless steel | 0.0021 | 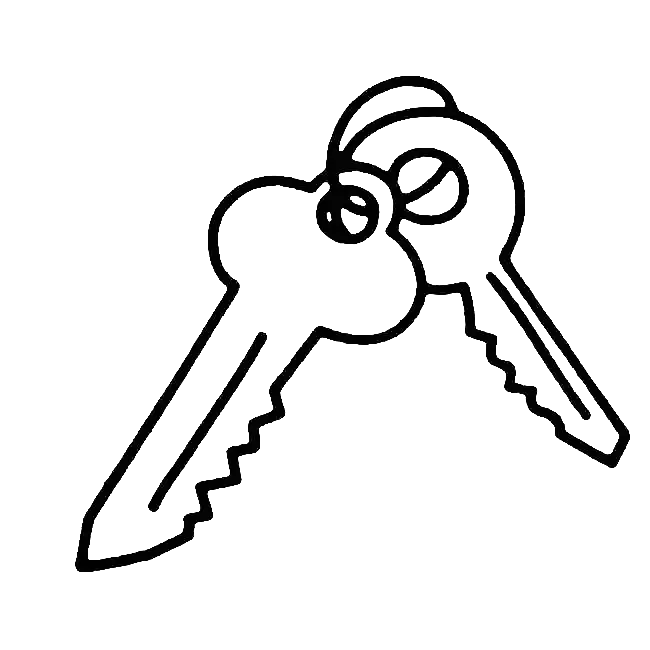 |
|  |  | Tissue | 29 | 400 | 0.6675 | 0.0149 | Paper/tissue | 0.008 | 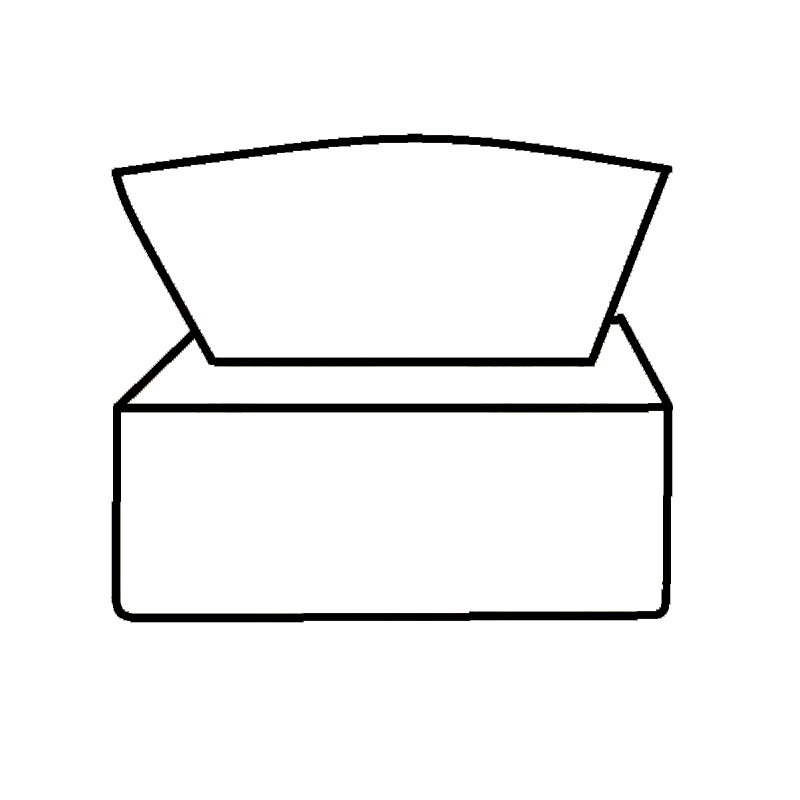 |
|  |  | Doll | 30 | 1,500 | 0.6675 | 0.0149 | fabric/cloth | 0.008 | 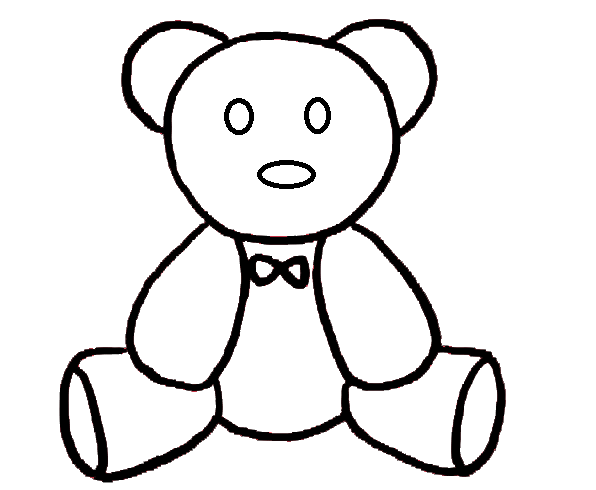 |
|  |  | Walkie-talkie | 31 | 100 | 0.12 | 0.07 | Non-porous | 0.0021 | 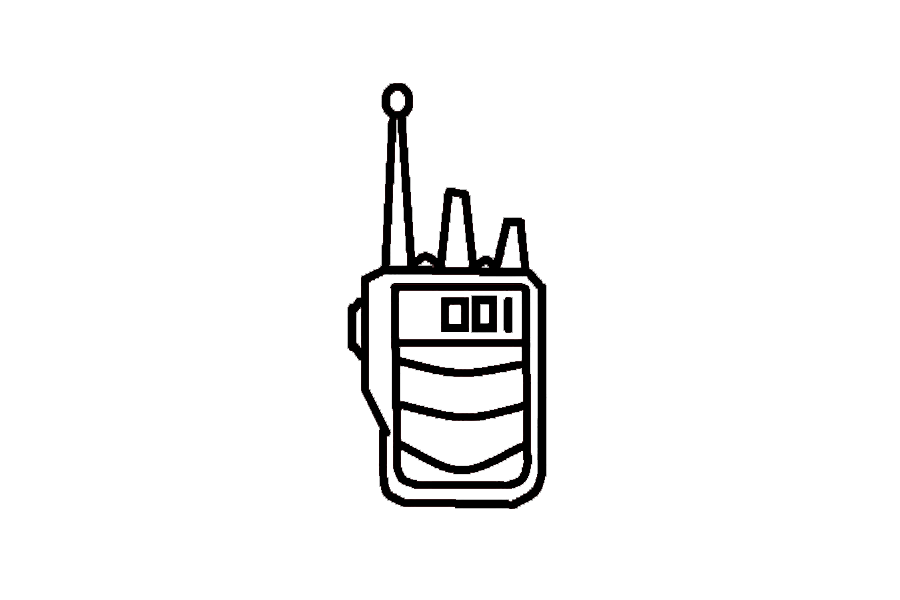 |
|  |  | Traditional Chinese handicraft | 32 | 25 | 0.8 | 0.03 | undefined material | 0.008 | 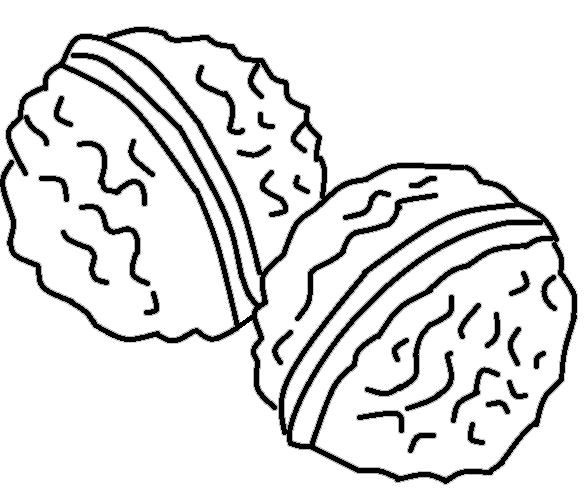 |
|  |  | Tissue bag | 33 | 650 | 0.12 | 0.07 | Non-porous | 0.0021 | 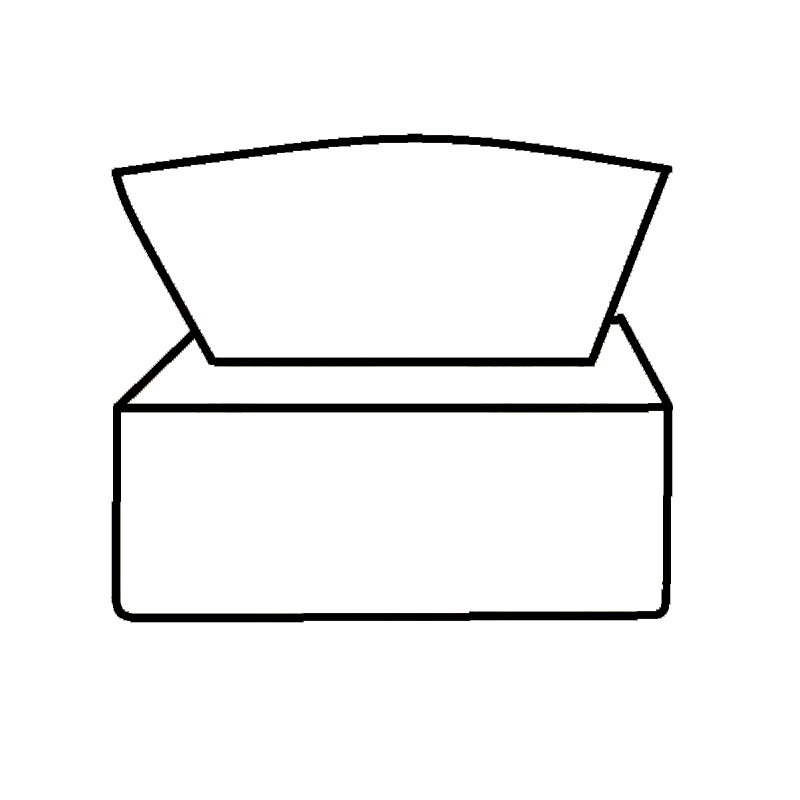 |
|  |  | Coat | 34 | 7,000 | 0.6675 | 0.0149 | fabric/cloth | 0.008 | 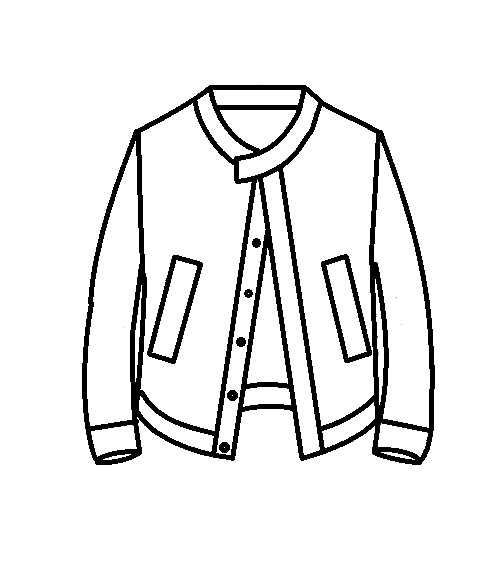 |
|  |  | Glasses | 35 | 100 | 0.1934 | 0.1803 | Glass | 0.0021 | 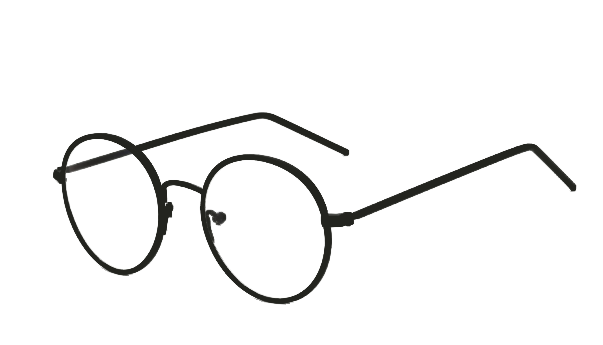 |
|  |  | Headset | 36 | 10 | 0.12 | 0.07 | Non-porous | 0.0021 | 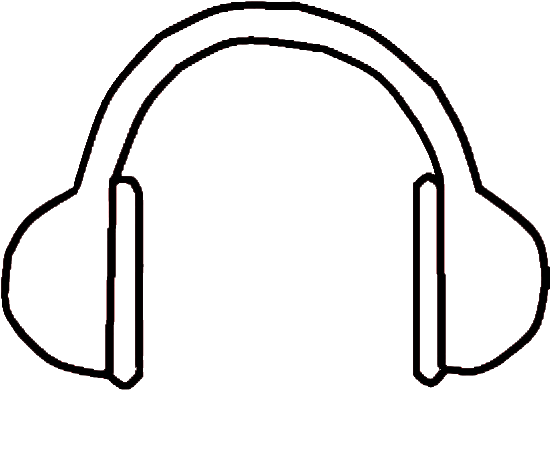 |
|  |  | Reticule | 37 | 1,800 | 0.46 | 0.05 | Porous/non-porous | 0.008 | 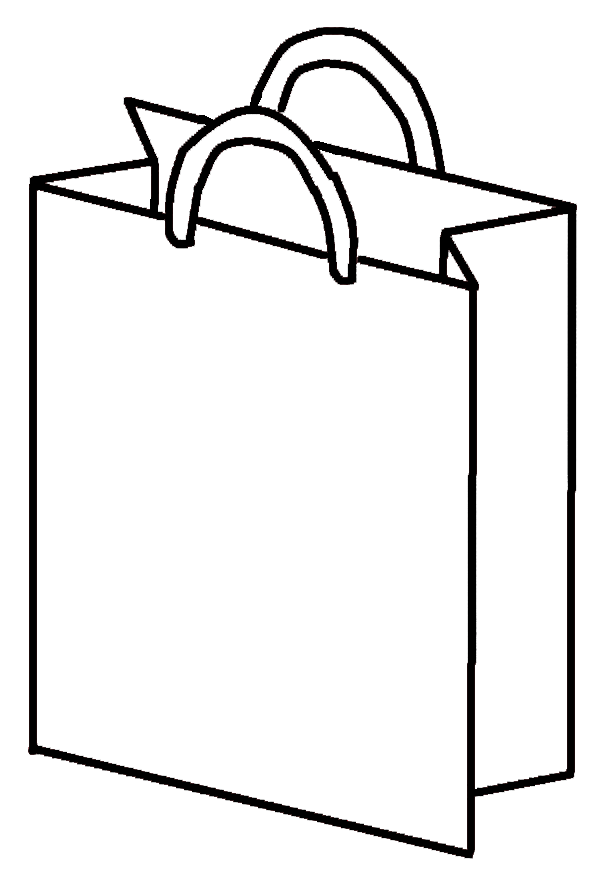 |
|  |  | Wallet | 38 | 600 | 0.12 | 0.07 | Non-porous | 0.0021 | 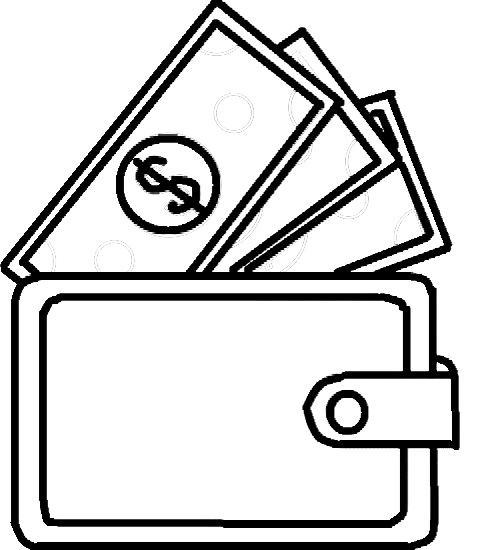 |
|  |  | Data line | 39 | 40 | 0.12 | 0.07 | Non-porous | 0.0021 | 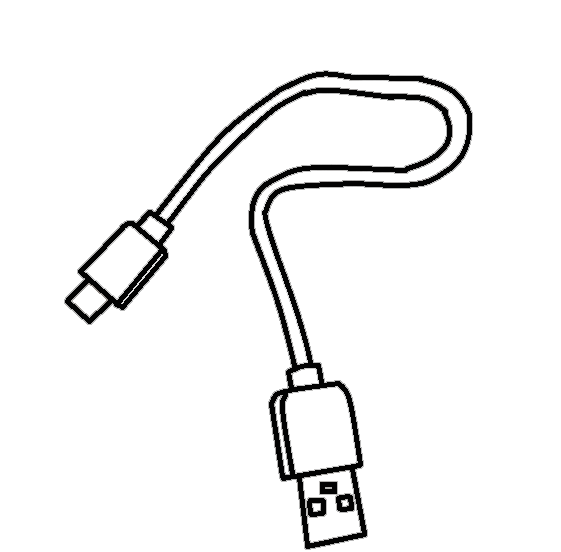 |
|  |  | Baby stroller | 40 | 2,000 | 0.12 | 0.07 | Non-porous | 0.0021 | 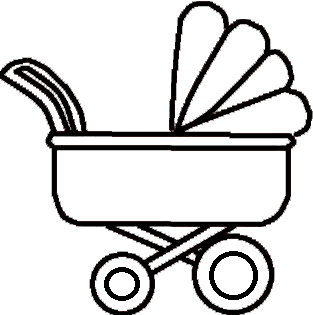 |
|  |  | Hat | 41 | 300 | 0.6675 | 0.0149 | Fabric/cloth | 0.008 | 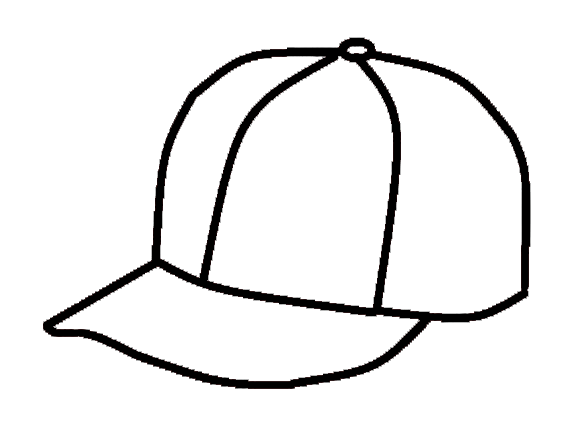 |
|  |  | Shoes | 42 | 600 | 0.12 | 0.07 | Non-porous | 0.0021 | 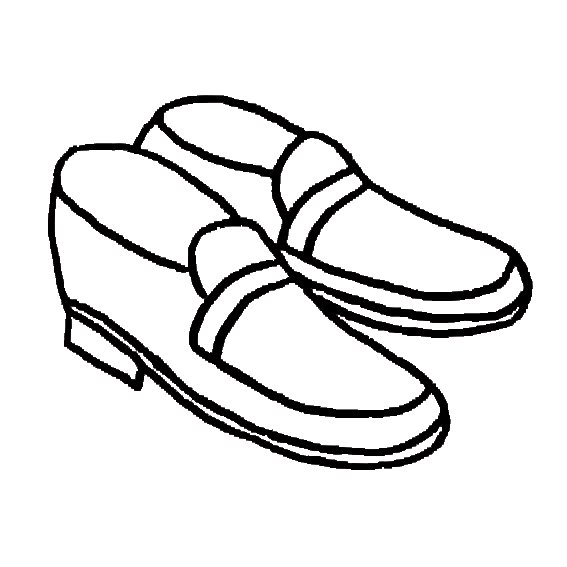 |
|  |  | Private others | 43 | 200 | 0.46 | 0.05 | Porous/non-porous | 0.008 |  |
|  |  | Plastic instant noodles bowl | 44 | 380 | 0.12 | 0.07 | Non-porous | 0.0021 | 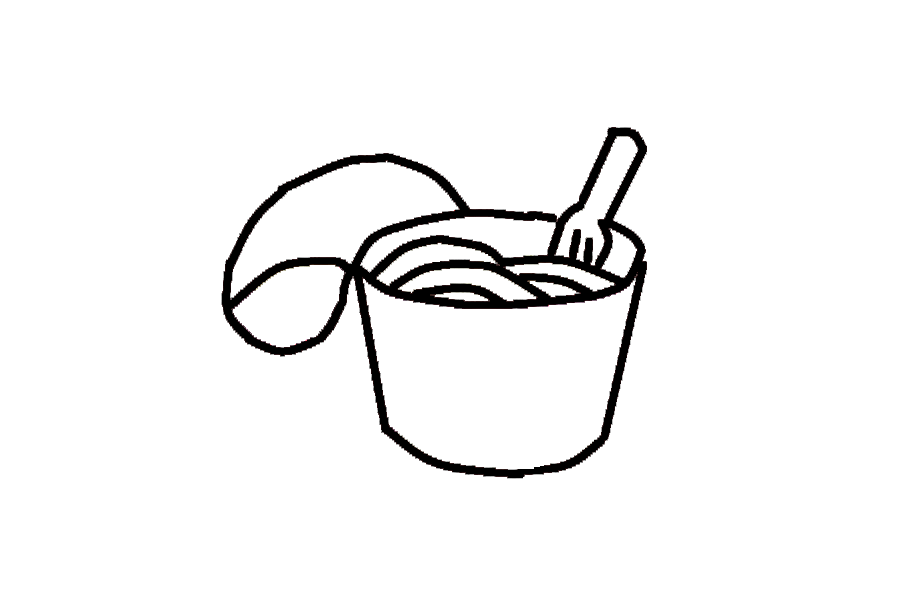 |
|  |  | Bracelet | 45 | 12 | 0.12 | 0.07 | Non-porous | 0.0021 | 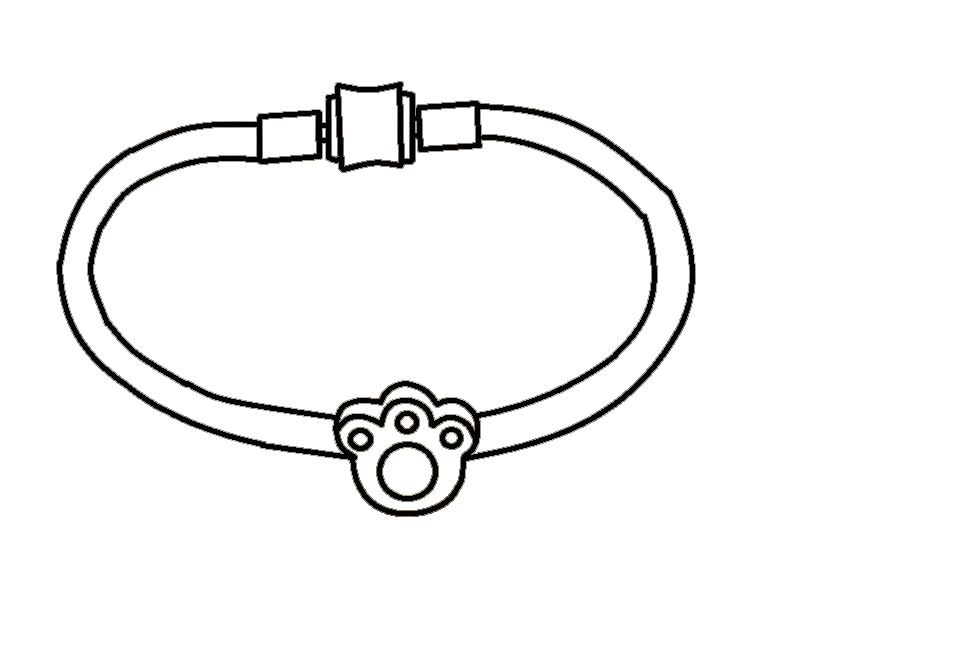 |
|  |  | Paper | 46 | 623 | 0.6675 | 0.0149 | paper | 0.5 | 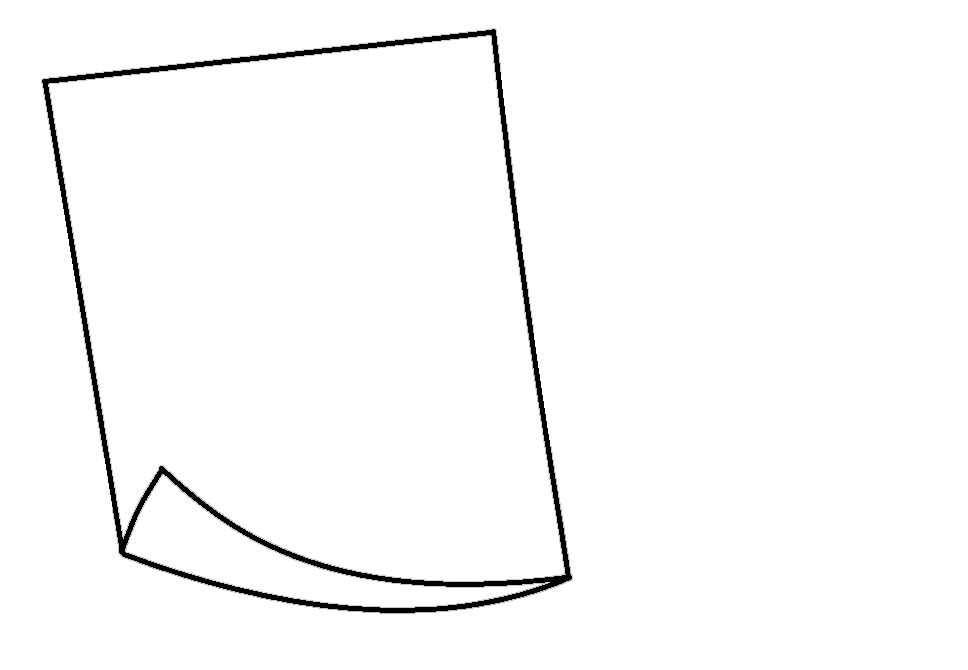 |
|  |  | Headphone box | 47 | 35 | 0.12 | 0.07 | Non-porous | 0.0021 | 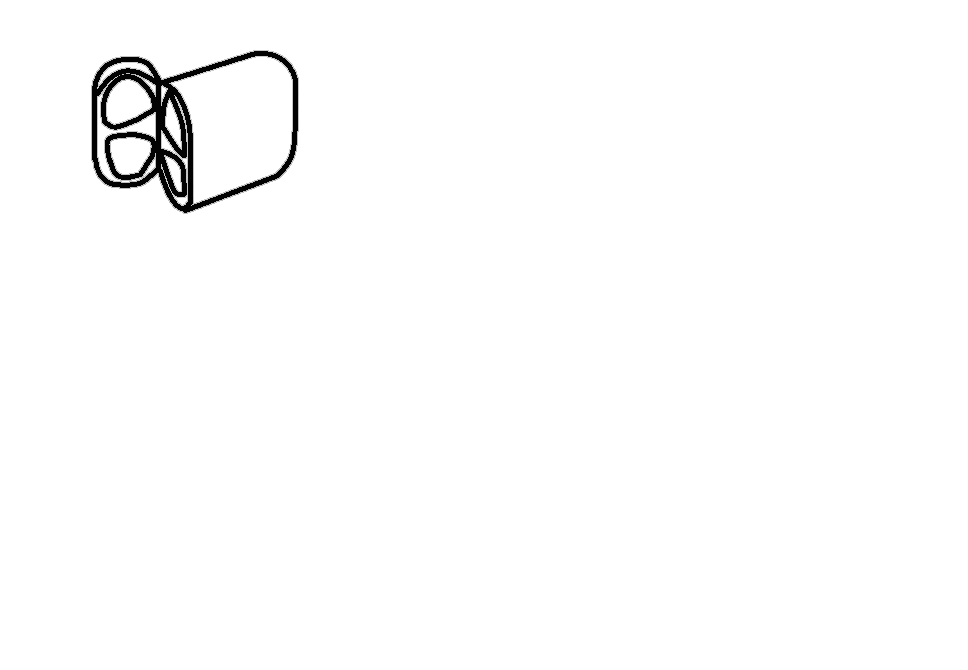 |
|  |  | Wrist watch | 48 | 20 | 0.1934 | 0.1803 | Glass | 0.0021 | 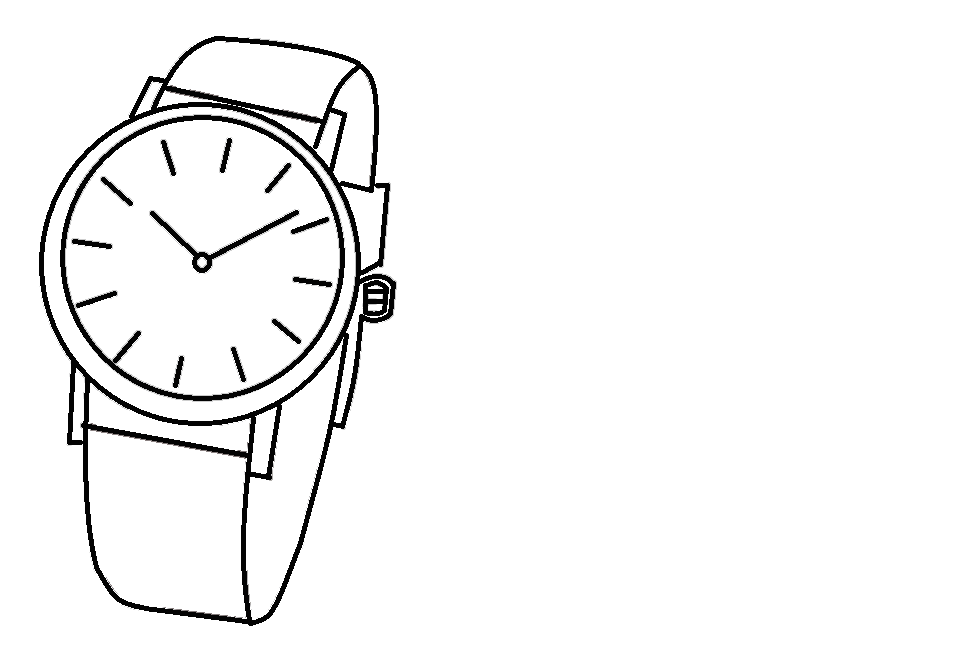 |
|  |  | Book | 49 | 800 | 0.6675 | 0.0149 | Paper/tissue | 0.008 | 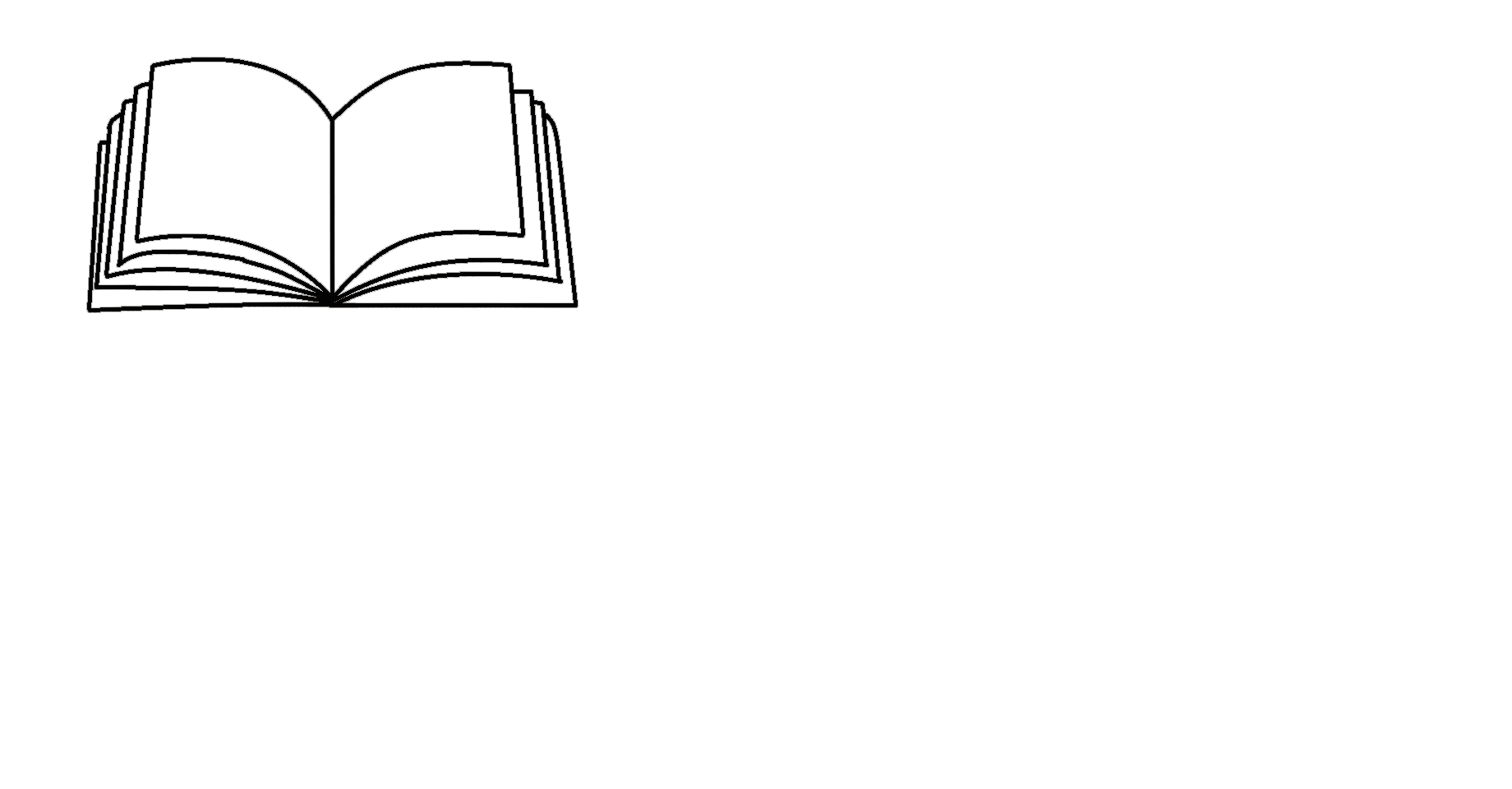 |
|  |  | Armband | 50 | 200 | 0.6675 | 0.0149 | fabric/cloth | 0.008 | 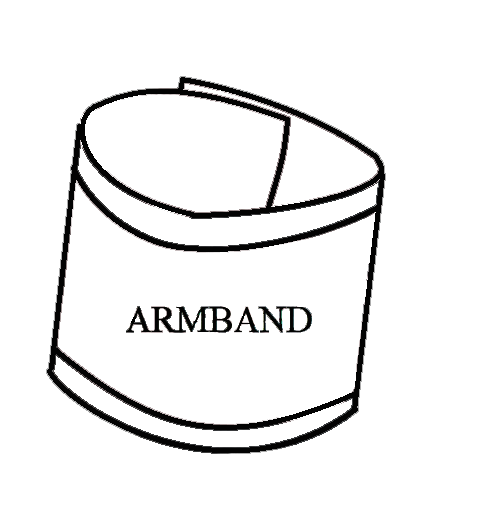 |
|  |  | Waist rope | 51 | 12 | 0.6675 | 0.0149 | porous | 0.008 | 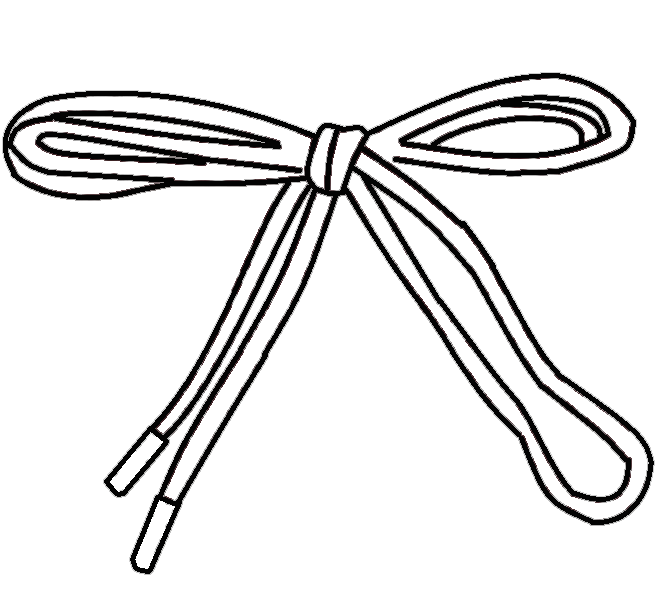 |
|  |  | Portable battery | 52 | 300 | 0.12 | 0.07 | Non-porous | 0.0021 | 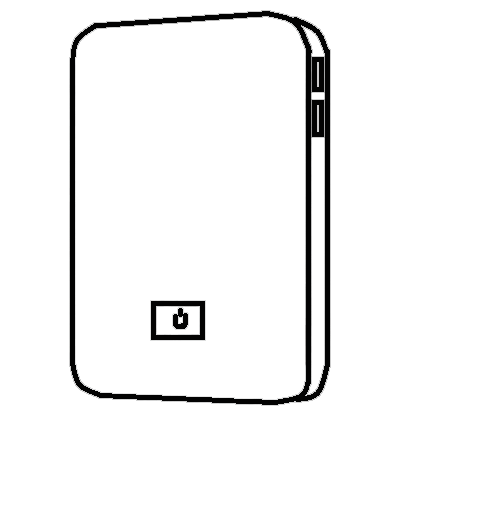 |
|  |  | Medicine kit | 53 | 200 | 0.12 | 0.07 | Non-porous | 0.0021 | 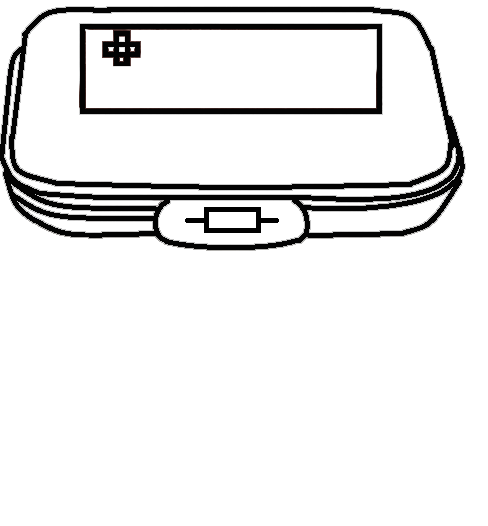 |
|  |  | Drug | 54 | 5 | 0.12 | 0.07 | Non-porous | 0.0021 | 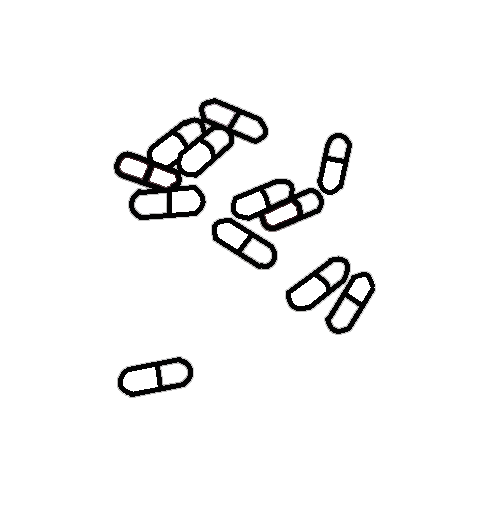 |
|  |  | Mouse | 55 | 120 | 0.12 | 0.07 | Non-porous | 0.0021 | 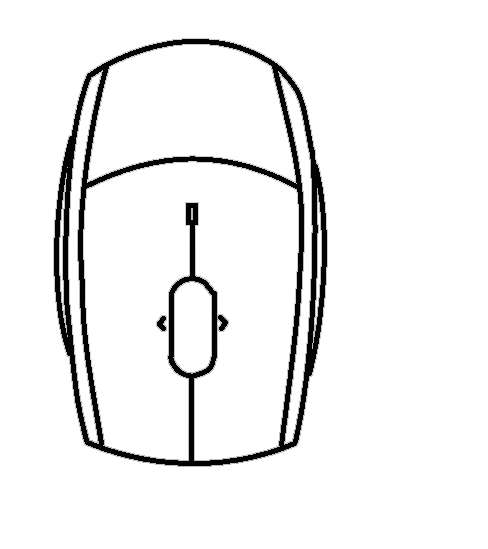 |
|  |  | Fan | 56 | 300 | 0.12 | 0.07 | Non-porous | 0.008 | 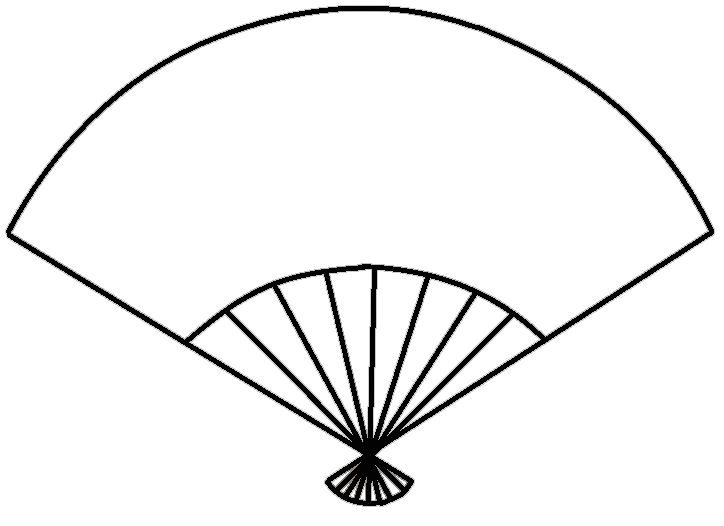 |
|  |  | Plastic fork | 57 | 25 | 0.12 | 0.07 | Non-porous | 0.0021 | 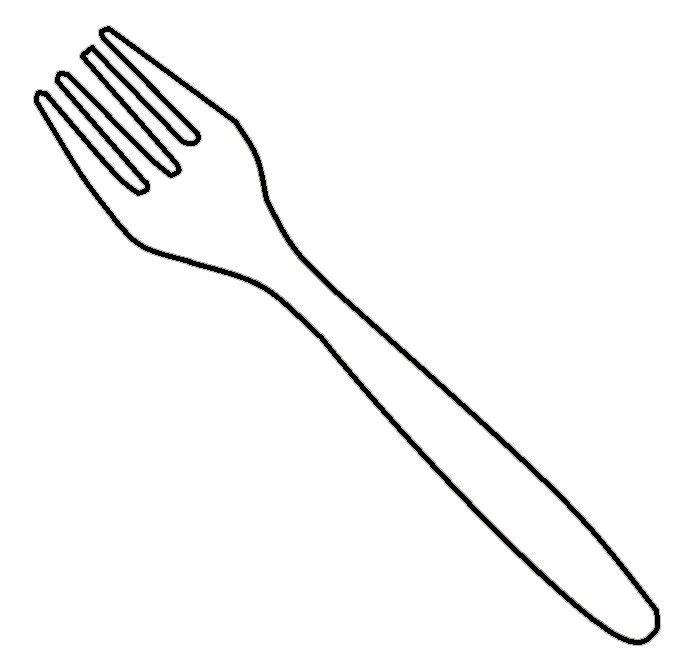 |
|  |  | Food packaging box | 58 | 500 | 0.6675 | 0.0149 | Paper/tissue | 0.008 | 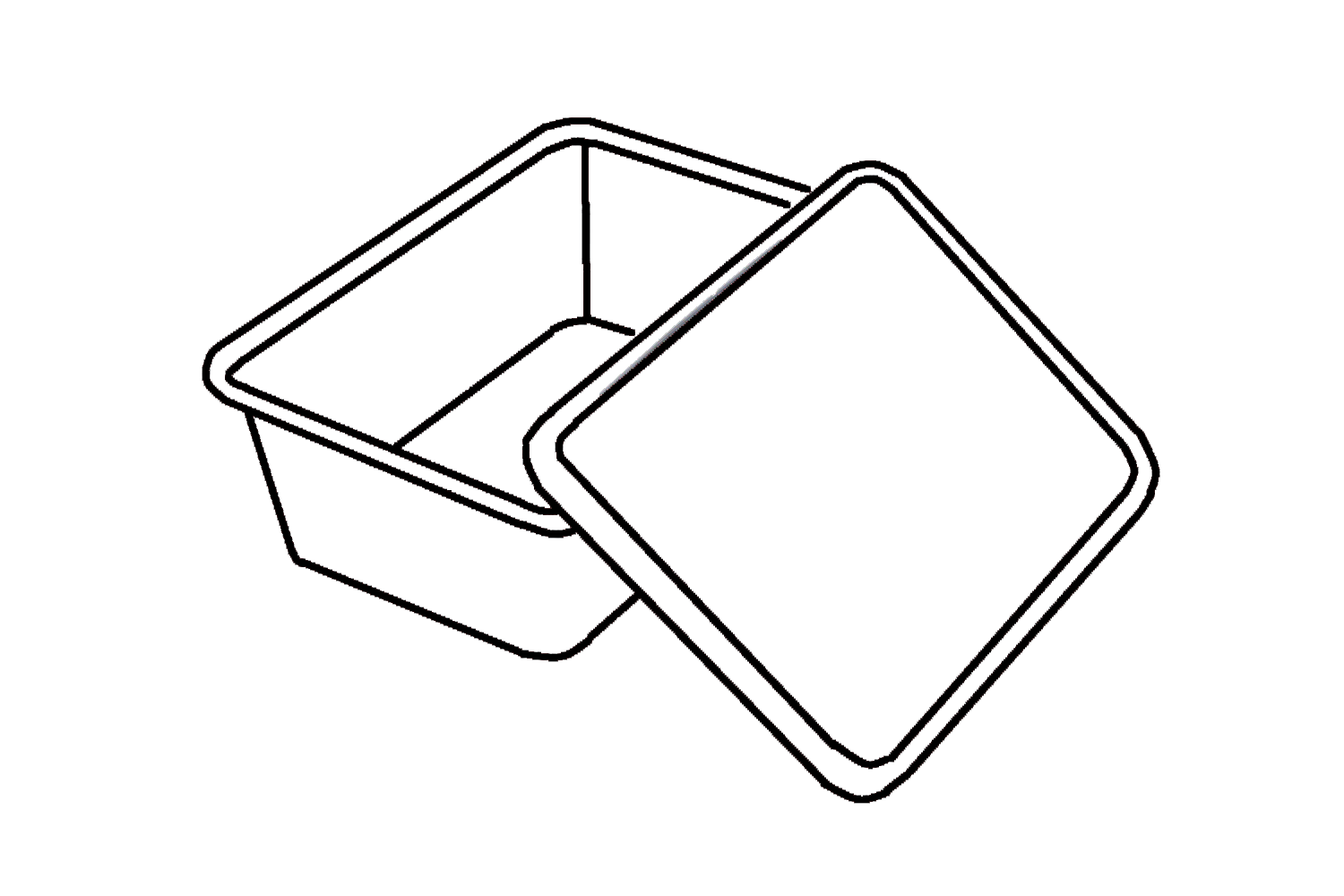 |
|  |  | Condiment | 59 | 20 | 0.12 | 0.07 | Non-porous | 0.0021 | 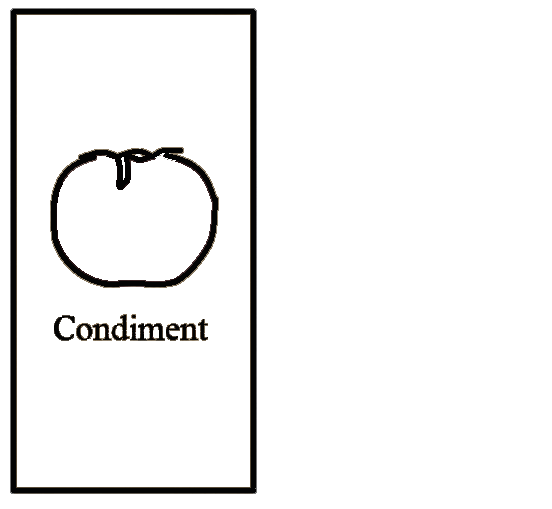 |
|  |  | Disposable gloves for restaurant | 60 | 200 | 0.12 | 0.07 | Non-porous | 0.0021 | 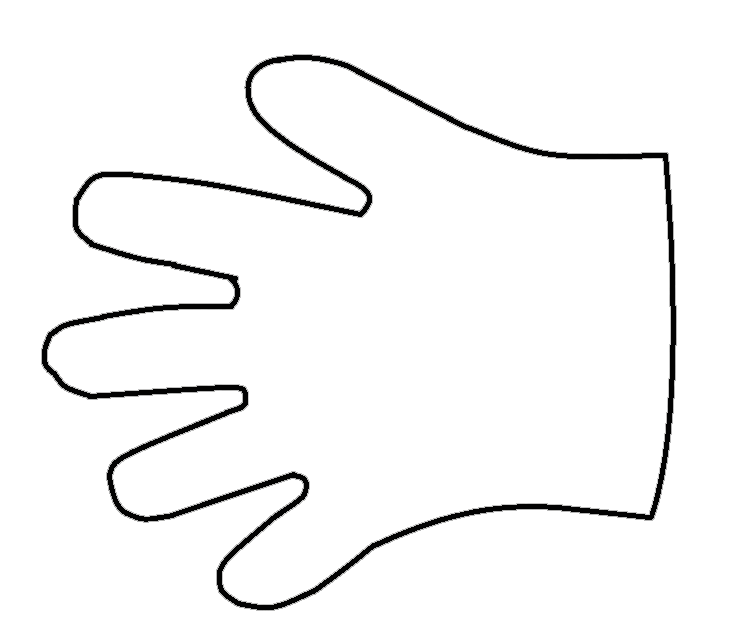 |
|  |  | Tinfoil shell | 61 | 40 | 0.12 | 0.07 | Non-porous | 0.0021 | 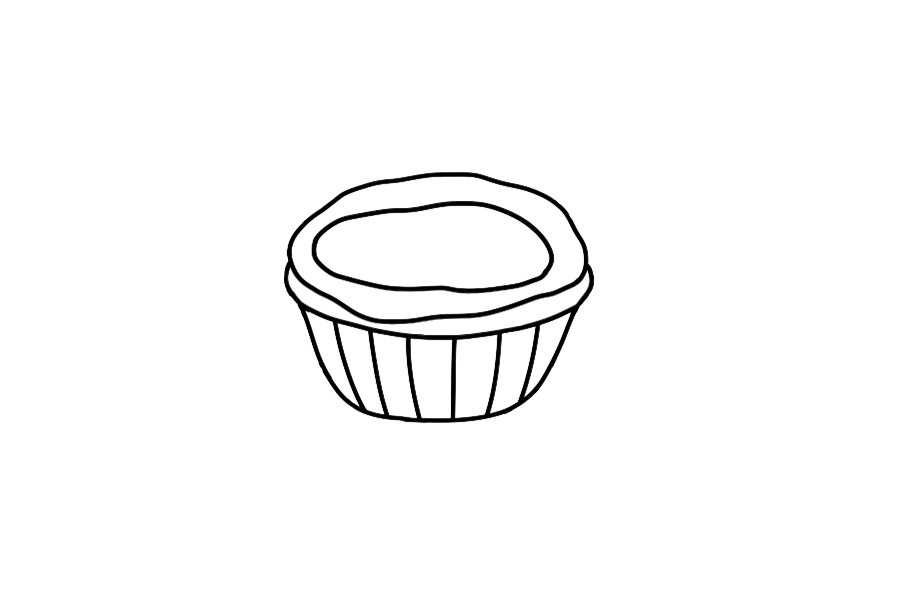 |
|  |  | Straw | 62 | 20 | 0.12 | 0.07 | Non-porous | 0.0021 | 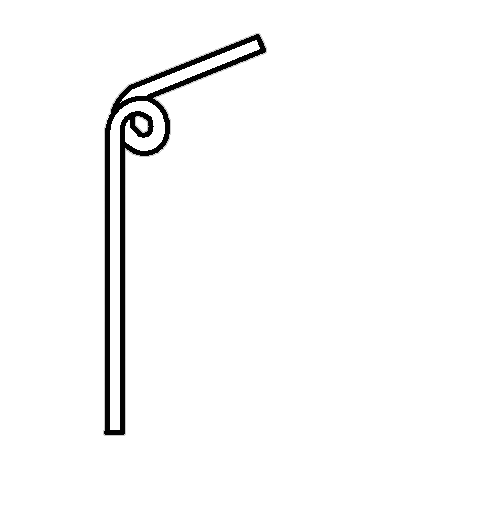 |
|  |  | Plastic waste | 63 | 10 | 0.12 | 0.07 | Non-porous | 0.0021 | 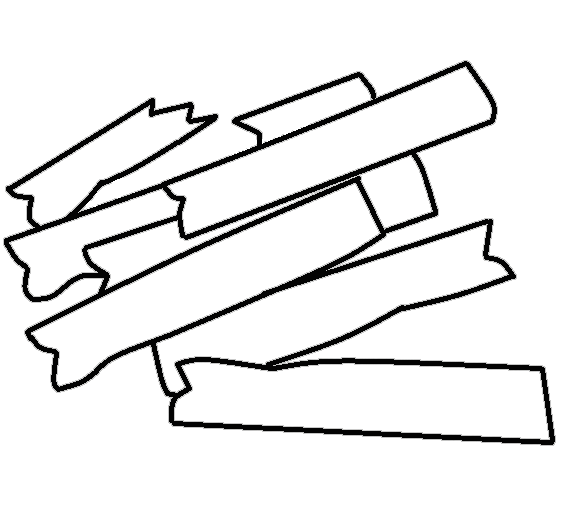 |
|  |  | Chopstick | 64 | 40 | 0.12 | 0.07 | Non-porous | 0.0021 | 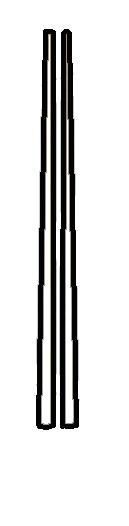 |
|  |  | Food | 65 | 100 | 0.2244 | 0.0013 | Food | -1 | 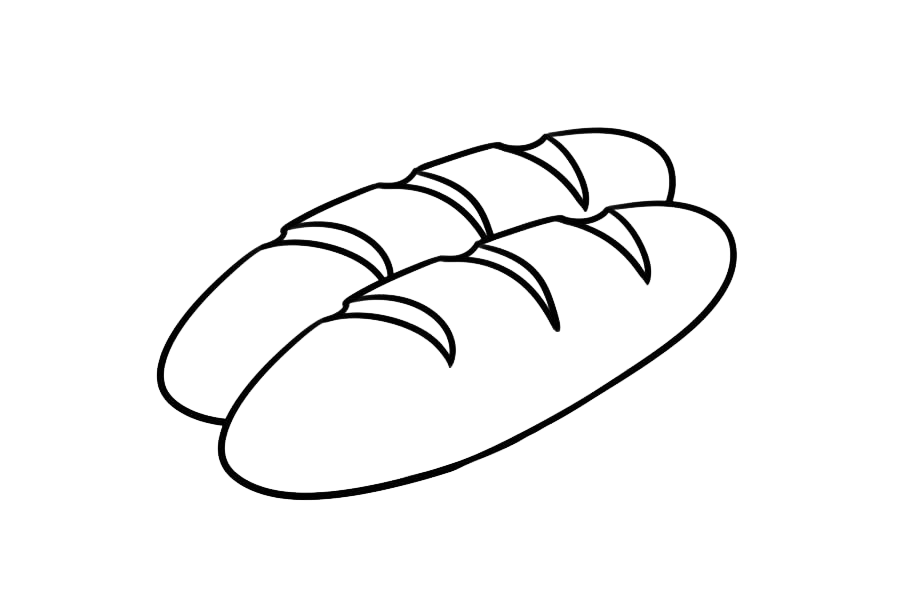 |
|  |  | Food packaging  bag | 66 | 700 | 0.12 | 0.07 | Non-porous | 0.0021 | 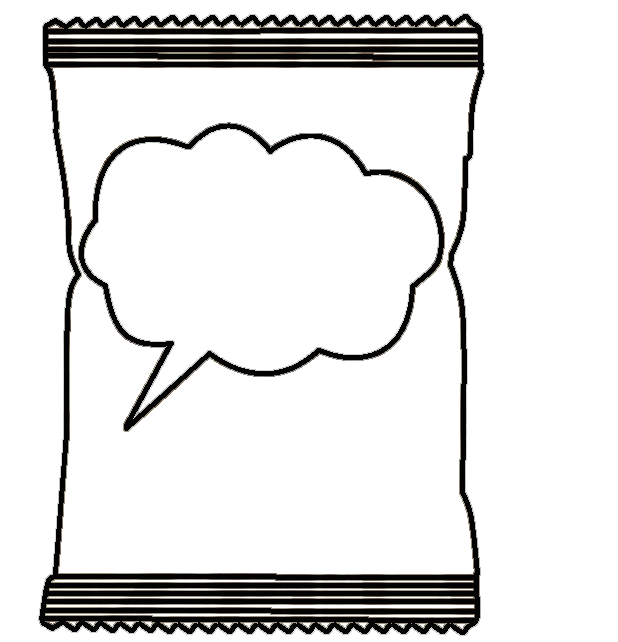 |
|  |  | Signboard | 67 | 100 | 0.6675 | 0.0149 | Paper/tissue | 0.008 | 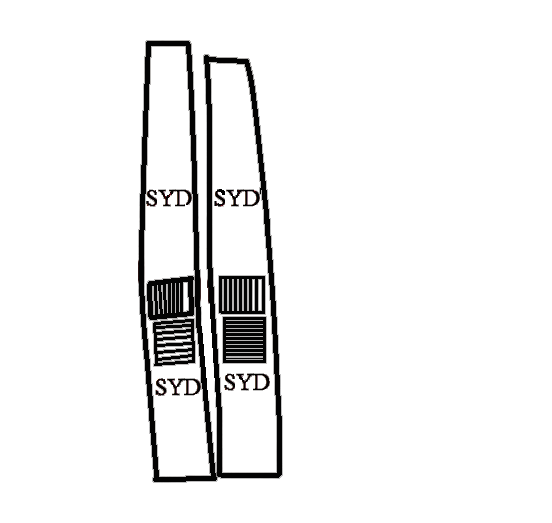 |
|  |  | Boarding pass | 68 | 320 | 0.6675 | 0.0149 | Paper/tissue | 0.008 | 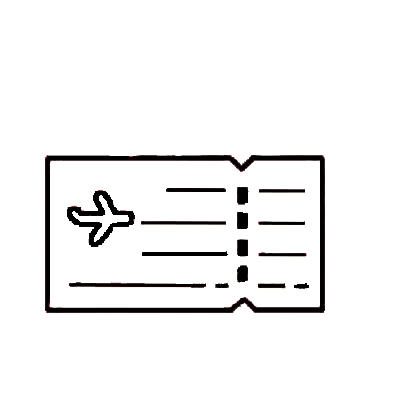 |
|  |  | Spoon | 69 | 30 | 0.048 | 0.2743 | Porcelain | 0.0021 | 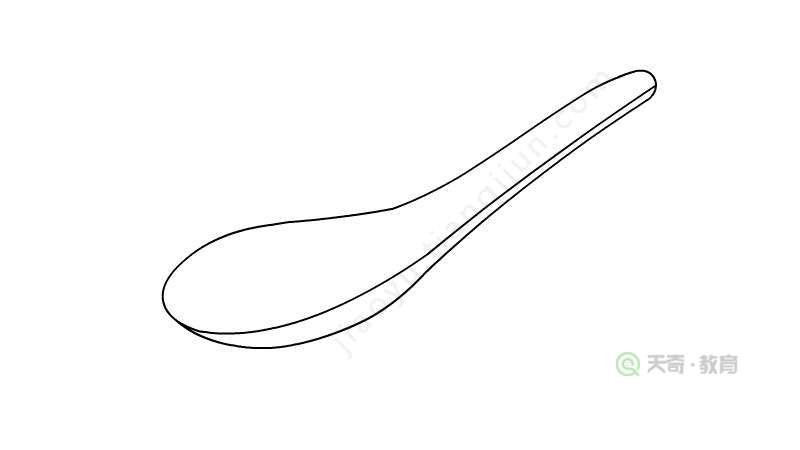 |
|  |  | Bowl  Plate | 70 | 200 | 0.048 | 0.2743 | Porcelain | 0.0021 | 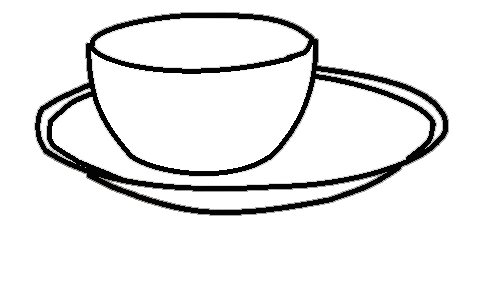 |
|  |  | Can | 71 | 227 | 0.12 | 0.07 | Non-porous | 0.0021 | 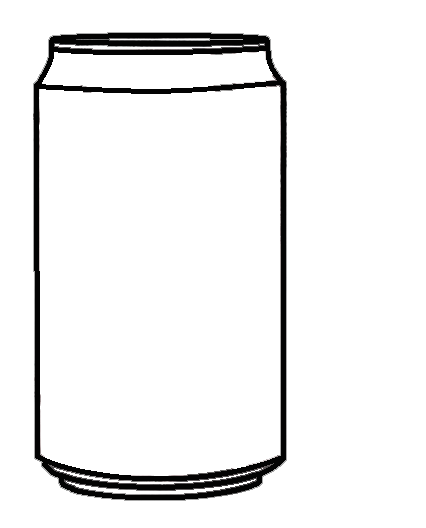 |
|  |  | Snack plate | 72 | 80 | 0.1817 | 0.1817 | undefined material | 0.008 | 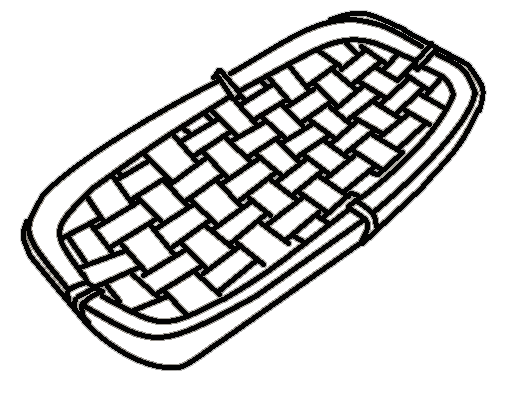 |
|  |  | Toothpick | 73 | 3 | 0.8 | 0.03 | porous | 0.008 | 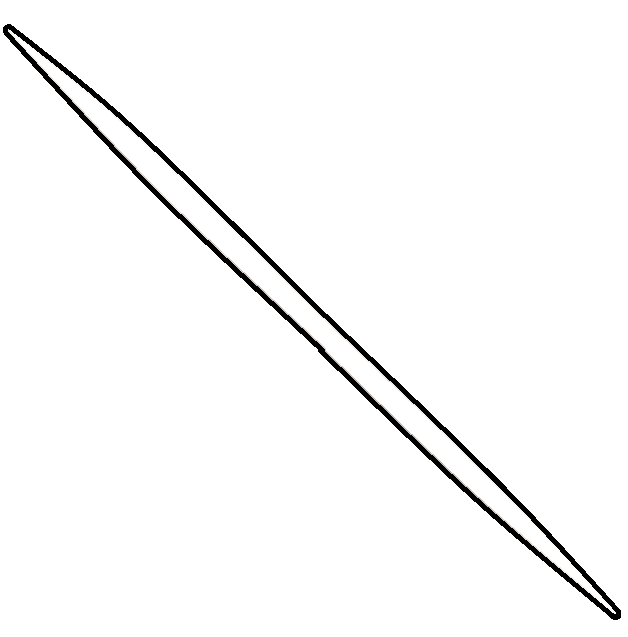 |
|  |  | Seasoning bowl | 74 | 150 | 0.048 | 0.2743 | Porcelain | 0.0021 | 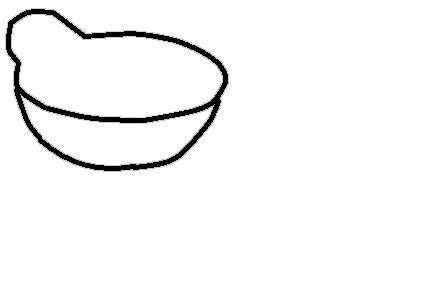 |
|  |  | Serving plate | 75 | 1,500 | 0.12 | 0.07 | Non-porous | 0.0021 | 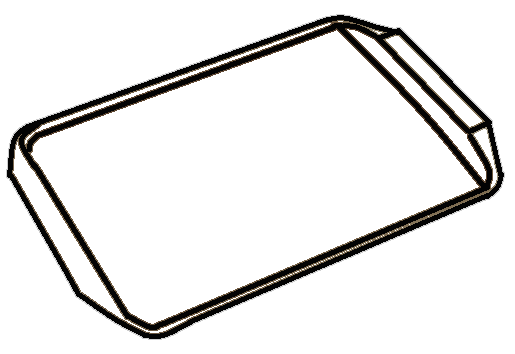 |
| Object for public use for all people  (PP) | | Restaurant chair | 76 | 600 | 0.46 | 0.05 | Porous/non-porous | 0.008 | 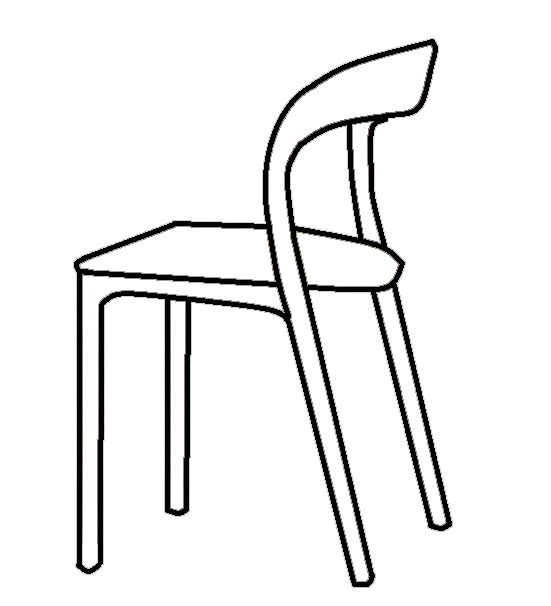 |
|  |  | Restaurant table | 77 | 5,000 | 0.12 | 0.07 | Non-porous | 0.0021 | 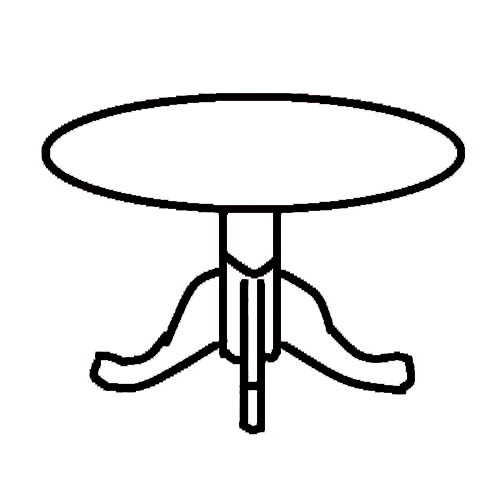 |
|  |  | Meal card | 78 | 160 | 0.12 | 0.07 | Non-porous | 0.0021 | 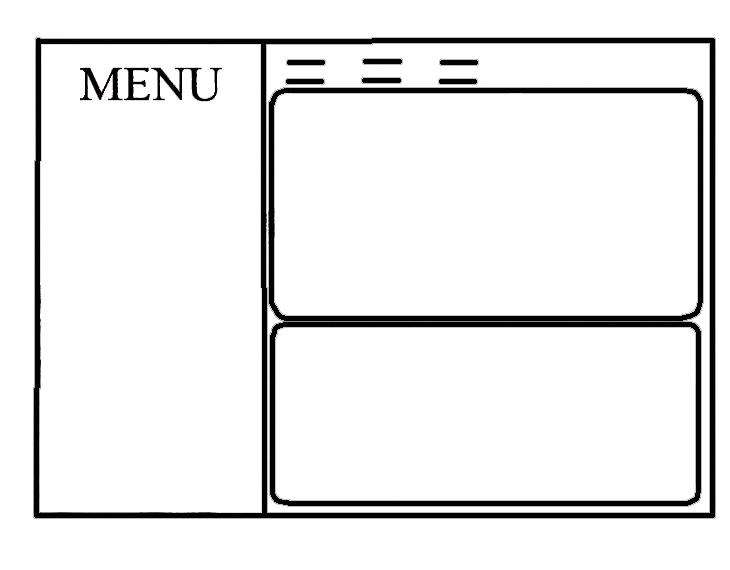 |
|  |  | Restaurant tissue box | 79 | 600 | 0.12 | 0.07 | Non-porous | 0.0021 | 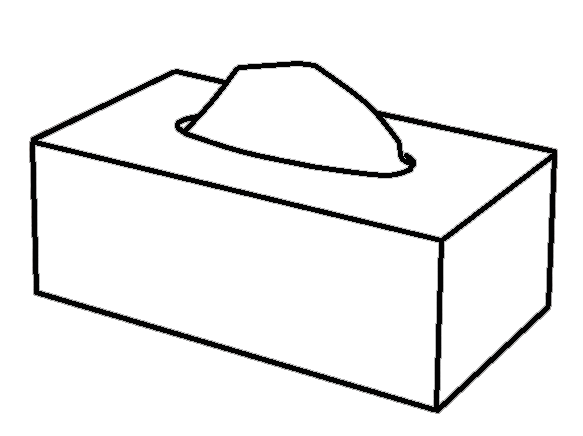 |
|  |  | Seasoning spoon | 80 | 10 | 0.048 | 0.2743 | Porcelain | 0.0021 | 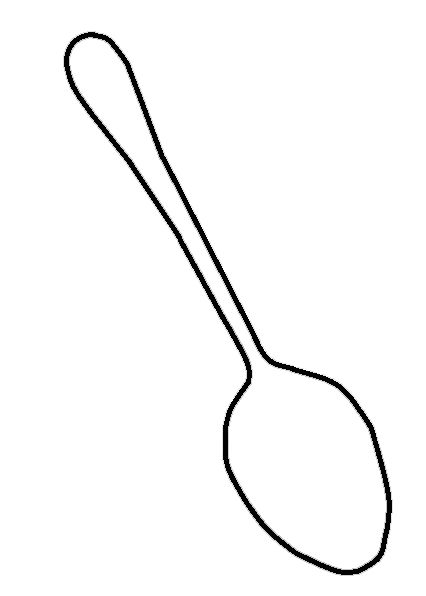 |
|  |  | Seasoning box | 81 | 15 | 0.048 | 0.2743 | Porcelain | 0.0021 | 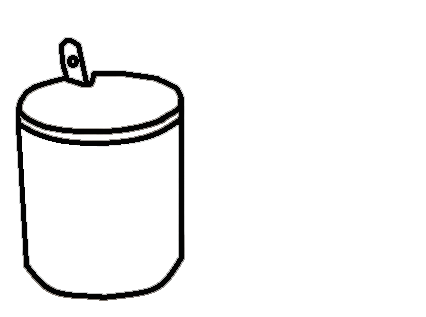 |
|  |  | Cover of seasoning box | 82 | 6 | 0.048 | 0.2743 | Porcelain | 0.0021 |  |
|  |  | Commodities (Placed At Counter 1-6) | 83-88 | 40 | 0.12 | 0.07 | Non-porous | 0.0021 | 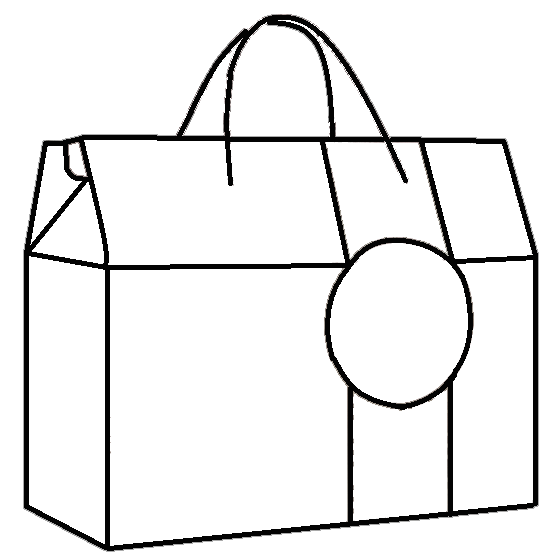 |
|  |  | Stand up sign | 89 | 650 | 0.12 | 0.07 | Non-porous | 0.0021 | 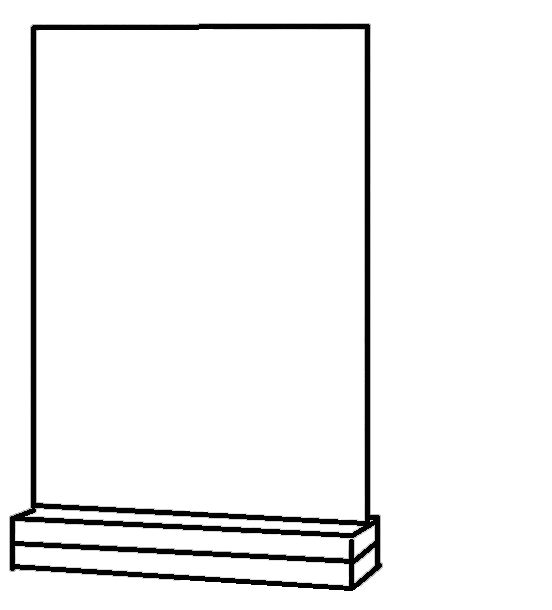 |
|  |  | Escalator handrail | 90 | 18,000 | 0.12 | 0.07 | Non-porous | 0.0021 | 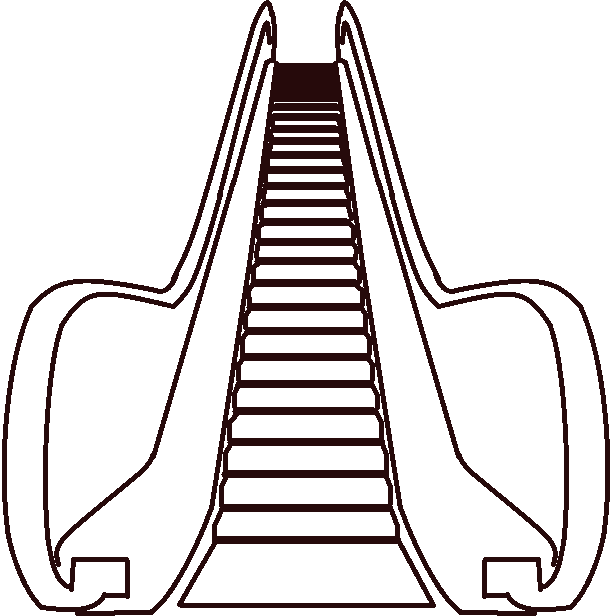 |
|  |  | Escalator glass | 91 | 100,000 | 0.1934 | 0.1803 | Glass | 0.0021 |  |
|  |  | Trolley | 92 | 2,200 | 0.12 | 0.07 | Non-porous | 0.0021 | 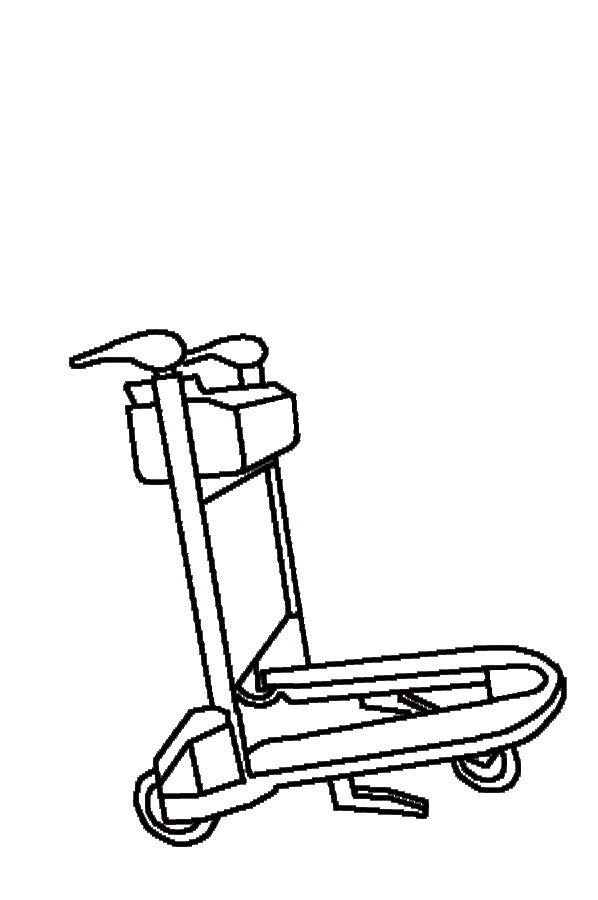 |
|  |  | Luggage tray | 93 | 6,000 | 0.12 | 0.07 | Non-porous | 0.0021 | 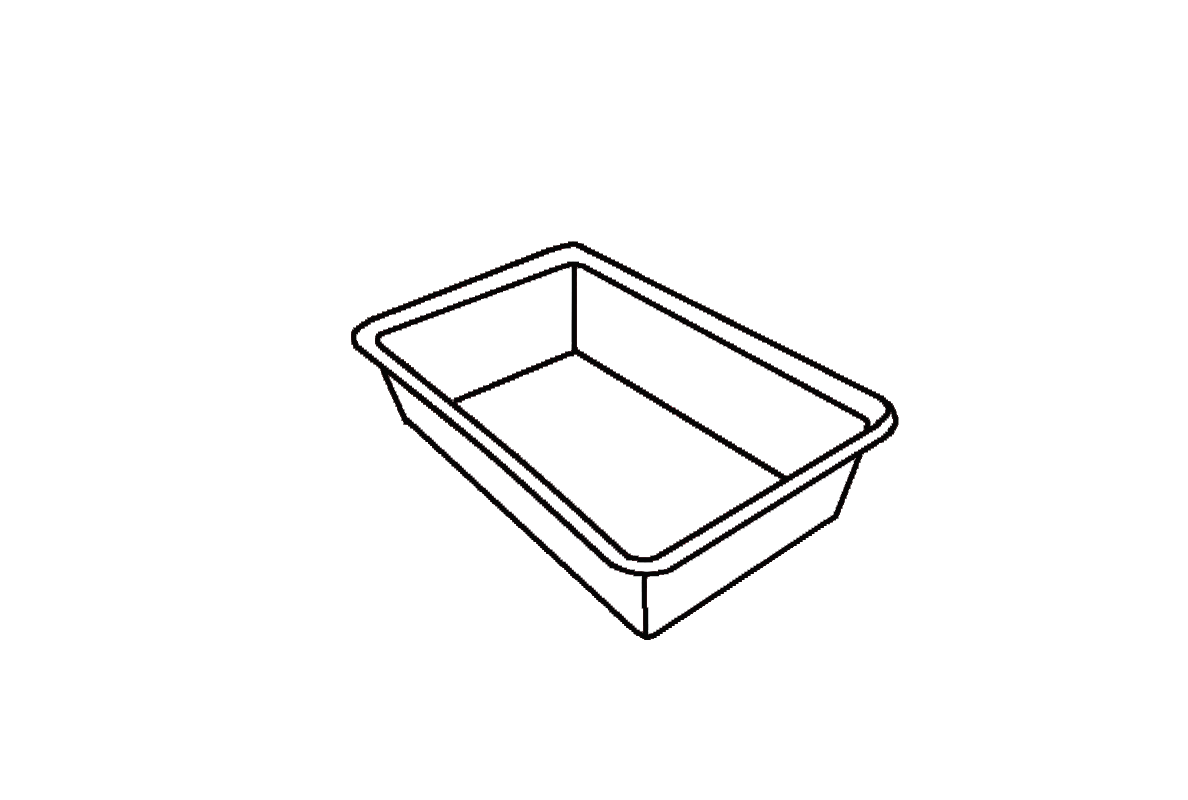 |
|  |  | Self-service check-in machine screen | 94 | 1,000 | 0.1934 | 0.1803 | Glass | 0.0021 | 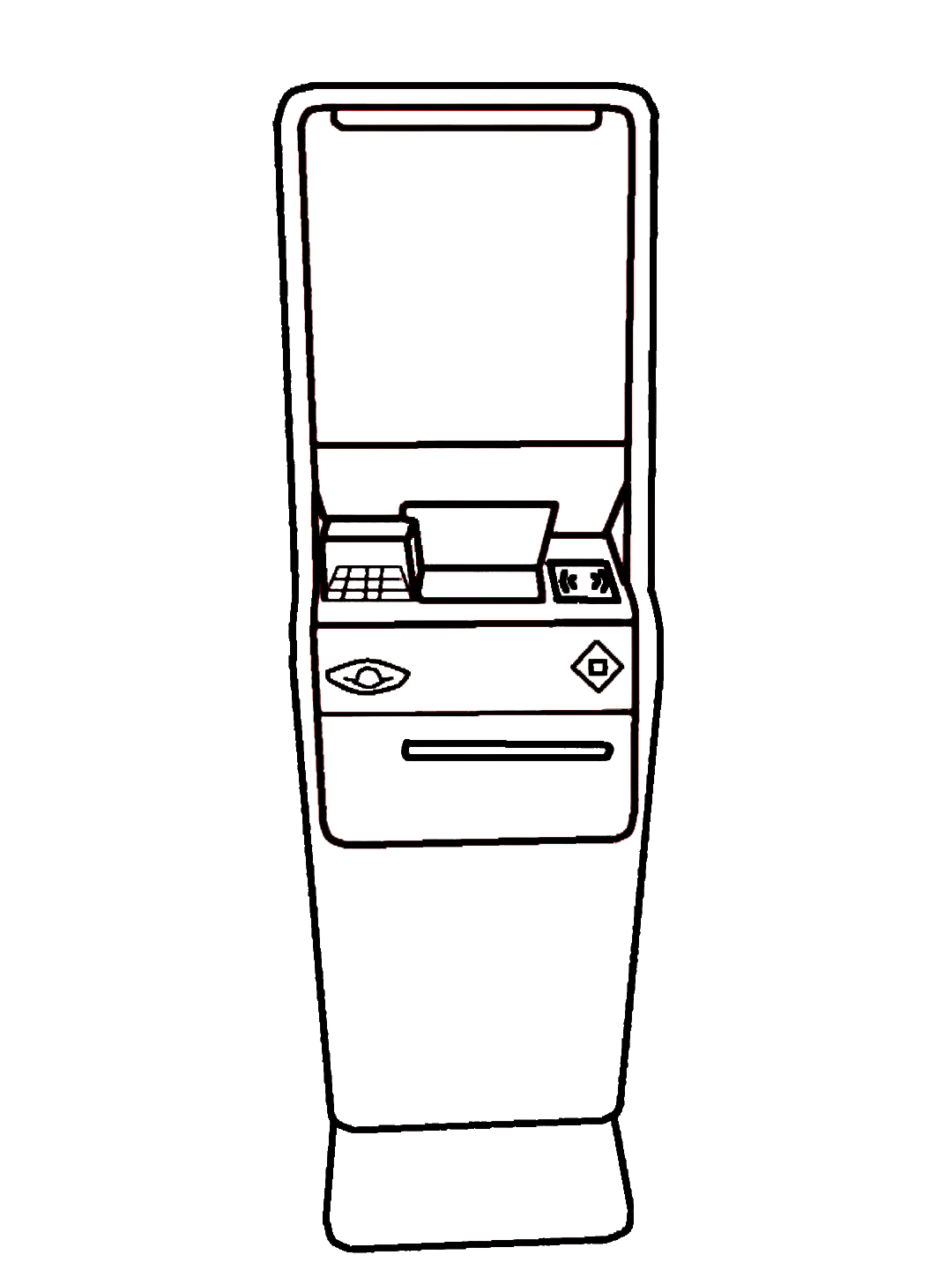 |
|  |  | Self-service check-in machine | 95 | 180 | 0.12 | 0.07 | Non-porous | 0.0021 |  |
|  |  | Table in charging area | 96 | 8,000 | 0.12 | 0.07 | Non-porous | 0.0021 | 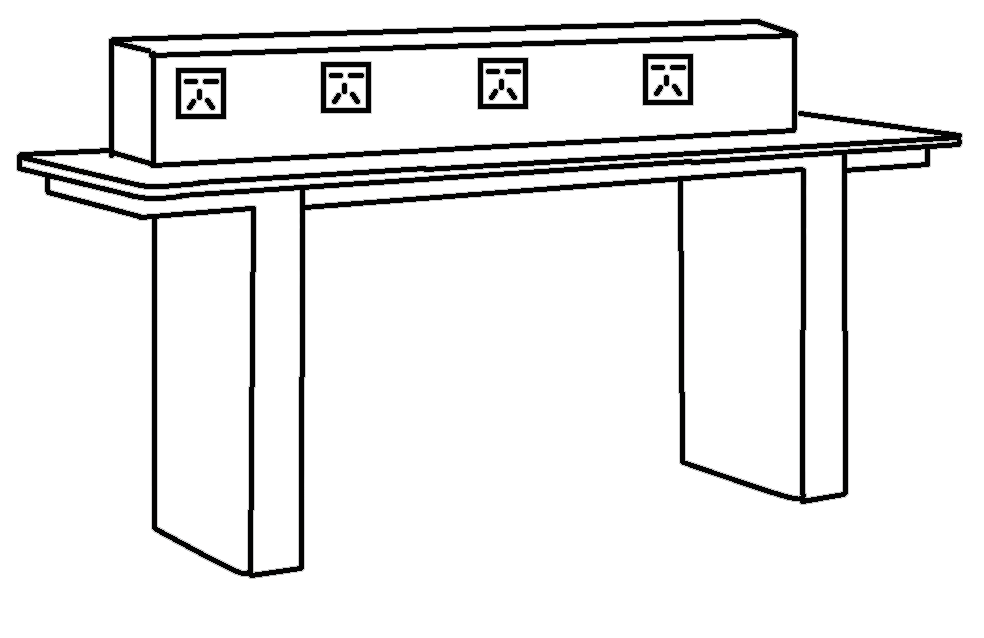 |
|  |  | Hand sanitizer bottle | 97 | 240 | 0.12 | 0.07 | Non-porous | 0.0021 | 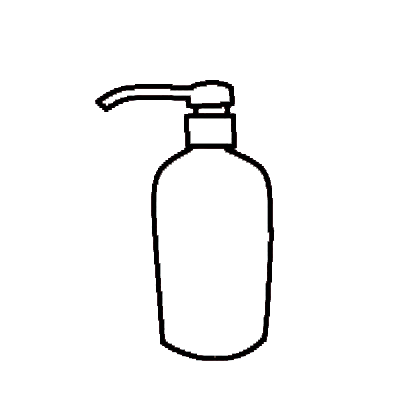 |
|  |  | Pen | 98 | 50 | 0.11 | 0.18 | Pen | 0.0021 | 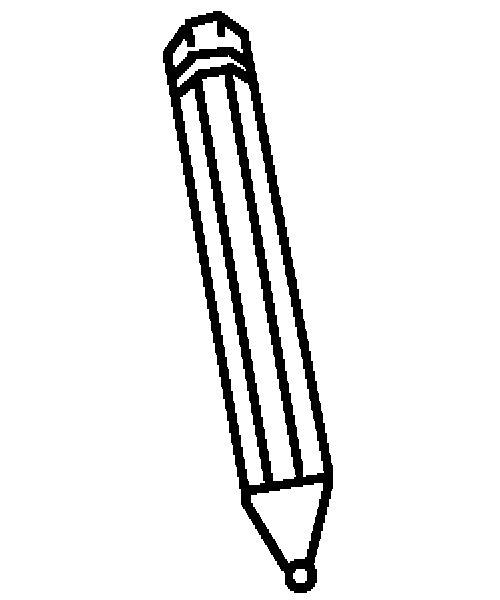 |
|  |  | Check-in counter | 99 | 5,000 | 0.12 | 0.07 | Non-porous | 0.0021 | 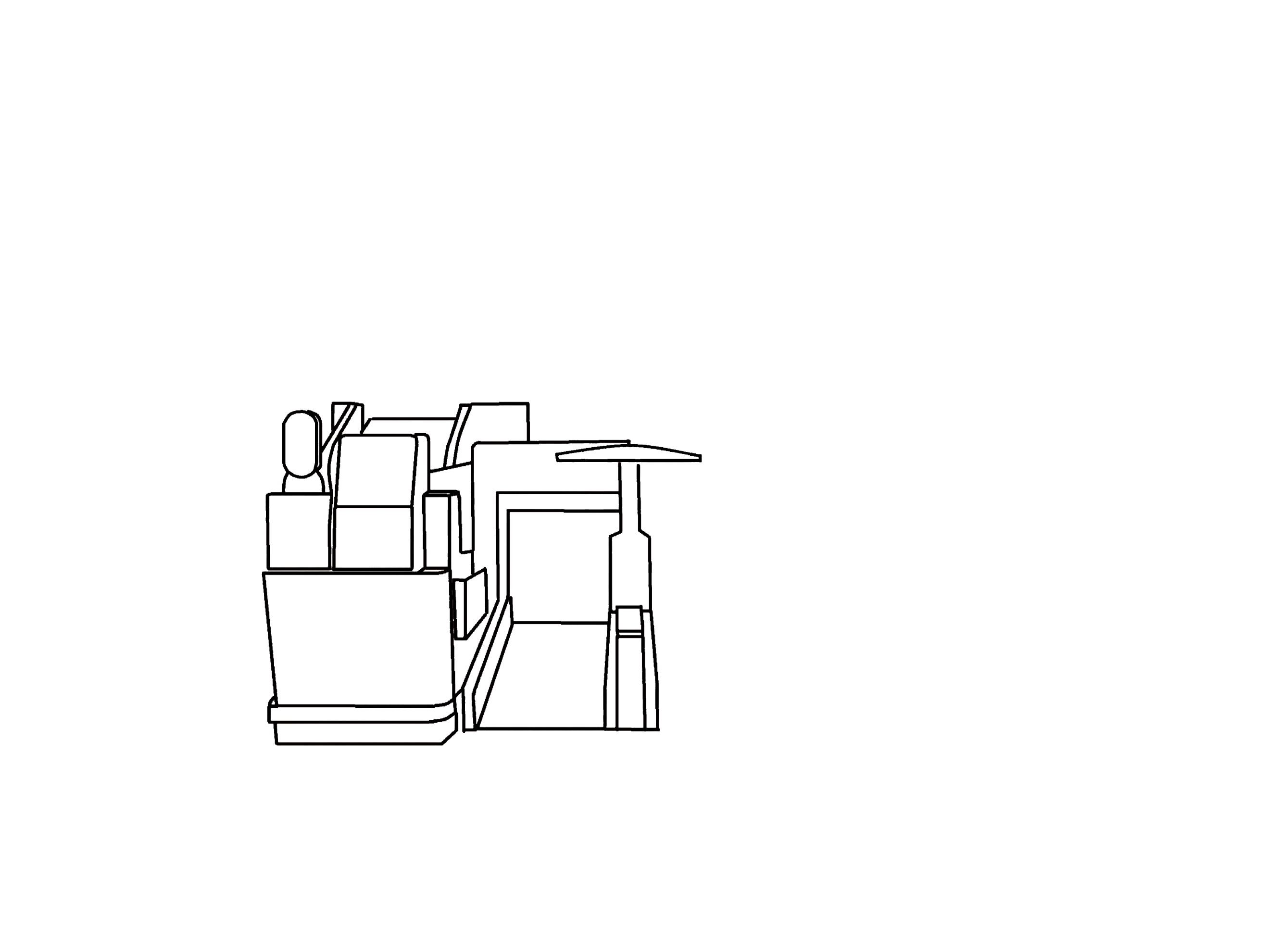 |
|  |  | Fence pole | 100 | 12 | 0.12 | 0.07 | Non-porous | 0.0021 | 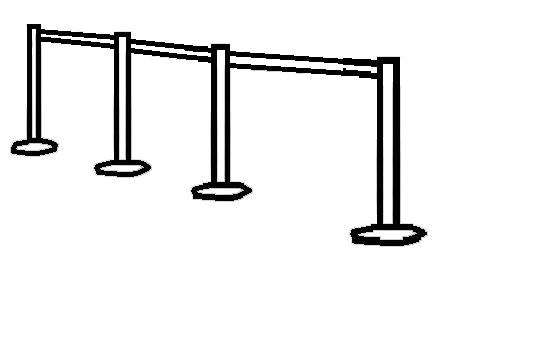 |
|  |  | Transverse band of fence | 101 | 800 | 0.6675 | 0.0149 | Fabric/cloth | 0.008 |  |
|  |  | Chair | 102 | 4,000 | 0.8 | 0.03 | Porous/non-porous | 0.008 | 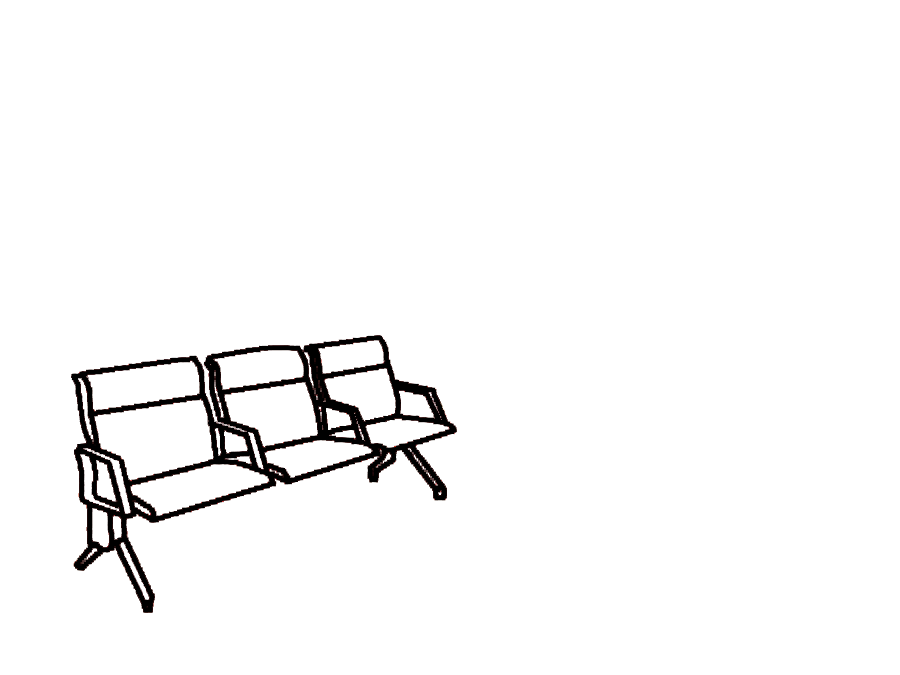 |
|  |  | Airport water dispenser | 103 | 50 | 0.12 | 0.07 | Non-porous | 0.0021 | 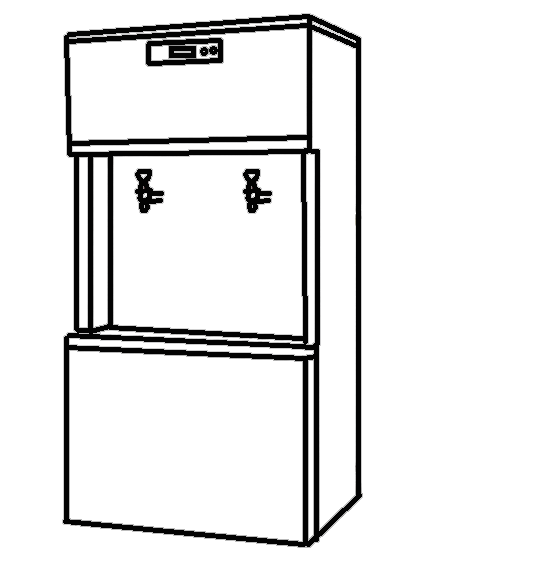 |
|  |  | Trash can | 104 | 1,500 | 0.12 | 0.07 | Non-porous | 0.0021 | 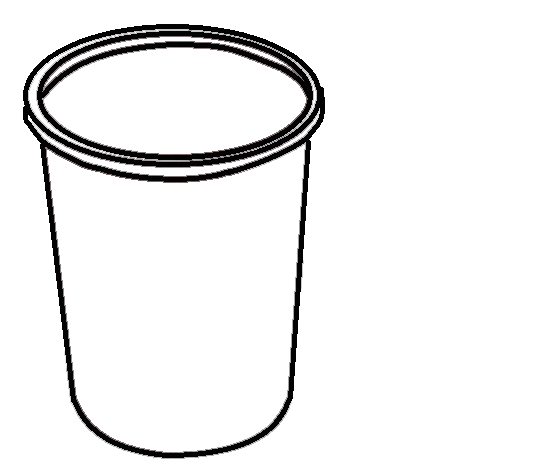 |
|  |  | Restaurant front desk | 105 | 6,000 | 0.12 | 0.07 | Non-porous | 0.0021 | 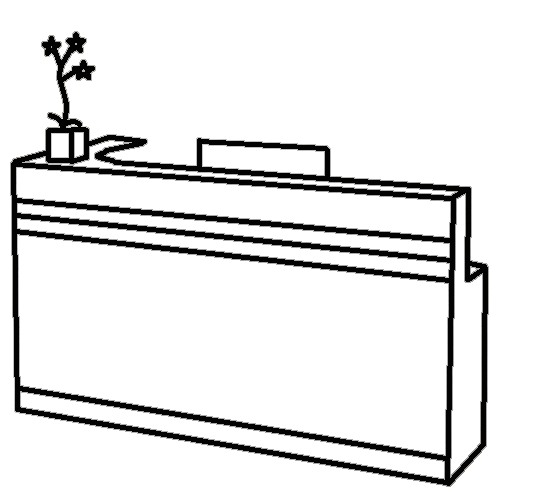 |
|  |  | Boarding gate counter | 106 | 2,400 | 0.12 | 0.07 | Non-porous | 0.0021 | 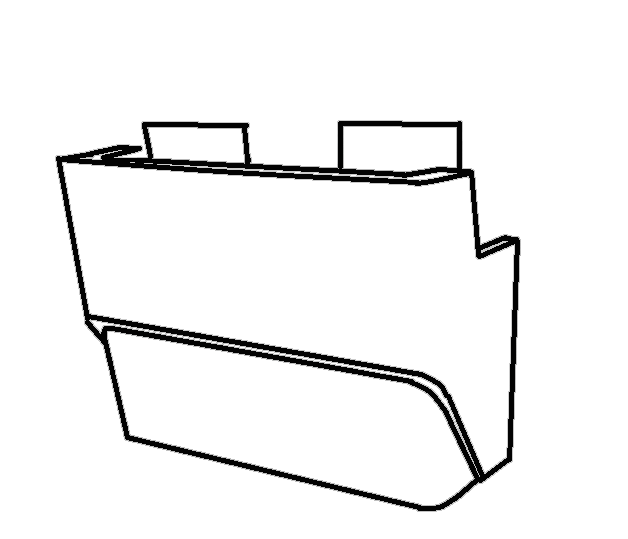 |
|  |  | Wall | 107 | 5,000 | 0.12 | 0.07 | Non-porous | 0.0021 | 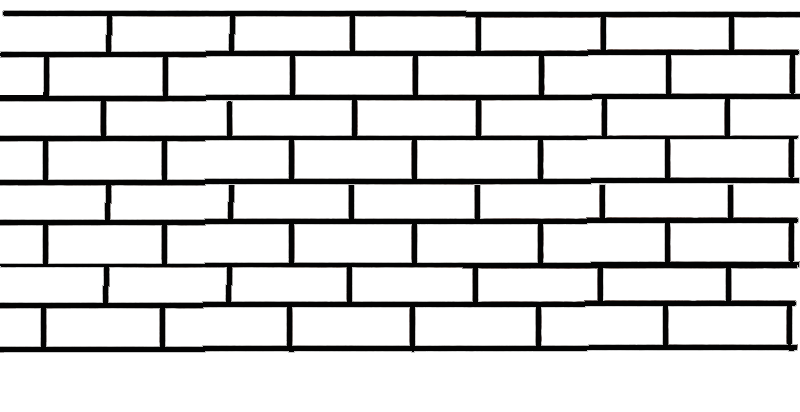 |
|  |  | Settlement counter | 108 | 6,000 | 0.12 | 0.07 | Non-porous | 0.0021 | 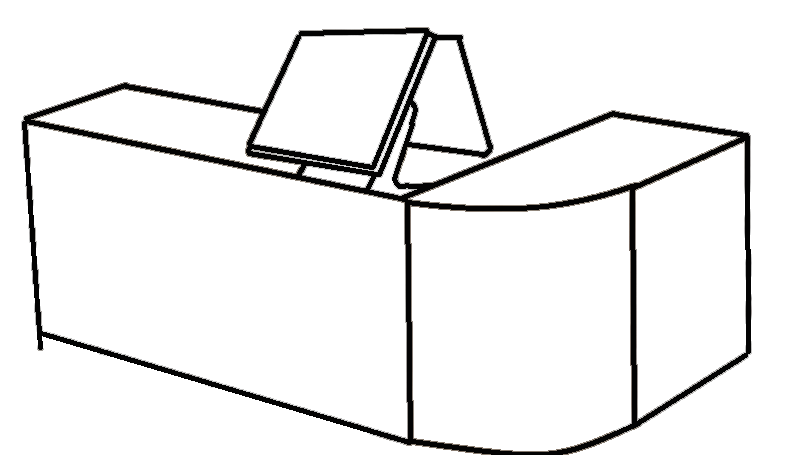 |

^1^Area: The effective touchable surface area of the item; ^2^Transfer rate: The transfer rate of the virus between the hand and the surface refers to the proportion of the virus that is transferred from the hand to the surface or from the surface to the hand upon contact; ^3^Inactivation rate: The rate at which viruses become inactive on the surface.

(All transfer rates between hand and surface were obtained from the literatures [1-9]; The inactivation rate comes from the literature [10-12].

**Reference**

1. Zhang N, Wang P, Miao T, Chan PT, Jia W, Zhao PC, Su B, Chen X, Li Y. Real human surface touch behavior based quantitative analysis on infection spread via fomite route in an office. Building and Environment. 2021; 191:107578.
2. Lopez GU. Transfer of microorganisms from fomites to hands and risk assessment of contaminated and disinfected surfaces. The University of Arizona 2013.
3. Mokhtari A, Jaykus LA. Quantitative exposure model for the transmission of norovirus in retail food preparation. International Journal of Food Microbiology. 2009; 133:38-47.
4. Meadow JF, Altrichter AE, Kembel SW, Moriyama M, O’Connor TK, Womack AM, Brown GZ, Green JL, Bohannan BJM. Bacterial communities on classroom surfaces vary with human contact. Microbiome. 2014; 2:7.
5. Rheinbaben FV, Schünemann S, Groß T, Wolff MH. Transmission of viruses via contact in a household setting: experiments using bacteriophage φX174 as a model virus. Journal of Hospital Infection. 2000; 46:61-6.
6. Fujimura KE, Demoor T, Rauch M, Lynch SV. House dust exposure mediates gut microbiome lactobacillus enrichment and airway immune defense against allergens and virus infection. Proceedings of the National Academy of Sciences. 2014; 111:805-10.
7. Lopez GU, Kitajima M, Havas A, Gerba CP, Reynolds KA. Evaluation of a disinfectant wipe intervention on fomite-to-finger microbial transfer. Applied and environmental microbiology. 2014; 80:3113-8.
8. Bloomfield SF, Aiello AE, Cookson B, O’Boyle C, Larson EL. The effectiveness of hand hygiene procedures in reducing the risks of infections in home and community settings including handwashing and alcohol-based hand sanitizers. American Journal of Infection Control. 2007; 35:S27-64.
9. Boone SA, Gerba CP. Significance of fomites in the spread of respiratory and enteric viral disease. Applied and environmental microbiology. 2007; 73:1687-96.
10. Cannon JL, Papafragkou E, Park GW, Osborne J, Jaykus LA, Vinjé J. Surrogates for the study of norovirus stability and inactivation in the environment: a comparison of murine norovirus and feline calicivirus. Journal of food protection. 2006; 69(11):2761-2765.
11. Centers for Disease Control and Prevention (CDC). Norovirus worldwide. https://www.cdc.gov/norovirus/worldwide.html. Accessed October 31,2021.
12. Hajime K, David JW, William A. The role of the healthcare surface environment in SARS-CoV-2 transmission and potential control measures. Clinical Infectious Disease. 2020; 72:2052-61.
