# Supplementary material for: Public surface disinfection every 2 hours can reduce the infection risk of norovirus in airports up to 83%
Source: PLoS Comput Biol. 2024 Dec 5;20(12):e1012561. doi: 10.1371/journal.pcbi.1012561 (PMC11620375; doi:10.1371/journal.pcbi.1012561)
Supplement: S5 Table — (DOCX) [file pcbi.1012561.s005.docx]

**Table S5.** Probability and duration of passengers staying in different areas [1].

| Airport area | Probability of stay  (%) | Duration for stay  (minute) |
| --- | --- | --- |
| Manual check-in | 46.9 | 98.5 |
| Self-service check-in | 53.1 | 95.6 |
| Escalator | 97.6 | 98.9 |
| Restaurant | 76.8 | 22.0 |
| Charging area | 76.8 | 90.6 |
| Shopping area | 77.3 | 98.2 |
| Waiting area | 99.5 | 87.3 |
| Boarding area | 100.0 | 100.0 |
| Baggage claim area | 96.6 | 99.7 |

**Reference**

1. Zhuang L, Ding Y, Zhou L, Liu R, Ding J, Wang R, et al. Fomite Transmission in Airports Based on Real Human Touch Behaviors. Buildings. 2023; 13:2582.
