# Supplementary material for: Public surface disinfection every 2 hours can reduce the infection risk of norovirus in airports up to 83%
Source: PLoS Comput Biol. 2024 Dec 5;20(12):e1012561. doi: 10.1371/journal.pcbi.1012561 (PMC11620375; doi:10.1371/journal.pcbi.1012561)
Supplement: S6 Table — (DOCX) [file pcbi.1012561.s006.docx]

**Table S6.** Antibacterial surface.

| Surface | Material | Inactivation rate | | Sketch Map |
| --- | --- | --- | --- | --- |
|  |  | Copper | Copper-nickel |  |
| Restaurant chair | Porous/non-porous | 0. 3252 | 0.0653 | 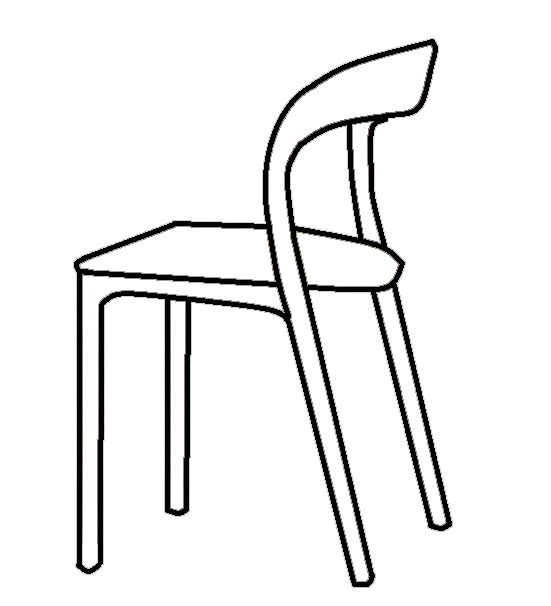 |
| Restaurant table | Non-porous |  |  | 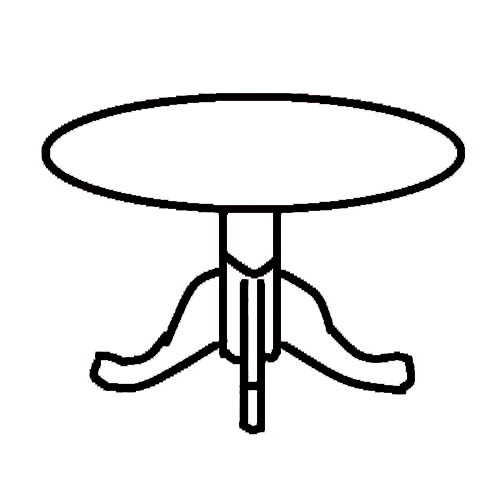 |
| Escalator handrail | Non-porous |  |  | 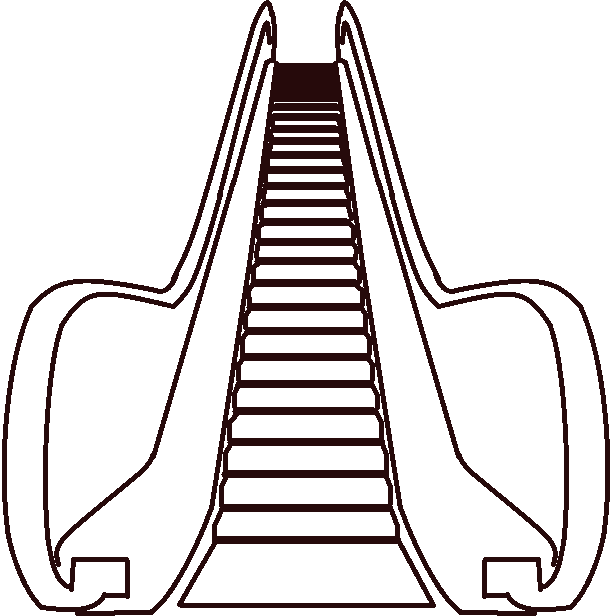 |
| Trolley | Non-porous |  |  | 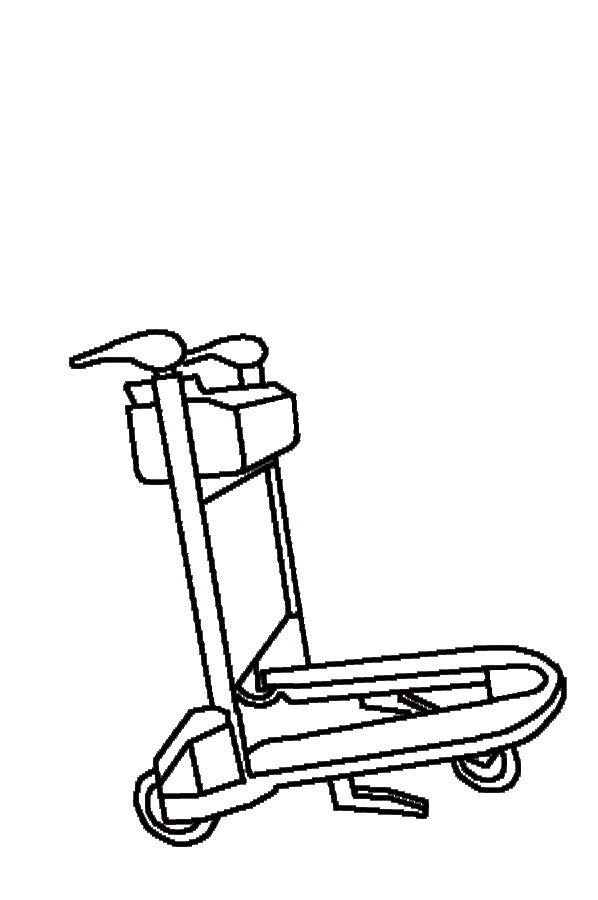 |
| Luggage tray | Non-porous |  |  | 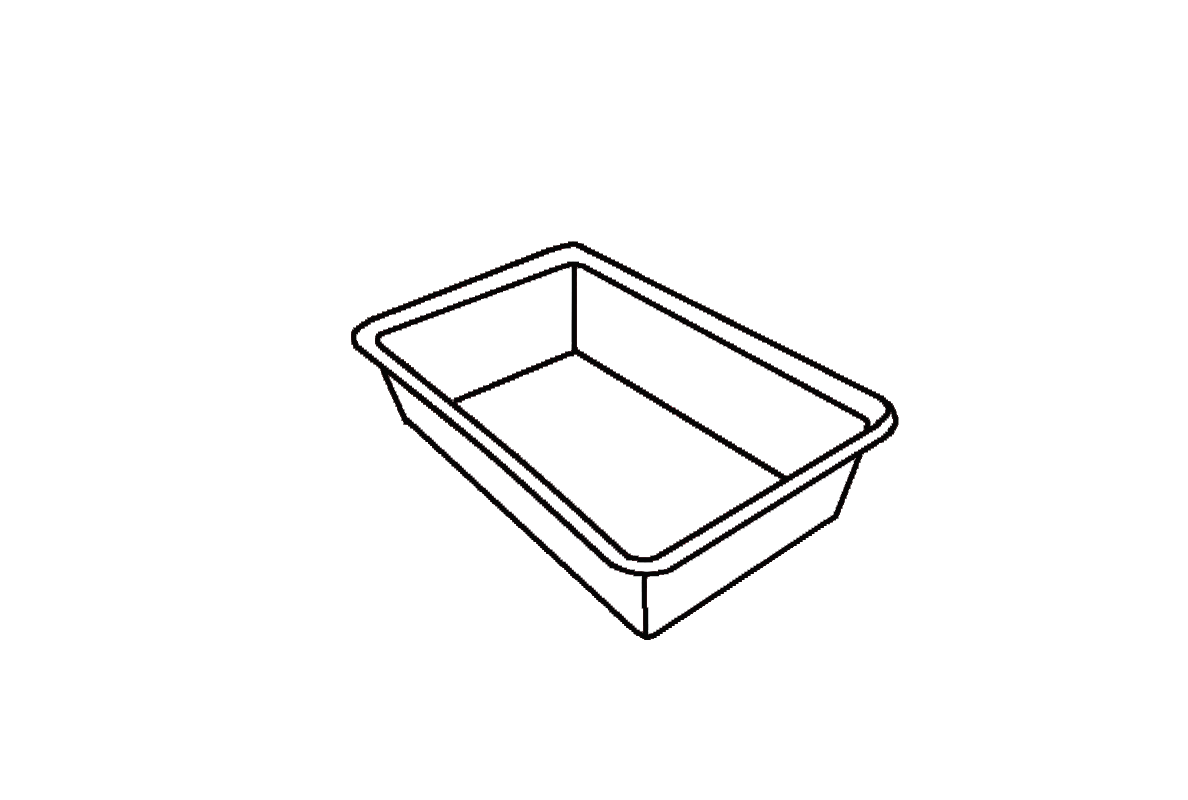 |
| Self-service check-in machine | Non-porous |  |  | 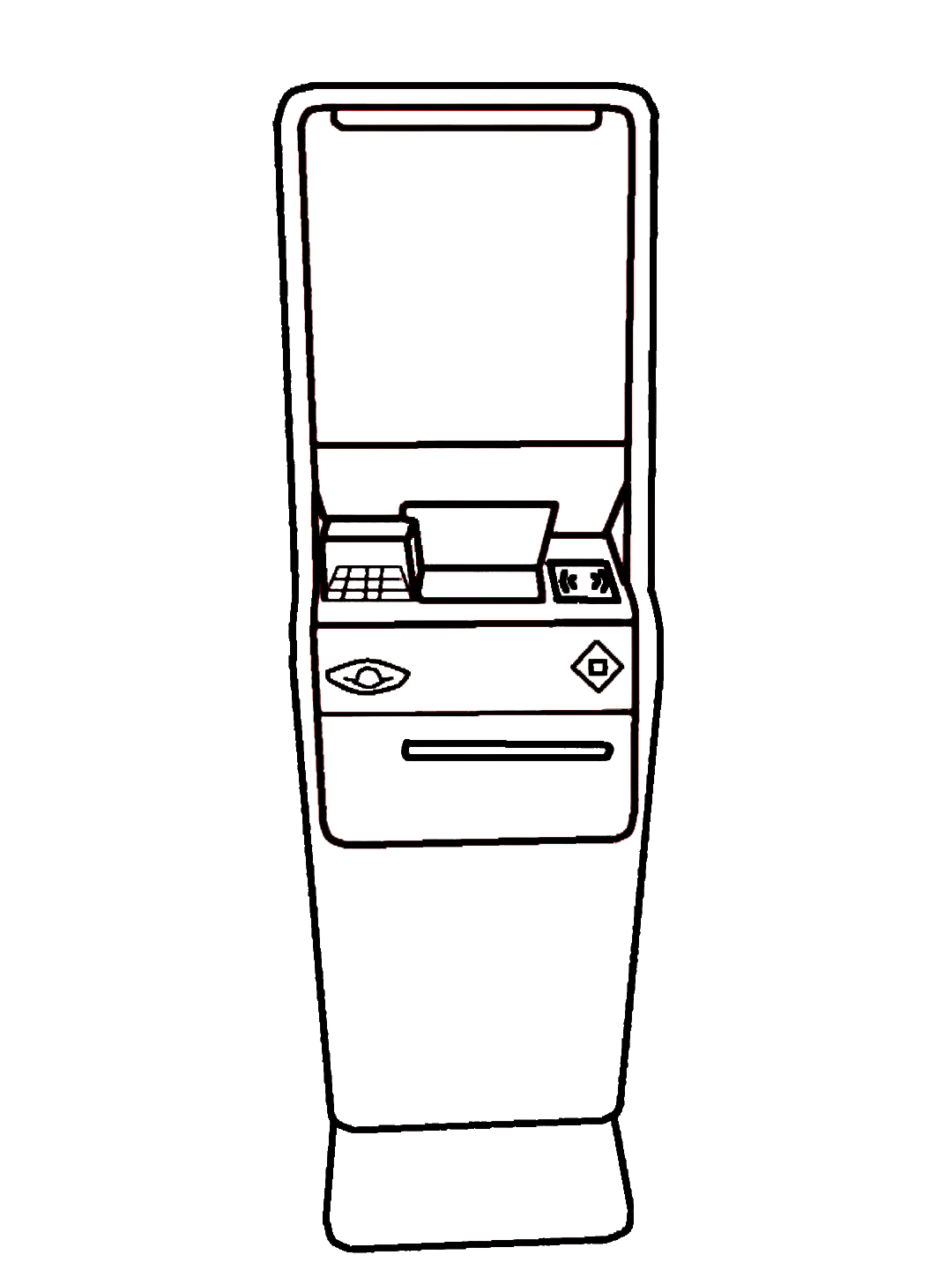 |
| Table in charging area | Non-porous |  |  | 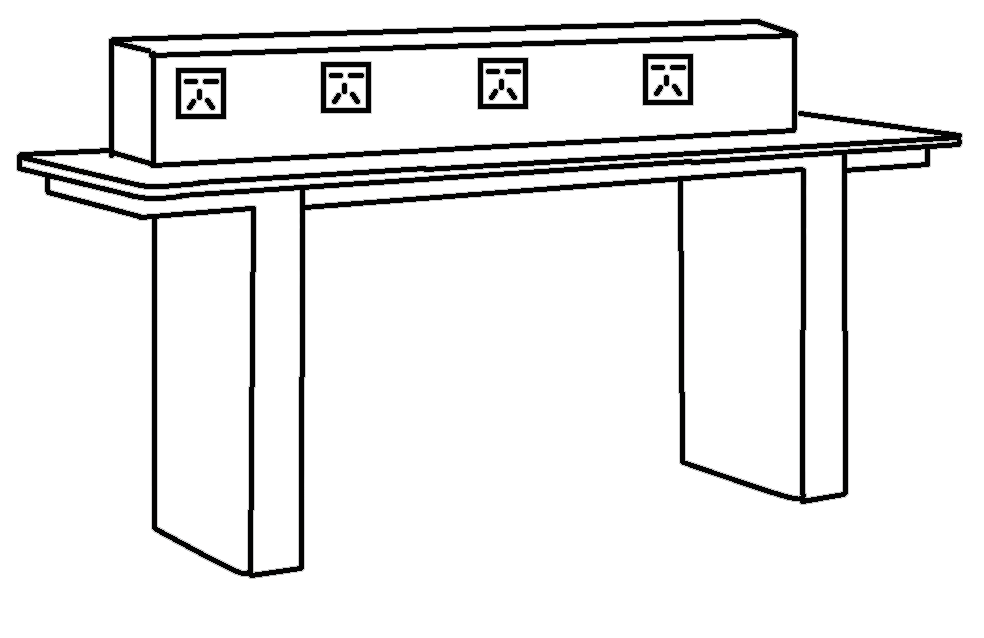 |
| Check-in counter | Non-porous |  |  | 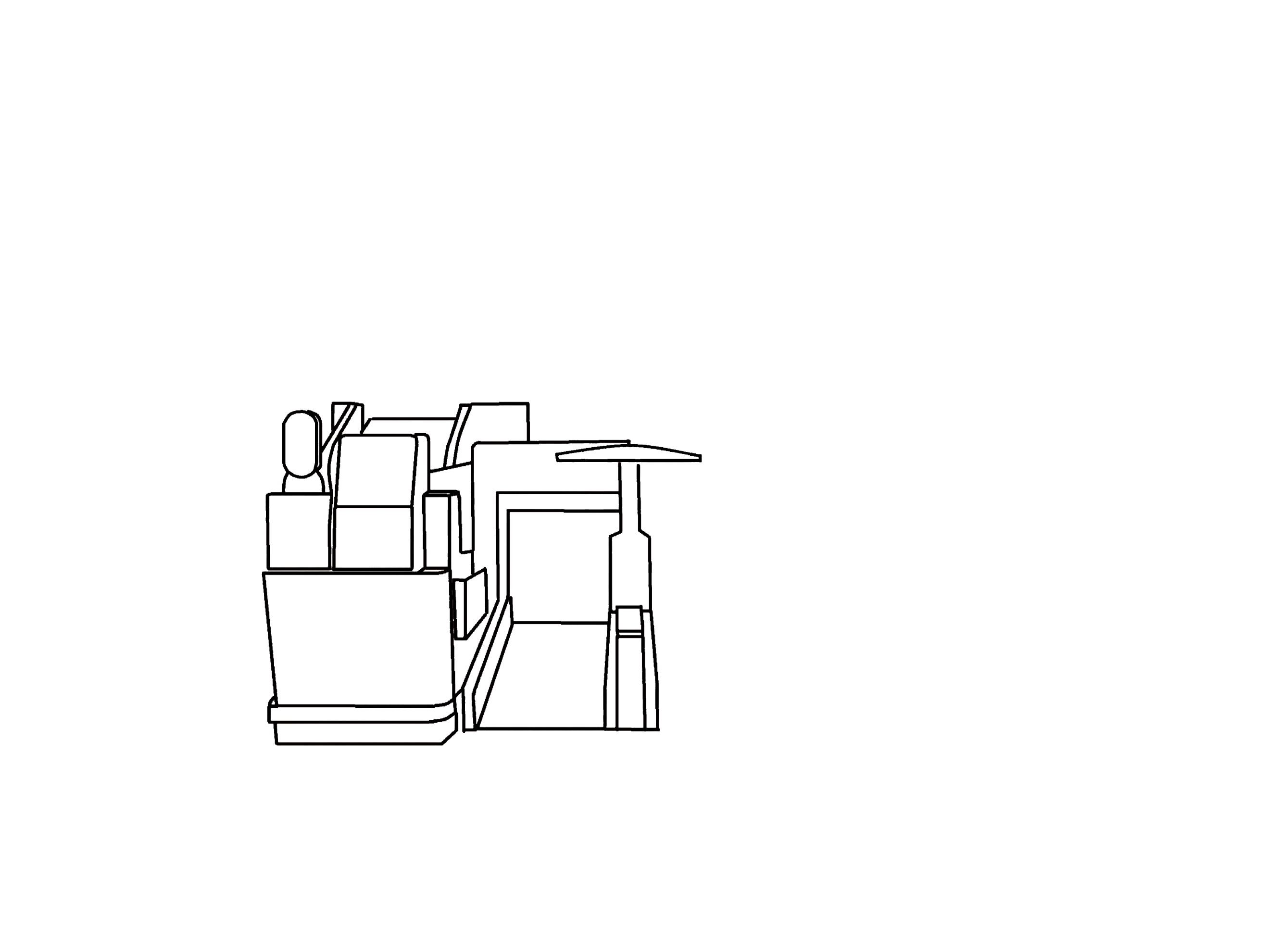 |
| Fence pole | Non-porous |  |  | 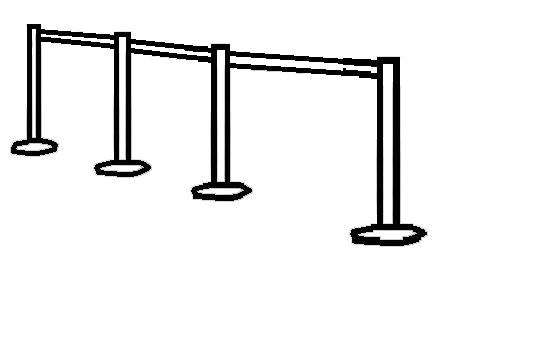 |
| Chair | Porous/non-porous |  |  | 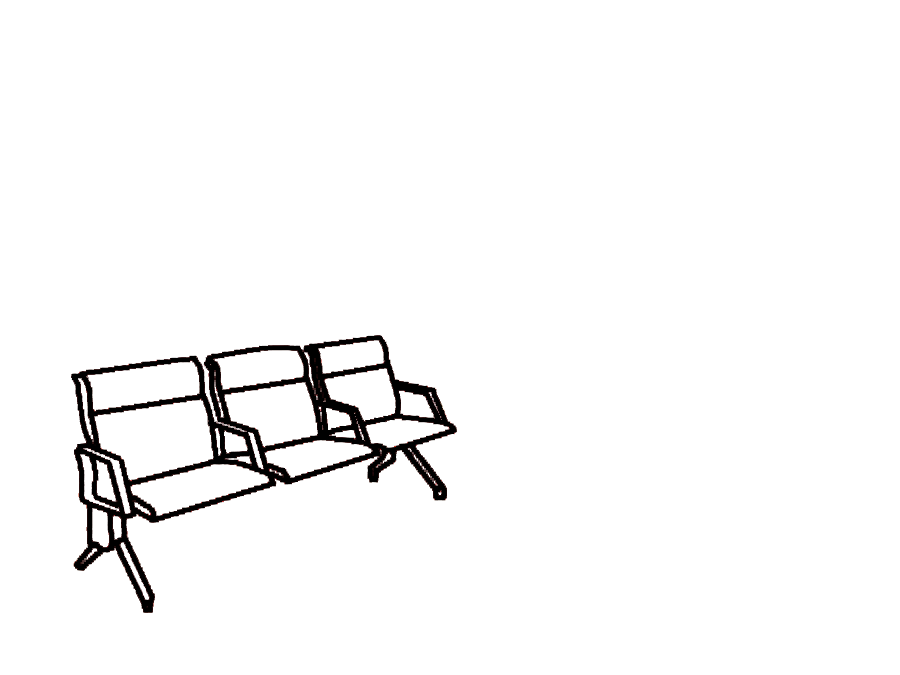 |
| Trash can | Non-porous |  |  | 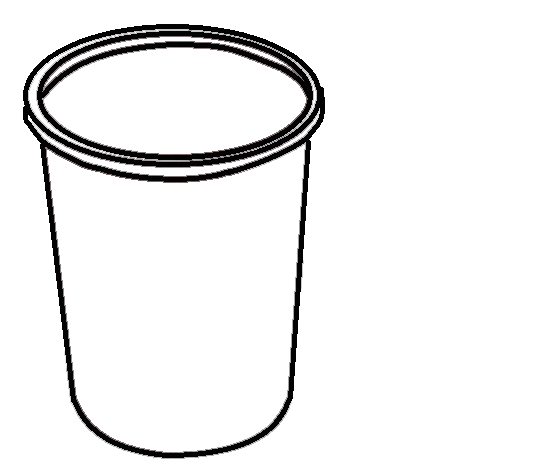 |
| Restaurant front desk | Non-porous |  |  | 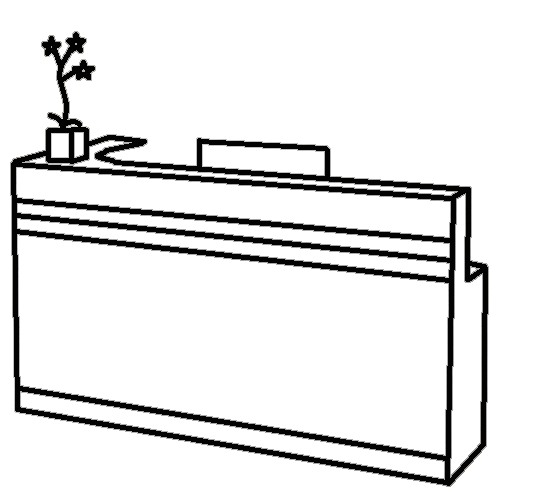 |
| Boarding gate counter | Non-porous |  |  | 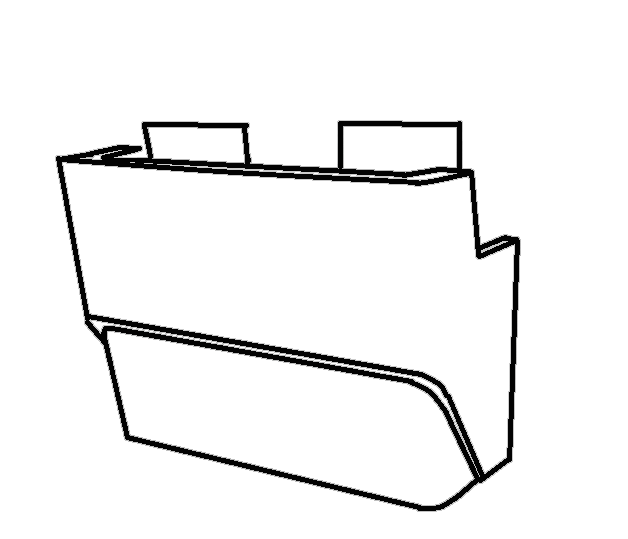 |
| Settlement counter | Non-porous |  |  | 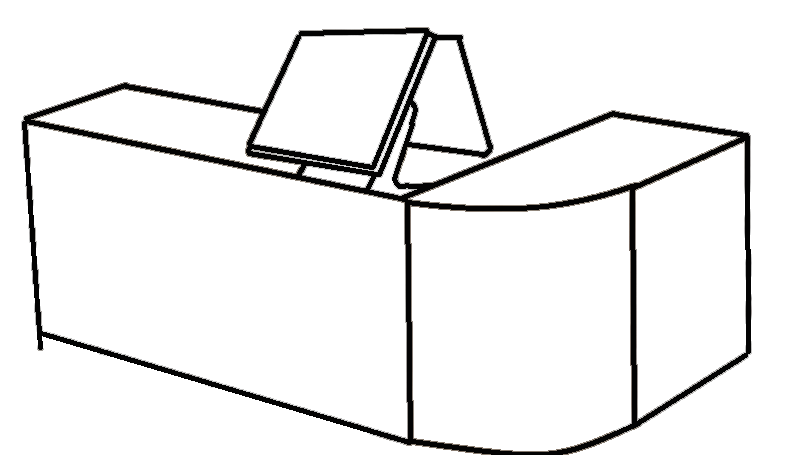 |
