# Supplementary material for: Public surface disinfection every 2 hours can reduce the infection risk of norovirus in airports up to 83%
Source: PLoS Comput Biol. 2024 Dec 5;20(12):e1012561. doi: 10.1371/journal.pcbi.1012561 (PMC11620375; doi:10.1371/journal.pcbi.1012561)
Supplement: S1 Text — (DOCX) [file pcbi.1012561.s007.docx]

**Appendix S1**

**Questionnaire on epidemic prevention and control behavior of airport personnel**

Dear passengers:

To support the prevention and control of infectious diseases, this questionnaire aims to gather information regarding your behavior and daily epidemic prevention measures at the airport. The questionnaire will be anonymous, with completion taking approximately 5 minutes. All information collected will be used for academic research purposes only, and all personal information will remain confidential. Thank you for your cooperation and understanding, and we hope you have a happy life.

As a token of appreciation, participants who carefully fill in and submit valid questionnaires will receive a WeChat red envelope worth RMB 20.0. If you have any inquiries regarding this questionnaire, please contact the Southeast University School of Energy and Environment (Name: **, Phone number: ***) or Key Laboratory of Green Built Environment and Energy Efficient Technology Beijing University of Technology (Name: ***, Phone number: ***).

**1.Personal Information**

Q1: Have you ever flown: [single choice] *

| ○Yes | ○No |
| --- | --- |

Q2: Your gender: [single choice] *

| ○Male | ○Female |
| --- | --- |

Q3: Your age: [single choice] *

| ○0-6 ○7-12 ○13-17 ○18-45 ○46-69 ○>69 |
| --- |

Q4. Your education: [single choice] *

| ○Primary school and below | ○Junior high school |
| --- | --- |
| ○Technical secondary school | ○High school |
| ○Junior college | ○University |
| ○Master degree or above |  |

Q5. Your annual income（pre-tax，RMB）: [single choice] *

| ○50 thousand and below | ○50-100 thousand |
| --- | --- |
| ○100-200 thousand | ○200-300 thousand |
| ○300-500 thousand | ○500 thousand and above |
| ○Secrecy |  |

Q6. How many flights have you taken: [single choice] *

| ○1~5 ○5~10 ○10~20 ○20~50 ○>50 |
| --- |

Q7. Your occupation: [fill in the blank] *

Q8. How long do you usually enter the airport before the plane takes off on time? [fill in the blank]*

Q9. How many people were you with on your last flight? [fill in the blank] *

Q10. Where do you usually go if you are delayed on domestic flights? [Multiple choice] *

| ○Waiting area ○Restaurant ○Shopping ○VIP lounge ○Charging area ○Other areas |
| --- |

Q11. Where do you usually go if you are delayed on international flights? [Multiple choice] *

| ○Haven't taken an international flight ○Duty-free store ○Waiting area ○Restaurant |
| --- |
| ○Shopping ○VIP lounge ○Charging area ○Other areas |

**2.Stay in various places at the airport**

Q12. Your last check-in method: [single choice] *

| ○Manual check-in | ○Self-service check-in |
| --- | --- |

Q13. Your Manual check-in time (Including the time of queuing and checking baggage, Unit: minutes): [fill in the blank] *

Q14. Your Self-service check-in time (Including the time of queuing and checking baggage, Unit: minutes): [fill in the blank] *

Q15. How long did it take you from entering the airport to queuing up for security check? (Unit: minutes): [fill in the blank] *

Q16. How long do you stay in the rest area before security check? (Unit: minutes): [fill in the blank] *

Q17. How long do you stay in the rest area after security check? (Unit: minutes): [fill in the blank] *

Q18. How long do you stay in the charging area? (Unit: minutes): [fill in the blank] *

Q19. The time you spent in the security process (including queuing, Unit: minutes): [fill in the blank] *

Q20. How long do you eat in the airport restaurant (Unit: minutes): [fill in the blank] *

Q21. How long do you stay in the store (shopping) (Unit: minutes): [fill in the blank] *

Q22. How long do you stay at the baggage claim area (Unit: minutes): [fill in the blank] *

Q23. How many times have you washed your hands (including disinfectant) since you last entered the airport (terminal), How often do you go to the bathroom? (Unit: time)

**3.Close contact behavior**

Q24. How often do you talk to friends during check-in (including waiting for check-in)?[Sliding bar] *

Q25. In the seating area of the pre security lounge, the percentage of time you spend talking with friends in the total time in the lounge. [Sliding bar] *

Q26. When you are in the airport lobby before security check, the percentage of time you spend talking with your friends in the total time in the lobby. [Sliding bar] *

Q27. The percentage of time you spend talking to your friends in the security check queue. [Sliding bar] *

Q28. When you are seated in the waiting area after security check, the percentage of time you spend talking with your friends in the total time in the waiting area. [Sliding bar] *

Q29. When you eat in a restaurant (restaurant), the percentage of time you spend talking to your friends in the total dining time. [Sliding bar] *

Q30. When dining in a restaurant (restaurant), the percentage of time you spend talking to the waiter in the total dining time. [Sliding bar] *

Q31. When shopping at the airport store, the percentage of your total shopping time spent talking to friends. [Sliding bar] *

Q32. When shopping at the airport store, the percentage of your total shopping time spent talking to the waiter. [Sliding bar] *

Q33. The percentage of time you spend talking to your friends during baggage collection as a percentage of the total baggage collection time. [Sliding bar] *

**4.Surface touch behavior**

Q34. When taking a plane at the airport this time, while taking the elevator (including escalators and straight stairs), which of the following public surfaces have you touched? [Multiple choice] *

| □Never took the escalator | □Never took the elevator |
| --- | --- |
| □Haven't touched any surface | □Escalator handrail |
| □Elevator external button | □Elevator internal button |
| □Elevator internal handrail | □Other _________________ |

Q35. Have you ever used a luggage cart during this trip? [single choice] *

| ○Yes ○No |
| --- |

Q36. Which of the following public surfaces have you touched in the security check area? [Multiple choice] *

| □Haven't touched any surface | □ID card induction area |
| --- | --- |
| □Check the ticket counter | □Luggage box |
| □Staff body surface | □Other _________________ |

Q37. Which of the following public surfaces have you touched in the toilet? [Multiple choice] *

| □Never been to the toilet | □Haven't touched any surface |
| --- | --- |
| □Toilet pit door | □Close stool |
| □Flush button | □Faucet |
| □Other _________________ |  |

Q38. Which of the following public surfaces did you touch when you took the shuttle bus last time? [Multiple choice] *

| □Never ride | □Haven't touched any surface |
| --- | --- |
| □Shuttle bus window | □Shuttle bus door |
| □Shuttle bus seat | □Handrails in the shuttle bus |
| □Other _________________ |  |

**5.Epidemic prevention measures**

Q39. Your vaccination status: [single choice] *

| ○Have not been vaccinated | ○Have received 1 shot of vaccine |
| --- | --- |
| ○Have received 2 shot of vaccine | ○Have received 3 shot of vaccine |

Q40. Which of the following epidemic prevention measures do you think is the most effective? [Multiple choice] *

| □Wear a surgical mask | □Wash your hands frequently |
| --- | --- |
| □Keep social distance | □Strengthen ventilation |
| □Surface disinfection | □Air disinfection |
| □Vaccination | □Consult a doctor promptly after symptoms of COVID-19 |
| □None of the above |  |

Q41.What do you think is the mode of transmission of the COVID-19[Multiple choice] *
